# Quantitative proteomics identifies and validates urinary biomarkers of rhabdomyosarcoma in children

**DOI:** 10.1186/s12014-023-09401-4

**Published:** 2023-03-14

**Authors:** Na Xu, Yuncui Yu, Chao Duan, Jing Wei, Wei Sun, Chiyi Jiang, Binglin Jian, Wang Cao, Lulu Jia, Xiaoli Ma

**Affiliations:** 1 Medical Oncology Department, Pediatric Oncology Center, Beijing Children’s Hospital, Capital Medical University, National Center for Children’s Health, Beijing Key Laboratory of Pediatric Hematology Oncology, Key Laboratory of Major Diseases in Children, Ministry of Education, No. 56 Nalishi Road, Beijing, 100045 China; 2 Clinical Research Center, Department of Pharmacy, Beijing Children’s Hospital, Capital Medical University, National Center for Children’s Health, Beijing, No. 56 Nanlishi Road, Beijing, 100045 China; 3 Proteomics Research Center, Core Facility of Instruments, Institute of Basic Medical Sciences, Chinese Academy of Medical Sciences and Peking Union Medical College, Beijing, China; 4 Department of Pediatrics, Beijing Friendship Hospital, Capital Medical University, Beijing, China

**Keywords:** Rhabdomyosarcoma, Urinary proteomics, Biomarker, Mass spectrometry, Pediatric

## Abstract

**Background:**

Rhabdomyosarcoma (RMS) is the most common soft tissue sarcoma with poor prognosis in children. The 5-year survival rate for early RMS has improved, whereas it remains unsatisfactory for advanced patients. Urine can rapidly reflect changes in the body and identify low-abundance proteins. Early screening of tumor markers through urine in RMS allows for earlier treatment, which is associated with better outcomes.

**Methods:**

RMS patients under 18 years old, including those newly diagnosed and after surgery, were enrolled. Urine samples were collected at the time points of admission and after four cycles of chemotherapy during follow-up. Then, a two-stage workflow was established. (1) In the discovery stage, differential proteins (DPs) were initially identified in 43 RMS patients and 12 healthy controls (HCs) using a data-independent acquisition method. (2) In the verification stage, DPs were further verified as biomarkers in 54 RMS patients and 25 HCs using parallel reaction monitoring analysis. Furthermore, a receiver operating characteristic (ROC) curve was used to construct the protein panels for the diagnosis of RMS. Gene Ontology (GO) and Ingenuity Pathway Analysis (IPA) software were used to perform bioinformatics analysis.

**Results:**

A total of 251 proteins were significantly altered in the discovery stage, most of which were enriched in the head, neck and urogenital tract, consistent with the most common sites of RMS. The most overrepresented biological processes from GO analysis included immunity, inflammation, tumor invasion and neuronal damage. Pathways engaging the identified proteins revealed 33 common pathways, including WNT/β-catenin signaling and PI3K/AKT signaling. Finally, 39 proteins were confirmed as urinary biomarkers for RMS, and a diagnostic panel composed of 5 candidate proteins (EPS8L2, SPARC, HLA-DRB1, ACAN, and CILP) was constructed for the early screening of RMS (AUC: 0.79, 95%CI = 0.66 ~ 0.92).

**Conclusions:**

These findings provide novel biomarkers in urine that are easy to translate into clinical diagnosis of RMS and illustrate the value of global and targeted urine proteomics to identify and qualify candidate biomarkers for noninvasive molecular diagnosis.

**Supplementary Information:**

The online version contains supplementary material available at 10.1186/s12014-023-09401-4.

## Introduction

Rhabdomyosarcoma (RMS) is the most common soft tissue sarcoma in childhood and accounts for approximately 54–70% of soft tissue sarcomas and 5–7% of all pediatric malignancies [1, 2]. RMS can present at any site, most commonly in the head and neck region, the genitourinary tract, limbs, retroperitoneum, and biliary tract [3]. The treatment for pediatric patients with RMS is based on a multimodality approach, including induction (multidrug) chemotherapy supplemented with surgery and/or radiotherapy. As reported by the Intergroup Rhabdomyosarcoma Study Group (IRSG), the five-year overall survival (OS) for pediatric patients with localized disease has improved with this multimodality approach. However, survival in patients with metastatic disease (26%-27% vs. 66%-85%) remains unsatisfactory [4]. This indicates that early diagnoses are needed to improve survival in pediatric patients with RMS.

Diagnosis of RMS is currently mainly dependent on pathological and imaging monitoring. However, using ultrasonography approximately, 15% of patients with RMS show distant metastases [5]. CT and MRI can only assess treatment efficacy based on tumor volume. PET-CT, with high radiation doses, has limitations in detecting lesions with a diameter < 5 mm [6]. Although lactate dehydrogenase (LDH) is significantly elevated in RMS, it can only indicate tumor burden status with a lack of specificity [7]. Thus, identification of more early, sensitive, and noninvasive surrogate markers of diagnosis and response is crucial.

Alterations in human proteins have been well recognized as indicators of pathophysiological changes caused by various diseases, including RMS. Several studies have revealed that GLI1, Gab1, GEFT, and FANCD2 are potential biomarkers and therapeutic targets in RMS in tissue specimens by immunohistochemical or western blot analysis [8–11]. Another study demonstrated that the IGFBP2 protein detected by ELISA in blood specimens identifies metastatic patients with worse event-free survival (EFS)[12]. Unlike traditional approaches to protein detection, only two studies have used proteomics to reveal multiple specific proteins and common pathways that may serve as biomarkers for RMS [13, 14]. However, they were carried out in vitro or in vivo rather than at the population level, resulting in limitations in their application in the clinic.

Urine is a good biological sample for biomarker analysis. Compared with plasma, it can quickly reflect changes in the body and identify low-abundance proteins [15, 16]. However, little is known about the specific protein characteristics and potential mechanism of RMS from urine samples, which is more meaningful for clinical application. Thus, the main purpose of this study was to identify and validate urinary biomarkers associated with tumor burden status that may be used for diagnosis and therapeutic monitoring of RMS in pediatrics using quantitative proteomics. These biomarkers may play critical roles in RMS diagnosis and improve understanding of the major pathophysiological pathways of RMS.

## Materials and methods

### Ethics and human subjects

All work performed in this study was approved by the Beijing Children's Hospital (BCH) Ethics Committee, and written informed consent was obtained from participants (2019–24). Pathological diagnosis of RMS was determined by two senior pathologists from different hospitals according to the authoritative guidelines in China [17]. Healthy controls (HCs) were recruited from children volunteers of employees at BCH.

### Experimental design

**Fig. 1:**
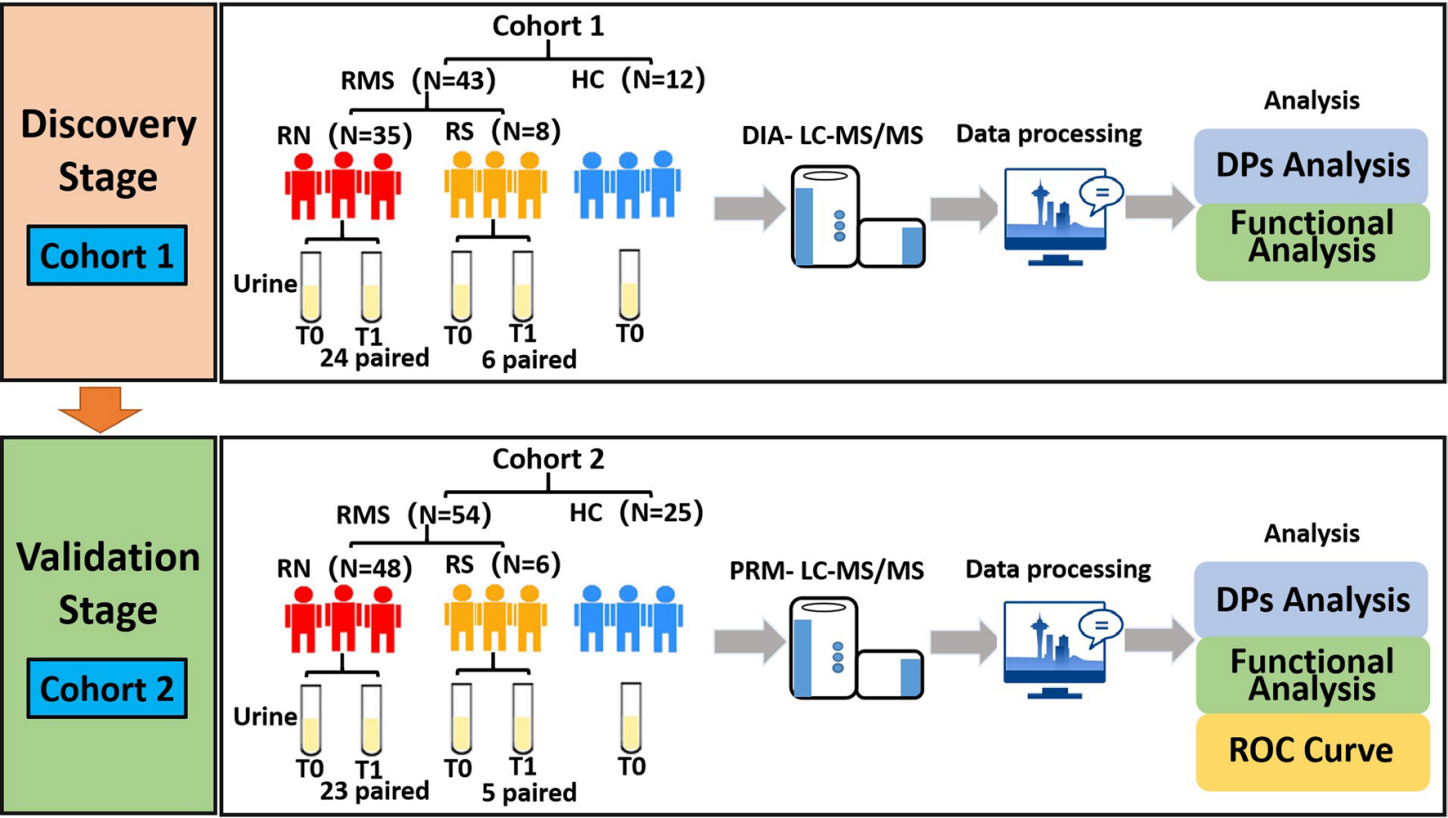
Study design of the two-stage workflow. *RMS* rhabdomyosarcoma, *HC* healthy control, *RN* newly diagnosed RMS patients at the time points of admission, *RS* RMS patients underwent surgery at the time points of admission, *T0* Timepoint of the first day of admission, *T1* Timepoint after 4 cycles of chemotherapy, *DIA* data-independent acquisition, *PRM* parallel reaction monitoring analysis, *DP* differential proteins, *ROC* receiver operating characteristic curve

The objective of the present study was to systemically identify and validate potential noninvasive diagnostic markers for RMS in urine. Therefore, three groups were recruited: newly diagnosed RMS patients (RN), RMS patients after surgery (RS), and HCs. For the RMS patients, urine samples were collected at admission (T0) and after four cycles of chemotherapy at follow-up (T1) (Fig. 1). Random morning midstream urine samples were collected from the HCs. All urine samples were immediately centrifuged at 3000 × g for 30 min at 4 °C to remove cell debris and then stored at − 80 °C.

A two-stage workflow was established as follows. (1) In the discovery stage, cohort 1 (43 RMS patients and 12 HC) was used to initially identify differential proteins (DPs) and explore the possible biological processes and disease pathways using data-dependent acquisition (DDA) and data-independent acquisition (DIA) quantitative proteomics strategy. (2) In the verification stage, cohort 2 (54 RMS patients and 25 HC) was used to verify the DPs as biomarkers and construct protein panels for clinical diagnosis of RMS by the parallel reaction monitoring (PRM) method (Fig. 1).

We divided the whole population into three groups. Group 1 included RMS patients receiving chemotherapy only (RN0 vs. RN1 vs. HC). Group 2 included RMS patients who underwent surgical treatment only (RN0 vs. RS0 vs. HC). Group 3 mainly included RMS patients who underwent both surgery and chemotherapy (RN0 vs. RS0 vs. RS1 vs. HC). In each group, comparisons were made before and after specific treatments, as well as between the RMS and HC groups. Only the DPs present in at least two of the above three groups were further selected.

### Sample preparation

The urine samples were first centrifuged at 5,000 × g for 30 min at 4 ◦C. The 15 mL of urine from each sample was precipitated with the same volume of acetone at − 20 ℃ overnight followed by centrifugation at 14,000 × g for 30 min at 4 ℃ to retain the precipitate and remove acetone by evaporation. The pellets were resuspended in a lysis buffer. The protein amount of each sample was quantified by the Bradford assay.

A total of 100 μg of protein was prepared using filter-aided sample preparation (FASP) for tryptic digestion (Trypsin Gold, Mass Spec Grade, Promega, Fitchburg, WI, USA) methods [18]. The protein samples were reduced with 20 mmol/L dithiothreitols at 37 ℃ for 5 min and alkylated with 50 mmol/L iodoacetamide (IAA, Sigma) for 45 min in the dark. After being centrifuged at 12000 g for 10 min, the supernatants were loaded into a 30-kDa filter device (Pall, Port Washington, NY, USA). The samples were then washed with 20 mM Tris 3 times and digested with trypsin (enzyme-to-protein ratio of 1:50) at 37 ℃ for 16–24 h. The peptide concentration was determined by the BCA method.

### LC–MS/MS analysis

The 10 ug digested peptides were dissolved in buffer A (deionized H_2_O, pH 10.0). Then, they were loaded onto an equilibrated fractionation C18 column (4.6 mm*250 mm, 3 μm) and eluted by buffer B (0.1% formic acid, 89.9% acetonitrile, deionized H_2_O) with separation gradient 5%-90%, flow rate 0.7 mL/min for 1 h. One tube component was collected every 1 min, and a total of 60 tube components were collected and combined into 20 fractions. They were vacuumized and stored at -20℃ for building the database.

All samples were analyzed with Orbitrap Fusion Lumos Tribrid Mass Spectrometer and EASY-nLC 1000 liquid chromatography (Thermo Fisher Scientific, Waltham, MA, USA). The 20 fractions obtained from the column separation were analyzed with mass spectrometry in DDA mode for the generation of the spectral library. Then 10 μL of the peptide from each fraction was loaded into a reversed-phase analytical column (75 μm * 100 mm, 3 μm, C18) at a flow rate of 0.3 μL/min and eluted with a gradient of 5–30% buffer B (0.1% formic acid in 99.9% acetonitrile) for 90 min. The MS data were acquired in high-sensitivity mode with the following parameters: full MS scan acquired within a 350–1500 m/z range with the resolution set to 60,000 for 3 s, MS/MS scan acquired in Orbitrap mode with a resolution of 15,000, 32% HCD collision energy.

The individual sample was analyzed with mass spectrometry in DIA-MS mode. The 20 ug peptides of each sample were dissolved in 20 μL 0.1% formic acid and 3μL of each peptide sample was mixed to form the quality control (QC) sample. Then the 10μL of each sample was first separated by chromatography and the liquid parameters settings of DIA were the same in DDA mode. The MS data were acquired in high-sensitivity mode with the following parameters: full MS scan acquired within a 350–1300 m/z range with the resolution set to 120,000 for 3 s, MS/MS scan acquired in Orbitrap mode with a resolution of 30,000, 32% HCD collision energy. A single DIA analysis of pooled QC peptides was performed after every 8 to 10 samples to monitor the stability of mass spectrometry.

We analyzed all the samples in random orders to avoid system errors, and different groups of samples were interleaved and analyzed. In addition, we assured the same person to handle the protein digestion procedure of all urinary samples used for both DIA and PRM analysis.

### MS data analysis

To generate a spectral library, DDA raw files were first searched by Proteome Discoverer (version 2.4; Thermo Scientific) with SEQUEST HT against the Swiss-Prot human database. Minimum and maximum peptide lengths were set to 6 and 144, respectively. Parent ion mass tolerances were set to 10 ppm, and fragment ion mass tolerance was set to 0.02 Da. Dynamic modification was oxidation/ + 15.995 Da (M), and the static modification was carbamdomethyl/ + 57.021 Da (C). The false-positive rate (FDR) cut-off was set at 1% at the protein level. The numbers of peptide-spectrum matches (PSMs), peptides, proteins, and protein groups were 109687, 18106, 3816 and 3199, respectively.

The search results were then imported into Spectronaut Pulsar (Biognosys AG, Switzerland) software to generate the spectral library. Minimum and maximum peptide lengths were set to 6 and 52, respectively. Confidence levels of peptides and proteins were set to high, which were selected as the proteome discoverer score type. The FDR was set to 1% at the protein level. Six fragment ions were set to calculate the peptide intensity, and at least one peptide was set to calculate the protein intensity. The numbers of precursors, peptides, proteins, and protein groups in the library were 22941, 17845, 3356 and 3243, respectively.

QC and individual acquisition DIA files were imported into Spectronaut Pulsar with default settings. The peptide retention time was calibrated according to iRT data. The systematic variance of the LC‒MS performance was calibrated using cross-run normalization and local normalization based on local regression. The IDPicker algorithm was used to perform protein inference. Peak areas of the respective fragment ions for MS2 were summed to calculate the peptide intensity, and the respective peptide intensity was summed to calculate the protein intensity. The 1% FDR at the protein level was used as a filter. Each protein contained at least 1 unique peptide.

### Parallel reaction monitoring analysis

Selected proteins in the 108 samples were verified by the parallel reaction monitoring (PRM) method, and each sample was analyzed by schedule mode. Analysis of the mixed sample was used as QC to observe the stability of the instrument signal during the whole analysis process among every 8 to 10 samples. iRT standard peptide analysis was added to each sample to observe the stability of the chromatographic retention time. To reduce system bias, different groups of samples were analyzed in a random order for mass spectrometry analysis.

Each sample was analyzed with a C18 RP self-packed capillary LC column (75 μm × 500 mm) and an eluted gradient of 5–30% buffer B2 (0.08% formic acid and 80% ACN; flow rate: 1.5 μL/min) for 25 min. An Orbitrap Exploris 480 Mass Spectrometer was used to analyse the peptides eluted by liquid chromatography. MS data were acquired using the high-sensitivity mode with the following parameters: PRM mode, full scans acquired at a resolution of 60,000 and MS/MS scans at a resolution of 15,000, rolling collision energy, charge state screening (including precursors with + 2 to + 4 charge state), MS/MS scan range of 350–1200 m/z, and maximum injection time of 20 ms.

Skyline 3.6 software was used to process PRM data, and correct peaks of all peptides were selected manually and exported. Progenesis software was used to extract the total ionic chromatography (TIC) of the + 2- + 5 charges of each sample. The TIC was used to normalize the abundance of each peptide and correct the sample loading amount and MS signal intensity. Six fragment ions were set to calculate the peptide intensity. The PRM results were exported for further analysis.

### Statistical analysis

Missing values of proteomic data in the samples were imputed with the sequential KNN method in different subgroups independently. Student’s t tests and one-way ANOVA were used for statistical analyses of quantitative data. Statistical significance was defined as FC > 1.5 or < 0.67 and a two-sided P value of less than 0.05. Orthogonal projection to latent structures discriminant analysis (OPLS-DA) was performed using SIMCA 14.0 (Umetrics, Sweden) software based on the selected biomarkers.

In the process of panel construction, a receiver operating characteristic (ROC) curve was used to evaluate the external efficacy and performed by the biomarker analysis module of the MetaboAnalyst 5.0 platform (https://www.metaboanalyst.ca/). As the data type was peak intensities, it was transformed by log transformation (base 10) and normalized by the median. Logistic regression and tenfold cross-validation were iteratively conducted for model training, parameter optimization, and performance evaluation. The 95% confidence interval (CI) was calculated using 100 bootstraps. The areas under the curve (AUCs) were calculated for each verified protein. The proteins with the top ten AUC values were randomly selected to form the panel and improve diagnostic efficiency. The criteria for the selection of the proteins for the panel were as follows: (1) the panel contained no more than 5 constituent proteins; (2) the panel with the largest AUC was selected.

### Bioinformatics analysis

DPs were analyzed by Gene Ontology (GO) based on biological processes, cellular components, and molecular functions using DAVID. Biological pathway analysis and disease/biofunction analysis were performed by Ingenuity Pathway Analysis (IPA) software (Ingenuity Systems, Mountain View, CA, USA). The Human Protein Atlas database (http://www.proteinatlas.org/) was used to conduct tissue distribution analysis. Figures were visualized in GraphPad Prism software (v9; San Diego, CA, USA) and the Wu Kong platform (https://www.omicsolution.org/wkomics/main/).

## Results

### Clinical characteristics of subjects

**Fig. 2:**
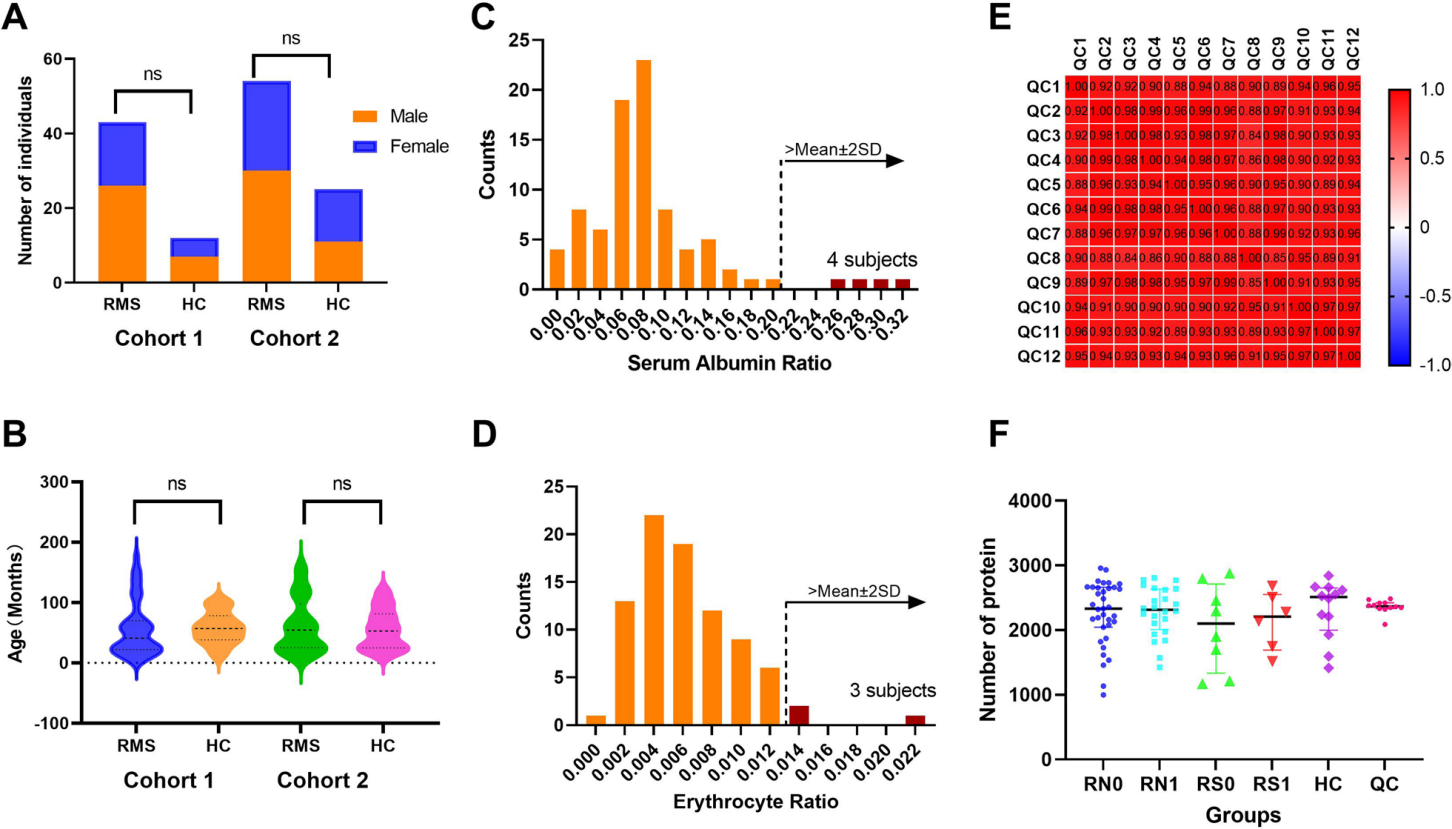
Clinical characteristics of subjects and urine proteome changes. **A** Gender distribution of cohort 1 and cohort 2. **B** Age distribution of cohort 1 and cohort 2. **C**Analysis of albumin proportion to total proteins in urine samples. **D** Analysis of urine samples contaminated with erythrocytes. **E** The correlation analysis of 12 QC samples by Pearson correlation coefficient. **F** The number of identified proteins in RN0, RN1, RS0, RS1, HC, and QC groups. RMS: rhabdomyosarcoma. *HC* healthy control, *QC* quality control samples, *RN0* newly diagnosed RMS at the time points of admission, *RN1* newly diagnosed RMS at the time points after 4 cycles of chemotherapy, *RS0* RMS underwent surgery at the time points of admission, *RS1* RMS underwent surgery at the time points after 4 cycles of chemotherapy

In the discovery stage, a total of 55 subjects, including 35 newly diagnosed patients (RN0), 8 RMS patients treated with surgery only (RS0), and 12 HCs, were initially recruited for cohort 1 (Fig. 1). There were 26 males and 17 females with a median age of 41.00 (22.00–70.00) months among the RMS patients. Of these patients, 24 RT0 patients (RN1) and 6 RS0 patients (RS1) had completed 4 cycles of chemotherapy at follow-up. Furthermore, 12 healthy subjects, including 7 males and 5 females with a median age of 57.00 (38.25–78.25) months, were recruited for comparison. There was no significant difference between the RMS patients and the HCs in terms of age (P = 0.89) or gender (P = 0.89) in cohort 1 (Table 1; Fig. 2A, B).

**Table 1:** Characteristics of RMS and HC group in cohort 1 and cohort 2

Variables	Cohort 1 (N = 55)			Cohort 2 (N = 79)		
	RMS (N = 43)	HC (N = 12)	*P*	RMS (N = 54)	HC (N = 25)	*P*
Age (Months)	41.00(22.00–70.00)	57.00(38.25–78.25)	0.89	54.5(25.38–98.00)	53.00(25.00–81.00)	0.62
Gender (M/F)						
Male	26(60.47)	7(58.33)	0.89	30(55.56)	11(44.00)	0.34
Female	17(59.53)	5(41.67)	–	24(44.44)	14(56.00)	–
LDH (U/L)	307.00(261.5–394.00)	–	–	306.50(246.50–373.75)	–	–
Pathological type						
ARMS	18(41.86)	–	–	28(51.85)	–	–
ERMS	25(58.14)	–	–	25(46.30)	–	–
Sites of the primary tumor						
Head and neck	13(30.23)	–	–	17(31.48)	–	–
Eye socket	1(2.33)	–	–	2(3.70)	–	–
Beside the meninges area	12(27.91)	–	–	14(25.93)	–	–
Limbs	4(9.30)	–	–	17(31.48)	–	–
Trunk	3(6.98)	–	–	6(11.11)	–	–
Abdominal pelvic	6(13.95)	–	–	6(11.11)	–	–
Genitourinary system	4(9.30)	–	–	2(3.70)	–	–
Tumor diameter						
≤ 5 cm	24(43.56)	–	–	32(59.26)	–	–
5-10 cm	15(34.88)	–	–	17(31.48)	–	–
≥ 10 cm	4(9.30)	–	–	5(9.26)	–	–
FOXO1						
Negative	26(60.47)	–	–	26(48.15)	–	–
Positive	15(34.88)	–	–	22(40.74)	–	–
Risk assessment						
Low-risk	4(9.30)	–	–	2(3.70)	–	–
Moderate-risk	33(76.74)	–	–	45(83.33)	–	–
High-risk	3(6.98)	–	–	3(5.56)	–	–
Central aggression	3(6.98)	–	–	4(7.41)	–	–

*RMS* rhabdomyosarcoma, *HC* healthy control, *LDH* lactate dehydrogenase, *ARMS* alveolar rhabdomyosarcoma, *ERMS* embryonal rhabdomyosarcoma

In the verification stage, cohort 2 was used to verify DPs. Cohort 2 included 34 RNs, 5 RSs, and 11 HCs from cohort 1 plus an additional 14 RNs and 14 HCs. The RMS patients included 30 males and 24 females with a median age of 54.50 (25.38–98.00) months. There were 23 RT0 patients (RN1) and 5 RS0 patients (RS1) who completed 4 cycles of chemotherapy. The healthy subjects included 11 males and 14 females with a median age of 53.00 (25.00–81.00) months. There was no significant difference between the RMS patients and the HCs in terms of age (P = 0.62) or gender (P = 0.34) in cohort 2 (Figs. 2A, B).

### Quality assessment and detection of urinary proteome alterations

We first analyzed the urine proteome of cohort 1 using label-free DIA-LC‒MS/MS quantitation. The method of Mann et al. [19] was used to remove samples contaminated by albumin or erythrocytes. Samples with fewer than 1000 identified proteins and those contaminated with albumin or erythrocytes, including 1 HC subject and 2 RMS cases, were further excluded (Fig. 2C, 2D). Correlation analysis showed that the mean correlation coefficient of the 12 QC samples was 0.93, which indicated the strong stability of mass spectrometry in this research (Fig. 2E). The sequential-KNN method was used to impute proteomic data in 5 groups (RN0, RN1, RS0, RS1, and HC) separately and remove missing proteins in more than 50% of the samples.

**Fig. 3:**
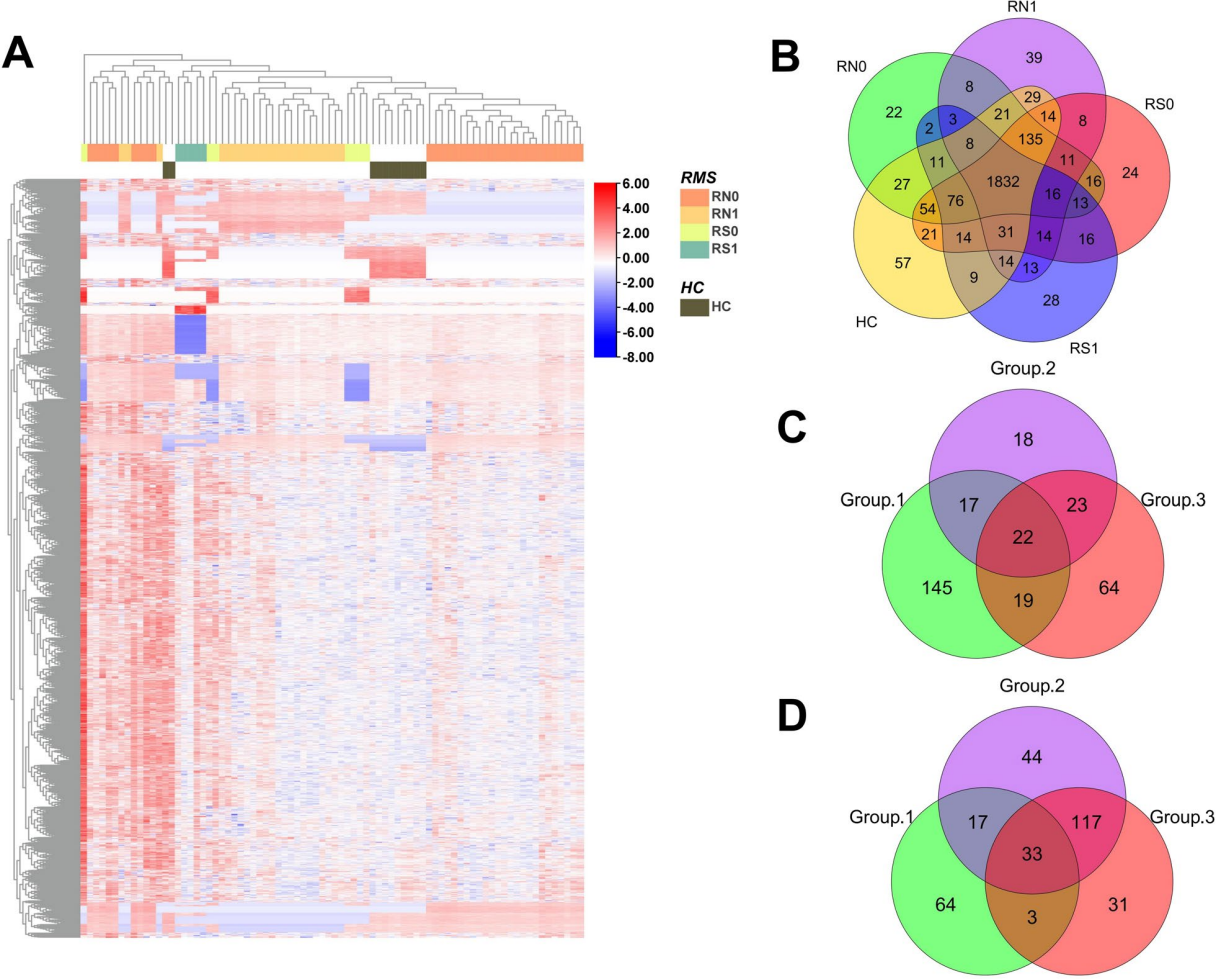
The global proteomic profiles using DIA LC–MS/MS quantitative proteomics strategy in cohort 1. **A** The heatmap of global proteomic profiles and the clustering results of the samples from five different groups. **B** Venn diagram of proteome distribution between five groups. **C** Up-regulated differential proteins between Groups 1, 2, and 3. **D** Down-regulated differential proteins between Groups 1, 2, and 3. RN0: newly diagnosed RMS at the time points of admission. *RN1* newly diagnosed RMS at the time points after 4 cycles of chemotherapy, *RS0* RMS underwent surgery at the time points of admission, *RS1* RMS underwent surgery at the time points after 4 cycles of chemotherapy, *HC* healthy control

**Fig. 4:**
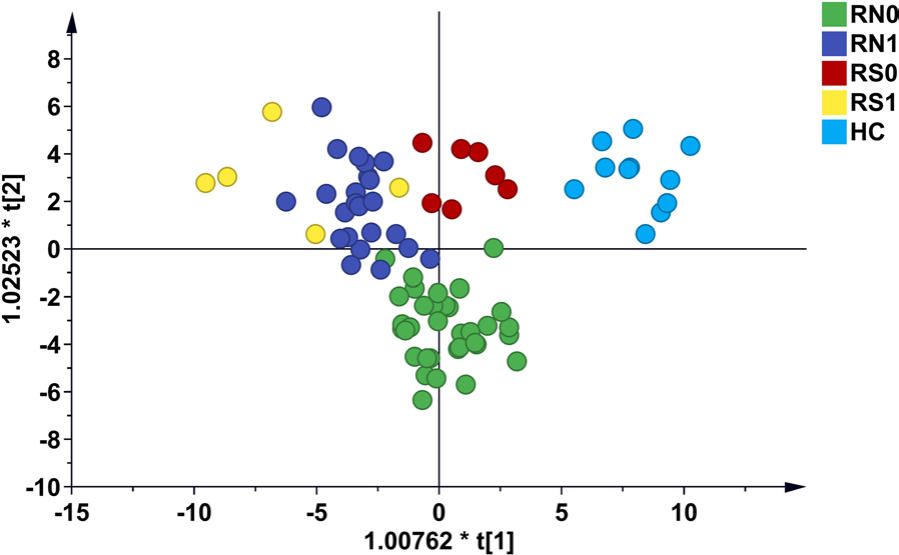
Pattern recognition analysis (OPLS-DA) of 251 differential proteins among RN0, RN1, RS0, RS1, and HC identified by DIA strategy. *OPLS-DA* orthogonal projection to latent structures discriminant analysis, *RN0* newly diagnosed RMS at the time points of admission, *RN1* newly diagnosed RMS at the time points after 4 cycles of chemotherapy, *RS0* RMS underwent surgery at the time points of admission, *RS1* RMS underwent surgery at the time points after 4 cycles of chemotherapy, *HC* healthy control

A total of 2586 proteins were finally identified (specific peptides ≥ 2 and FDR < 1%). The protein numbers of RN0, RN1, RS0, RS1, and HC were 2255, 2196, 2295, 2100, and 2353, respectively (Fig. 2F). The heatmap of global proteomic profiles showed that most RN0 patients were clustered in the same group (Fig. 3). The same trend was seen in HC groups. Among them, there were a total of 1832 intersecting proteins. According to the analysis strategy in the study design, 320, 291, and 312 DPs were identified in Groups 1, 2, and 3, respectively (Fig. 3A). Finally, a total of 251 DPs were identified under the indicated criteria, including 81 up-regulated and 170 down-regulated proteins (Fig. 3B, C, Additional file 1: Table S1). Pattern recognition analysis of OPLS-DA was performed, and the results indicated that RMS could be distinguished from HC based on urinary proteins (Fig. 4).

### Annotation and functional analysis

**Fig. 5:**
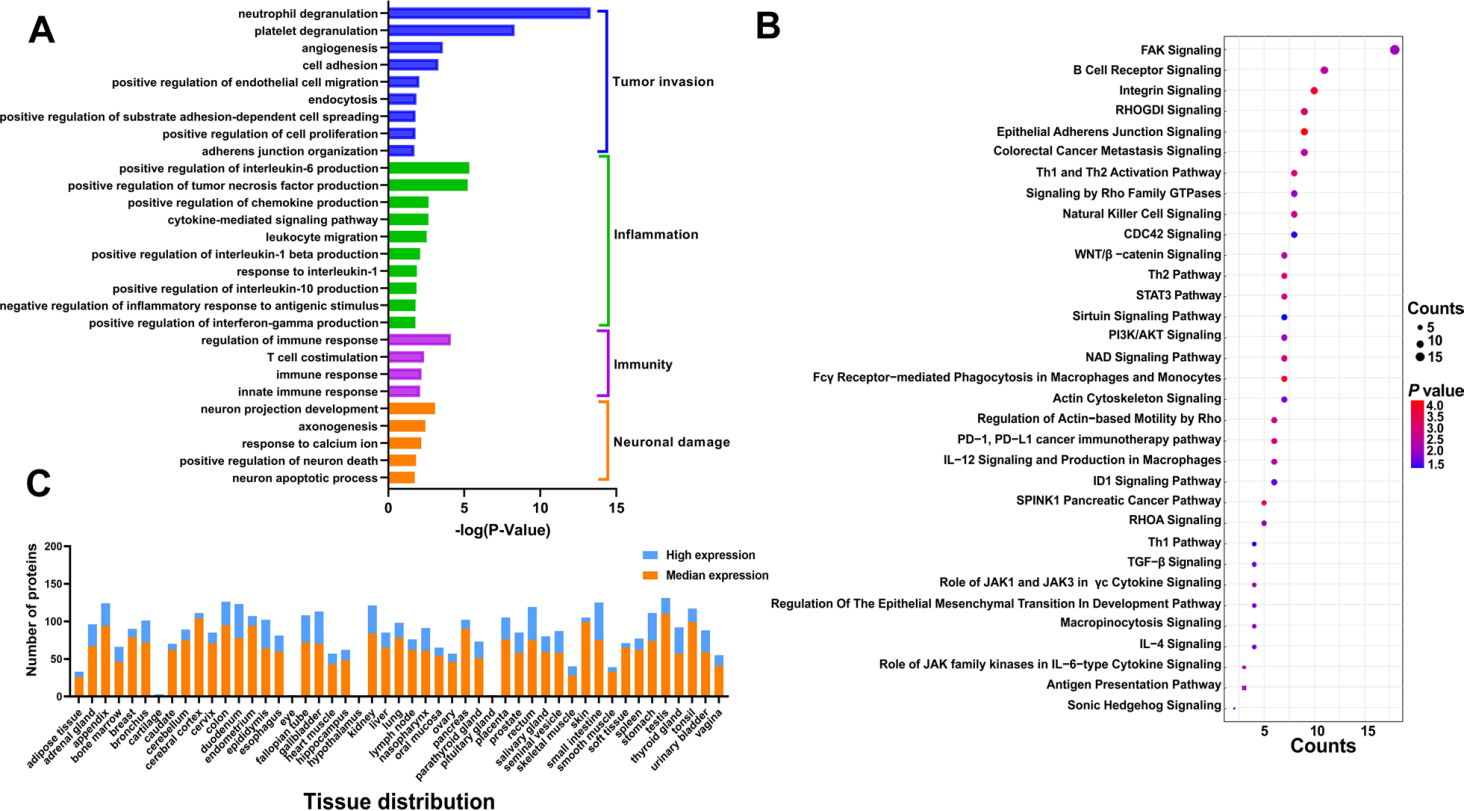
Annotation and functional characterization of differential proteins. **A** Biological process analysis of differential proteins. **B** Bubble diagram of pathway analysis between differential proteins. **C** The distribution of the tissue-enriched proteins

The pathogenesis and biological mechanism of RMS were associated with four kinds of biological processes, including immunity, inflammation, tumor invasion, and neuronal damage (Fig. 5A, Additional file 2: Table S2). In terms of tumor invasion, RMS was mainly related to neutrophil degranulation, angiogenesis, and cell adhesion. Additionally, RMS is related to positive regulation of interleukin-6, interleukin-1, interleukin-10 tumor necrosis factor, and interferon-γ production in inflammation. Regarding immunity, RMS was more relevant to regulation of the immune response and T-cell costimulation. Interestingly, we noticed that RMS also had a close relationship with neuronal damage, which is consistent with the clinical characteristics of perineural invasion in RMS. The main biological processes included neuron projection development, axonogenesis, response to calcium ions, positive regulation of neuron death, and neuron apoptotic process.

In pathway analysis by IPA, the DPs were mostly involved in regulation of WNT/β-catenin signaling, PI3K/AKT signaling, and the FAT10 cancer signaling pathway, which modulate the process of cell differentiation and proliferation (Fig. 5B, Additional file 3: Table S3). Disease and function analysis showed 43 DPs to be associated with soft tissue sarcomas. Among them, 26 and 39 DPs are reported to be connected with an extracranial or malignant genitourinary solid tumor, respectively. In addition, 25 DPs are related to muscle tumors; 8 DPs (APOE, FCER1A, FCER2, GSK3B, HSPD1, IL6R, MMP7, and RARRES2) are linked to proliferation of muscle cells (Additional file 4: Table S4).

### Tissue distribution analysis

Expression levels of protein biomarkers in different tissues are very useful for speculation of target organ damage. The Tissue Atlas contains information regarding expression data for human genes from 44 normal human tissue types at both mRNA and protein levels. As urine biomarkers can reflect changes in organs in the early stage, the organ origin of the 251 DPs was searched from The Tissue Atlas, and their expression-enriched or enhanced tissue types were acquired. In total, 191 of the 251 proteins are distributed in 41 solid tissues as tissue-enriched proteins (Fig. 5C, Additional file 5: Table S5). The above DPs are mostly distributed in the testis, colon, cerebral cortex, prostate, and so on, consistent with the most common sites of RMS. Additionally, forty DPs are reported to be highly or moderately expressed in skeletal muscle. Because RMS is a malignant neoplasm characterized by early myogenic differentiation, which implicates a skeletal myoblastic cell of origin, these proteins enriched in skeletal muscle may provide more clues regarding the pathogenesis of RMS.

### Validation of the urinary protein biomarkers

**Fig. 6:**
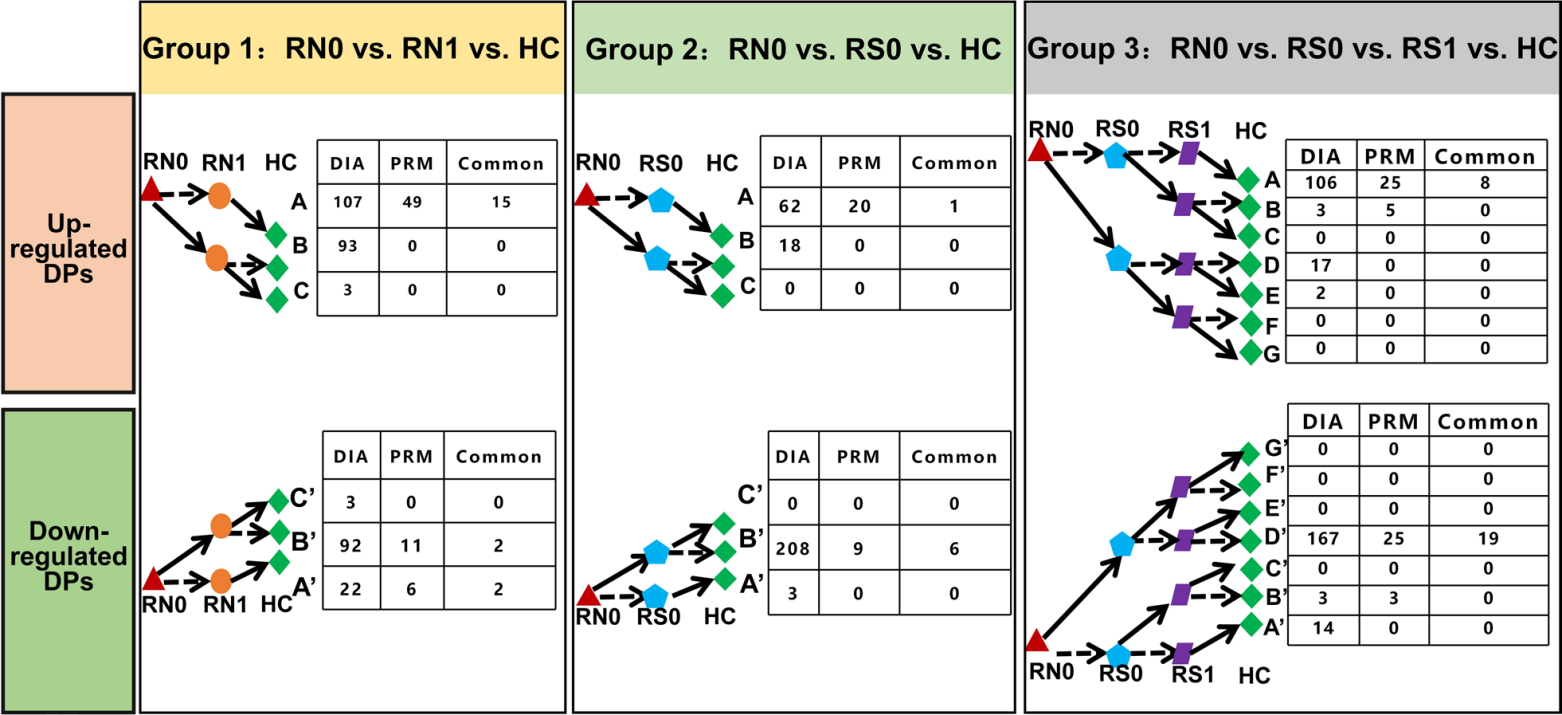
Comparison of the effects of pre-and post-chemotherapy (Group 1: RN0 vs RN1 vs HC), pre-and post-surgery (Group 2: RN0 vs RS0 vs HC), and surgery plus chemotherapy (Group 3: RN0 vs RS0 vs RS1 vs HC) on the distribution of urine protein identified by DIA and PRM. RMS: rhabdomyosarcoma. *HC* healthy control, *RN0* newly diagnosed RMS at the time points of admission, *RN1* newly diagnosed RMS at the time points after 4 cycles of chemotherapy, *RS0* RMS underwent surgery at the time points of admission, *RS1* RMS underwent surgery at the time points after 4 cycles of chemotherapy, *DIA* data-independent acquisition, *PRM* parallel reaction monitoring analysis, *DP* differential proteins

**Fig. 7:**
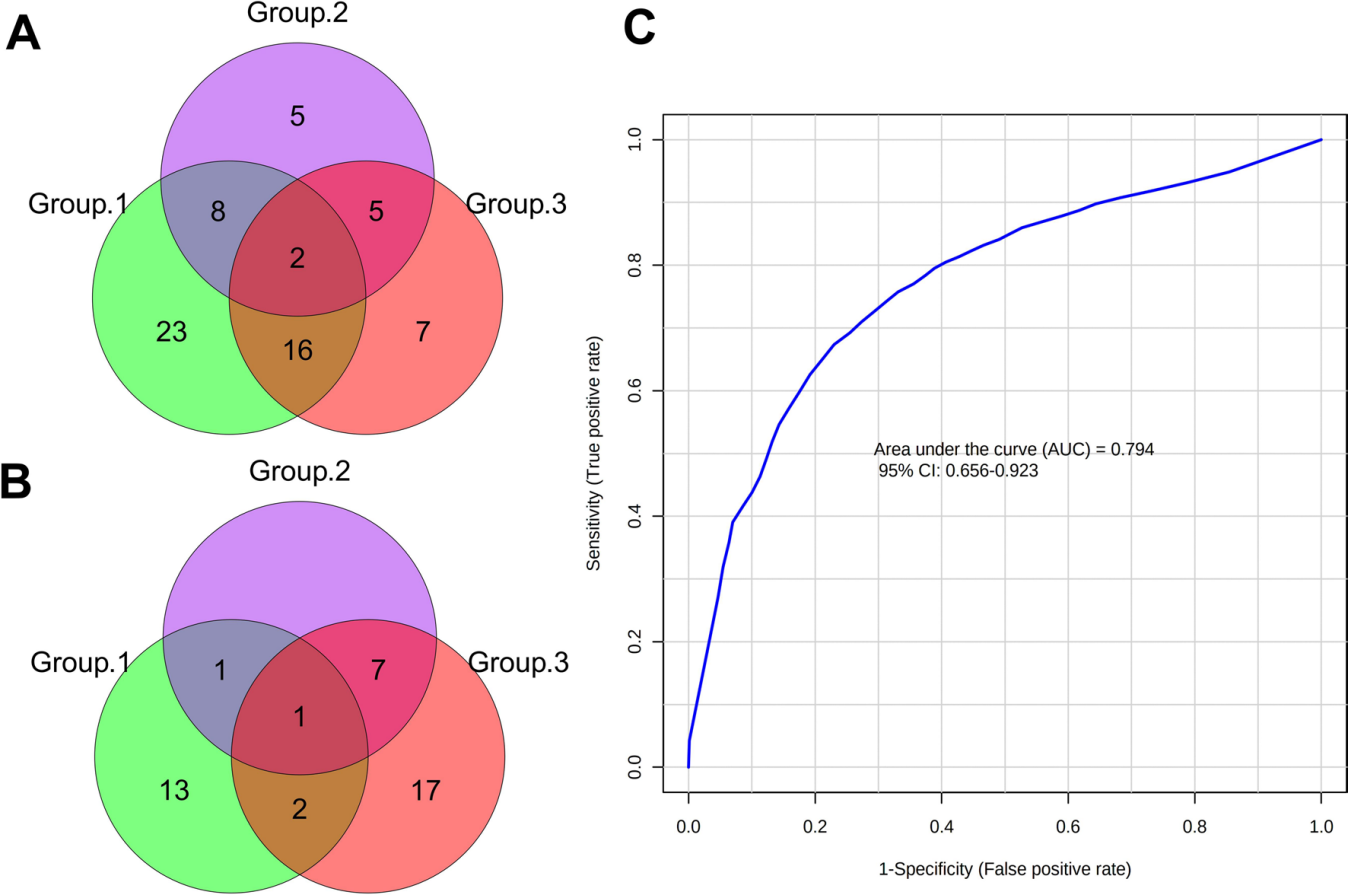
Proteome distribution and diagnostic panel construction for RMS and HC using PRM-based targeted proteomic method. **A** Up-regulated differential proteins between groups 1, 2, and 3 using PRM. **B** Down-regulated differential proteins between groups 1, 2, and 3 using PRM. **C** ROC curve of the diagnostic panel for RMS and HC from the tenfold cross-validation

We further analyzed the urine proteome of cohort 2 to validate all DPs found in cohort 1 using the PRM assay (Additional file 6: Table S6). Sixty-five, 29, and 54 DPs were identified in Groups 1, 2, and 3, respectively (Fig. 6). Referring to the DIA analysis process, thirty-nine urinary proteins were finally verified, including sixteen up-regulated and twenty-three down-regulated proteins (Fig. 7A, B, Table 2). Of note, six of the thirty-nine proteins (AXL, GINM1, SLC10A3, SLC39A14, SPINT1, and TMEM132A) are reported to be highly or moderately expressed in skeletal muscle.

**Table 2:** Literature analysis of thirty-nine validated urinary biomarkers

UniProt accession	Gene name	Protein name	Trend	Associated with RMS	Associated with muscular tissue	Reported as urine biomarkers	Reported as cancer biomarkers
P09237	MMP7	Matrilysin	Up	×	×	IgA, kidney fibrosis	Colon cancer, cervical cancer, gastric cancer, bladder cancer, ovarian cancer, oral verrucous and squamous cell cancer, head and neck squamous cell carcinomas
Q13938	CAPS	Calcyphosin	Up	×	×	×	Pediatric primitive neuroectodermal tumors, breast cancer, lung cancer
P15907	ST6GAL1	Beta-galactoside alpha-2,6-sialyltransferase 1	Up	×	√	×	Hepatocellular carcinoma, gastric cancer, colorectal cancer
P30086	PEBP1	Phosphatidylethanolamine-binding protein 1	Up	×	×	Prostate cancer, ovarian cancer, clear cell renal cell carcinoma	Bladder cancer, pancreatic cancer, lung adenocarcinoma, hepatocellular carcinoma
Q07075	ENPEP	Glutamyl aminopeptidase	Up	×	×	Familial Parkinson's disease	Colorectal cancer, breast cancer
Q24JP5	TMEM132A	Transmembrane protein 132A	Up	×	×	×	×
Q9H6S3	EPS8L2	Epidermal growth factor receptor kinase substrate 8-like protein 2	Up	×	×	Proliferative lupus nephritis, renal cell carcinoma	Endometrial carcinoma
P01911	HLA-DRB1	HLA class II histocompatibility antigen, DRB1 beta chain	Up	×	×	Renal allograft rejection	×
P01009	SERPINA1	Alpha-1-antitrypsin	Up	×	×	Preeclampsia, primary nephrotic syndrome, respiratory pathologies, bladder cancer, prostate cancer, morquio syndrome, hypertension	Bladder cancer, oral cancer, pancreatic cancer, non-small-cell lung cancer
P02671	FGA	Fibrinogen alpha chain	Up	×	×	Prostate cancer, bladder cancer, renal allograft rejection	Prostate cancer, bladder cancer
O14745	SLC9A3R1	Na( +)/H( +) exchange regulatory cofactor NHE-RF1	Up	×	×	×	Hepatocellular carcinoma
Q10588	BST1	ADP-ribosyl cyclase/cyclic ADP-ribose hydrolase 2	Up	×	×	Polycystic and other chronic kidney diseases, chronic lung allograft dysfunction	Prostate cancer
O75309	CDH16	Cadherin-16	Up	×	×	Sepsis-induced acute kidney injury	Papillary thyroid cancer, lung adenocarcinoma
P31997	CEACAM8	Carcinoembryonic antigen-related cell adhesion molecule 8	Up	×	×	×	Oral squamous cell carcinoma, non-small cell lung cancer, gastric cancer
Q9UKK9	NUDT5	ADP-sugar pyrophosphatase	Up	×	×	×	Breast cancer, colon cancer
Q9UN74	PCDHA4	Protocadherin alpha-4	Up	√	×	×	Cervical cancer
Q13705	ACVR2B	Activin receptor type-2B	Down	×	√	×	Colorectal cancer
Q6FHJ7	SFRP4	Secreted frizzled-related protein 4	Down	×	×	×	Endometrial carcinoma, bladder cancer, gastric cancer, colon cancer
O43278	SPINT1	Kunitz-type protease inhibitor 1	Down	×	×	Urothelial carcinoma	Prostate adenocarcinoma, cervical cancer,
							Endometrial carcinoma, CLL, pancreatic ductal adenocarcinoma
O43291	SPINT2	Kunitz-type protease inhibitor 2	Down	×	×	×	Cervical cancer, endometrial carcinoma, breast cancer, prostate cancer
P08174	CD55	Complement decay-accelerating factor	Down	×	×	Respiratory pathologies, prostate cancer	Colon cancer, primary gallbladder carcinoma, bladder cancer
P09131	SLC10A3	P3 protein	Down	×	×	×	×
P09486	SPARC	SPARC	Down	×	×	Clear cell renal cell carcinoma, urinary bladder cancer	Rectal cancer, non-small-cell lung cancer, pancreatic cancer, gastric cancer, hypopharyngeal cancer, colon cancer, diffuse large B-cell lymphoma, breast cancer
P13598	ICAM2	Intercellular adhesion molecule 2	Down	×	×	×	Lung cancer
P15151	PVR	Poliovirus receptor	Down	×	×	×	Multiple myeloma, bladder cancer
P16112	ACAN	Aggrecan core protein	Down	×	×	×	Gastric cancer, breast cancer
P19022	CDH2	Cadherin-2	Down	×	×	Respiratory pathologies, diabetic nephropathy	Malignant bone and soft tissue tumors, ductal carcinoma, glioblastoma, papillary thyroid carcinoma
P19652	ORM2	Alpha-1-acid glycoprotein 2	Down	×	×	Rheumatoid arthritis, adult-onset Still's disease	colorectal cancer, cervical cancer, cholangiocarcinoma, papillary thyroid carcinoma, liver cancer
P20138	CD33	Myeloid cell surface antigen CD33	Down	×	×	×	Colorectal cancer, AML
P30530	AXL	Tyrosine-protein kinase receptor UFO	Down	√	×	×	Osteosarcoma, melanoma, esophageal adenocarcinoma, non-small cell lung cancer, colorectal adenocarcinoma, Wilms' tumor
Q15043	SLC39A14	Metal cation symporter ZIP14	Down	×	×	×	Colorectal cancer, prostate cancer, breast cancer
Q9HBB8	CDHR5	Cadherin-related family member 5	Down	×	×	Autism	Hepatocellular carcinoma, clear cell renal cell carcinoma, pancreatic ductal adenocarcinoma
Q9NU53	GINM1	Glycoprotein integral membrane protein 1	Down	×	×	Bladder carcinoma	Bladder carcinoma
Q9ULK6	RNF150	RING finger protein 150	Down	×	×	×	×
Q9Y4C0	NRXN3	Neurexin-3	Down	×	×	×	Glioblastoma
O75339	CILP	Cartilage intermediate layer protein 1	Down	×	×	Cartilage matrix turnover	Bladder cancer
Q9H6X2	ANTXR1	Anthrax toxin receptor 1	Down	×	×	×	Angiosarcoma, gastric cancer, colorectal cancer, lung cancer
P43490	NAMPT	Nicotinamide phosphoribosyltransferase	Down	√	×	×	Glioblastoma, bladder cancer, breast cancer, prostate cancer, basal cell carcinomas, breast invasive ductal carcinoma
Q9BXN2	CLEC7A	C-type lectin domain family 7 member A	Down	×	×	×	Clear cell renal cell carcinoma, AML

Whether the potential diagnostic biomarkers of RMS found in this study have ever been reported as biomarkers for other diseases has been widely investigated (Table 2). Three of thirty-nine proteins (PCDHA4, AXL, and NAMPT) correlate with RMS and RMS-associated tumor behavior. The other two proteins (ST6GAL1 and ACVR2B) are involved in proliferation, differentiation, and signaling of skeletal muscle. In addition, all urinary proteins, except for four (TMEM132A, HLA-DRB1, SLC10A3, RNF150), have been reported as candidate biomarkers for thirty-five neoplastic diseases. Of note, seventeen of thirty-nine proteins are reported as urine biomarkers in different diseases, of which eight proteins (PEBP1, EPS8L2, SERPINA1, FGA, SPINT1, CD55, SPARC, and GINM1) are related to neoplastic disease in urine specimens (Additional file 7: Table S7).

### Construction of the urinary protein biomarker panel for diagnosis

Based on cohort 2, we identified a compact biomarker combination containing 5 proteins, including EPS8L2, SPARC, HLA-DRB1, ACAN, and CILP. The AUC values of this 5-protein combination to distinguish the RMS and HC groups were calculated as 0.79 (95% CI = 0.66 ~ 0.92) (Fig. 7C). Moreover, the top five AUC values of individual proteins between the RN0 group and HC group ranged from 0.67 to 0.73 (Additional file 8: Table S8).

## Discussion

RMS is one of the most common highly malignant soft tissue sarcomas in children. It is of high priority to identify indicators that can diagnose and monitor the development of the disease and understand its pathogenesis. For this purpose, we conducted proteomics to profile urine protein alterations and identified 251 significant DPs. The accuracy of these biomarkers to diagnose RMS was validated via proteomics of PRM technology. The 39 proteins and a 5-protein panel were used as biomarkers to diagnose and monitor the development of RMS. Moreover, alterations in these DPs provide very valuable insight into the pathogenesis of RMS.

The DPs found in this study are mostly involved in regulation of WNT/β-catenin signalling, PI3K/AKT signalling, and FAT10 cancer signalling pathways, which modulate the process of cell differentiation and proliferation. Wnt signalling plays an important role in skeletal muscle differentiation. Recent studies suggest that canonical Wnt signalling is inactive in RMS [20]. Impaired Wnt signalling promotes the development of embryonal RMS with highly invasive properties in p53/c-fos double mutant mice [21]. Similarly, activating the canonical WNT/β-catenin pathway by GSK3 inhibition suppresses growth and self-renewal in embryonal RMS [22]. In addition, another study showed that common alterations in the RAS/MAPK and PI3K/AKT pathways exist in RMS, regardless of fusion status [23]. While these pathways are necessary for RMS cell proliferation and survival, it is not clear whether they contribute directly to RMS invasion, angiogenesis, or metastatic ability.

Additionally, consistent with prior reports, this study confirmed that the biological processes of RMS are associated with immunity, inflammation, and tumor invasion. Notably, neuronal damage was found to be related to the development of RMS in our study, in line with the clinical features of the disease. RMS most often occurs in the head and neck, given the ease of access to the invasion of the central nervous system by direct extension [24]. Nervous invasion has also been noted for RMS originating at sites outside the head and neck, suggesting hematogenous spread [25]. These results confirm that the altered urine proteins identified in this study indeed reflect authentic pathophysiological changes in response to RMS and minimize the possibility that the protein alterations were influenced by other factors.

We further validated thirty-nine urine proteins as possible novel biomarkers for RMS. Among them, three (NAMPT, PCDHA4, and AXL) have been reported to be associated with the pathogenesis of this tumor. Moiz Vora et al. reported that NAMPT is overexpressed in RMS compared to skeletal or smooth muscle tissue and that the level of NAMPT expression correlates with tumor behavior [26]. A recent study revealed that the DNA methylation patterns of PCDHA4 can distinguish between metastatic and nonmetastatic RMS and suggested a novel therapeutic target that may enhance the efficiency of RMS treatments [27]. Another study showed abundant tyrosine phosphorylation and expression of AXL in cell lines of ten sarcomas, including RMS samples. The authors showed that sarcoma cells and tissues express multiple tyrosine kinases, providing more clues to identify drivers of sarcoma growth and survival and limiting the effectiveness of targeted agents [28]. In addition, two other urinary proteins (ST6GAL1 and ACVR2B) found in this study are reported to be involved in the signalling regulation, proliferation, and differentiation of skeletal muscle [29, 30]. Moreover, seventeen of the biomarkers have been reported as urine biomarkers for more than twenty diseases. Among them, eight urinary proteins are related to different neoplastic diseases, mainly urothelial carcinoma, prostate cancer and bladder carcinoma. It is worth noting that this is consistent with the predisposition of RMS to occur in the genitourinary tract. Therefore, from the perspective of urine proteomics, our study found that these proteins can distinguish RMS patients from healthy children and that it can provide more important support for understanding the pathogenesis of RMS.

To extend the utility of our datasets, we used a logistic regression model to distinguish between RMS patients and HCs with high sensitivity and specificity. Importantly, given the performance of the model, EPS8L2, SPARC, HLA-DRB1, ACAN, and CILP are promising candidates as diagnostic markers to indicate early disease development of RMS. EPS8L2 was found to interact with Ctdnep1 to cause nuclear mispositioning by regulating dorsal actin cables for nuclear movement during cell migration, which is usually associated with cell dysfunction and disease, from muscular disorders to cancer metastasis [31]. One study validated EPS8L2 as an early, noninvasive urinary indicator for renal cell carcinoma (AUC: 0.81, 95%CI: 0.70–0.93) [32]. An additional report described that the peptides from EPS8L2 are associated with meningioma pathobiology through the integrin and PI3K-Akt pathways [33]. An association between EPS8L2 and several kinds of solid tumors was also found in our disease annotation analysis. Interestingly, EPS8L2 was closely related to biliary tract adenocarcinoma, which was consistent with the clinical characteristics of RMS. Another important indicator, SPARC, is reported to be one of the candidate biomarkers of chemotherapy efficacy for pediatric sarcoma, including RMS, Ewing sarcoma, and osteosarcoma [34]. Moreover, SPARC has been shown by several studies to be a key protein that attracts many kinds of cancer cells to the bone microenvironment and can be used as a prognostic indicator of tumor severity and/or aggressiveness [35–38]. In our study, SPARC was implicated in the biological process of angiogenesis and tumor invasion, migration, and shape change. Three other important proteins, HLA-DRB1, CHIP1, and ACAN, also play critical roles in the development and recurrence of benign and malignant diseases. Recent studies have highlighted HLA-DRB1 as a potential early marker for sarcomas that is significantly enriched in various immune-related logical processes and pathways [39, 40]. CHIP1, a matrix component of human articular cartilage, was found to be an important marker in RMS in this study. Similar reports have confirmed CHIP1 as a novel candidate indicator for the early detection of lung cancer and breast cancer brain metastasis in prognosis analysis [41, 42]. As the main component of the cell surface and extracellular matrix, ACAN can interact with a variety of key molecules, such as growth factors and cytokines, to participate in the epithelial-mesenchymal transition, thereby enhancing cell movement, migration, and invasion [43]. Several comparative proteomic studies have reported ACAN as a new serum tumor marker for hepatocellular or childhood acute lymphoblastic leukemia through regulation of the Hippo/YAP signalling pathway [44, 45]. Our study identified and validated the above proteins as urinary biomarkers in RMS, which may inform rational therapeutic targets by elucidating the immunologic, inflammatory and other cancer invasion mechanisms contributing to the aetiology of RMS.

There are some limitations in our study. First, some RMS patients and HCs in cohort 2 were also included in cohort 1. Although we applied two different approaches (i.e., DIA and PRM) to generate consistent results, it would be ideal to involve independent clinical samples from multiple centers to further validate the biomarkers we identified. Second, the relatively limited size of the sample might affect the results, though it takes longer to obtain sufficient samples of RMS because of the lower incidence. However, our study also found that individual heterogeneity was relatively obvious in the RMS group due to diverse tumor sites. Third, multiple testing corrections were not used in this study because strict testing correction may lead to false-negatives. More studies using larger samples are needed to verify the results in the future. Finally, the detailed roles of the indicator proteins in the pathogenesis of RMS require further investigation or experimental validation.

## Conclusions

This study profiled urine protein alterations and identified a series of valuable biomarker candidates, providing hints at the pathogenesis of RMS. These proteins show promising potential to be further developed as clinical biomarkers, either individually or in combination, to diagnose and closely monitor RMS development, thereby providing timely advice for clinical treatment. More importantly, this is the first study that reports a specific humoral response for RMS urine samples and suggests the value of urinary-based biomarkers in improving diagnostic capabilities for RMS patients.

## Supplementary Information

**Additional file 1: Table S1.** Distribution of 251 differential proteins in each comparison based on DIA data.

**Additional file 2: Table S2.** Biological processes analysis of 251 differential proteins.

**Additional file 3: Table S3.** Pathway analysis of 251 differential proteins.

**Additional file 4: Table S4.** Diseases annotation of 251 differential proteins.

**Additional file 5: Table S5.** Tissue distribution analysis of 251 differential proteins.

**Additional file 6: Table S6.** Distribution of differential proteins in each comparison based on PRM data.

**Additional file 7: Table S7.** Literature analysis of 39 validated urinary biomarkers.

**Additional file 8: Table S8**. Biomarkers panel for diagnosis of RMS.

## Data Availability

The datasets used and/or analyzed during the current study are available from the corresponding author on reasonable request.

## Abbreviations

RMS: Rhabdomyosarcoma
DP: Differential proteins
ROC: Receiver operating characteristic curve
OS: Overall survival
LDH: Lactate dehydrogenase
EFS: Event-free survival
HC: Healthy controls
DDA: Data-dependent acquisition
DIA: Data-independent acquisition
PRM: Parallel reaction monitoring
FASP: Filter-aided sample preparation
FDR: False positive rate
QC: Quality control sample
OPLS-DA: Orthogonal projection to latent structures discriminant analysis
CI: Confidence interval
