# Supplementary material for: Quantitative proteomics identifies and validates urinary biomarkers of rhabdomyosarcoma in children
Source: Clin Proteomics. 2023 Mar 14;20:10. doi: 10.1186/s12014-023-09401-4 (PMC10012572; doi:10.1186/s12014-023-09401-4)
Supplement: Supplementary file 1 — Additional file 1: Table S1. Distribution of 251 differential proteins in each comparison based on DIA data. [file 12014_2023_9401_MOESM1_ESM.pdf]

Table S1 Distribution of 251 differential proteins in each comparison based on DIA data.

UniProt accession	Protein Names	RN0	RN1	RS0	RS1	HC	RN0 vs RN1		RN1 vs HC		RN0 vs RS0		RS0 vs HC		RS0 vs RS1		RS1 vs HC	
							Fold Change	*P*	Fold Change	*P*	Fold Change	*P*	Fold Change	*P*	Fold Change	*P*	Fold Change	*P*
A0A075B6Q5	Immunoglobulin heavy variable 3-64	4.82E+04	4.28E+04	1.47E+05	7.42E+04	8.75E+04	1.13	0.94	0.49	0.22	0.33	0.03	1.67	0.31	1.98	0.19	0.85	0.56
A0A0B4J2D9	Immunoglobulin kappa variable 1D-13	7.02E+04	6.95E+04	6.96E+05	1.09E+05	3.08E+04	1.01	0.13	2.26	0.02	0.10	0.27	22.57	0.22	6.39	0.83	3.53	0.03
A0A0C4DH24	Immunoglobulin kappa variable 6-21	0.00E+00	1.84E+04	2.40E+05	0.00E+00	2.27E+04	—	—	0.81	0.19	—	—	10.57	0.18	—	—	—	—
O14520	Aquaporin-7	6.85E+04	3.10E+05	1.10E+06	9.06E+04	9.76E+04	0.22	0.25	3.18	0.66	0.06	0.04	11.28	0.23	12.15	0.54	0.93	0.63
O14618	Copper chaperone for superoxide dismutase	0.00E+00	1.56E+04	5.94E+04	0.00E+00	1.81E+04	—	—	0.86	0.56	—	—	3.29	0.66	—	—	—	—
O14745	Na(+)/H(+) exchange regulatory cofactor NHE-RF1	8.65E+04	7.45E+04	7.72E+04	4.91E+04	2.96E+04	1.16	0.41	2.52	0.04	1.12	0.97	2.61	0.01	1.57	0.24	1.66	0.70
O14786	Neuropilin-1	3.40E+04	2.07E+04	1.64E+04	4.53E+04	3.33E+04	1.65	0.01	0.62	0.37	2.07	0.01	0.49	0.17	0.36	0.07	1.36	0.32
O15144	Actin-related protein 2/3 complex subunit 2	2.69E+04	3.14E+04	3.18E+05	5.11E+04	3.04E+04	0.86	0.57	1.03	0.89	0.08	0.01	10.48	0.13	6.23	0.61	1.68	0.24
O15145	Actin-related protein 2/3 complex subunit 3	0.00E+00	1.16E+04	1.14E+05	7.14E+03	9.97E+03	—	—	1.17	0.57	—	—	11.44	0.06	15.97	0.12	0.72	0.90
O43242	26S proteasome non-ATPase regulatory subunit 3	0.00E+00	1.46E+04	1.45E+05	0.00E+00	1.12E+04	—	—	1.30	0.56	—	—	12.89	0.57	—	—	—	—
O43278	Kunitz-type protease inhibitor 1	7.45E+04	1.05E+05	2.22E+05	1.63E+05	7.80E+04	0.71	0.01	1.35	0.08	0.34	0.01	2.85	0.12	1.36	0.91	2.10	0.10
O43291	Kunitz-type protease inhibitor 2	9.31E+04	1.82E+05	8.31E+05	7.68E+04	1.84E+05	0.51	0.36	0.99	0.87	0.11	0.03	4.52	0.30	10.81	0.35	0.42	0.88
O43633	Charged multivesicular body protein 2a	5.99E+04	6.34E+04	3.27E+05	3.90E+04	9.00E+04	0.95	0.62	0.70	0.53	0.18	0.03	3.64	0.20	8.39	0.19	0.43	0.72

O43707	Alpha-actinin-4	6.51E+04	3.86E+04	1.63E+05	3.06E+04	8.78E+04	1.69	0.42	0.44	0.51	0.40	0.13	1.86	0.04	5.33	0.01	0.35	0.38
O43795	Unconventional myosin-Ib	2.13E+04	2.49E+04	7.90E+04	2.17E+04	1.70E+04	0.86	0.89	1.47	0.36	0.27	0.05	4.65	0.05	3.64	0.30	1.28	0.57
O43865	S-adenosylhomocysteine hydrolase-like protein 1	2.69E+04	2.19E+04	1.60E+05	2.41E+04	3.80E+04	1.23	0.47	0.58	0.51	0.17	0.04	4.20	0.35	6.62	0.36	0.63	0.76
O60241	Adhesion G protein-coupled receptor B2	0.00E+00	2.08E+04	7.18E+04	3.26E+04	2.26E+04	—	—	0.92	0.41	—	—	3.18	0.12	2.20	0.47	1.44	0.56
O60888	Protein CutA	1.75E+05	1.58E+05	5.81E+05	1.65E+05	1.28E+05	1.11	0.60	1.23	0.53	0.30	0.04	4.54	0.07	3.52	0.38	1.29	0.46
O75015	Low affinity immunoglobulin gamma Fc region receptor III-B	0.00E+00	5.01E+04	1.25E+05	5.39E+04	4.59E+04	—	—	1.09	0.99	—	—	2.73	0.27	2.33	0.45	1.17	0.87
O75131	Copine-3	4.42E+04	3.20E+04	3.45E+05	7.32E+04	7.44E+04	1.38	0.40	0.43	0.08	0.13	0.02	4.63	0.26	4.71	0.49	0.98	0.90
O75309	Cadherin-16	6.75E+04	6.21E+04	9.55E+04	6.60E+04	3.38E+04	1.09	0.69	1.84	0.00	0.71	0.88	2.83	0.10	1.45	0.82	1.95	0.00
O75339	Cartilage intermediate layer protein 1	3.95E+04	3.49E+04	1.81E+05	5.80E+04	6.51E+04	1.13	0.70	0.54	0.03	0.22	0.03	2.78	0.53	3.12	0.62	0.89	0.96
O75340	Programmed cell death protein 6	2.00E+04	2.23E+04	2.23E+05	1.24E+04	2.66E+04	0.90	0.63	0.84	0.92	0.09	0.00	8.39	0.07	17.99	0.07	0.47	0.28
O75368	Adapter SH3BGRL	3.54E+04	2.77E+04	1.40E+05	4.40E+04	1.78E+04	1.28	0.61	1.56	0.02	0.25	0.60	7.87	0.27	3.18	0.74	2.47	0.02
O75629	Protein CREG1	1.82E+05	1.84E+05	1.16E+06	3.09E+05	2.80E+05	0.98	0.63	0.66	0.12	0.16	0.02	4.14	0.29	3.75	0.47	1.10	0.97
O75886	Signal transducing adapter molecule 2	0.00E+00	1.26E+04	6.34E+04	5.16E+04	2.04E+04	—	—	0.62	0.66	—	—	3.11	0.85	1.23	0.71	2.53	0.48
O75891	Cytosolic 10-formyltetrahydrofolate dehydrogenase	6.18E+04	5.12E+04	3.43E+04	4.37E+04	3.24E+04	1.21	0.30	1.58	0.68	1.80	0.04	1.06	0.34	0.78	0.41	1.35	0.72
O95336	6-phosphogluconolactonase	4.27E+05	4.15E+05	2.45E+05	3.47E+05	3.55E+05	1.03	0.87	1.17	0.70	1.75	0.03	0.69	0.14	0.70	0.89	0.98	0.42
O95502	Neuronal pentraxin receptor	0.00E+00	2.20E+04	8.78E+04	2.36E+04	1.48E+04	—	—	1.49	0.06	—	—	5.93	0.23	3.72	0.41	1.59	0.97

O95831	Apoptosis-inducing factor 1, mitochondrial	1.10E+05	7.80E+04	7.30E+05	1.63E+05	9.17E+04	1.41	0.94	0.85	0.78	0.15	0.01	7.96	0.05	4.48	0.18	1.78	0.83
P01009	Alpha-1-antitrypsin	1.73E+06	1.25E+06	6.38E+05	1.09E+06	6.95E+05	1.38	0.07	1.81	0.04	2.72	0.00	0.92	0.59	0.59	0.32	1.57	0.35
P01011	Alpha-1-antichymotrypsin	1.10E+06	8.39E+05	5.02E+05	5.23E+05	5.54E+05	1.31	0.49	1.51	0.56	2.19	0.04	0.91	0.23	0.96	0.84	0.94	0.31
P01225	Follitropin subunit beta	0.00E+00	8.21E+04	3.36E+05	0.00E+00	3.20E+04	—	—	2.56	0.27	—	—	10.50	0.09	—	—	—	—
P01241	Somatotropin	0.00E+00	0.00E+00	3.35E+05	5.95E+04	7.81E+04	—	—	—	—	—	—	4.28	0.37	5.62	0.45	0.76	0.95
P01624	Immunoglobulin kappa variable 3-15	6.33E+05	7.44E+05	1.88E+06	8.15E+05	1.62E+06	0.85	0.93	0.46	0.05	0.34	0.04	1.16	0.77	2.31	0.13	0.50	0.10
P01782	Immunoglobulin heavy variable 3-9	4.55E+04	7.44E+04	1.72E+05	1.66E+05	8.01E+04	0.61	0.72	0.93	0.21	0.26	0.04	2.15	0.56	1.04	0.99	2.08	0.57
P02649	Apolipoprotein E	2.32E+05	1.44E+05	1.27E+05	1.33E+05	1.80E+05	1.61	0.01	0.80	0.38	1.82	0.05	0.71	0.33	0.96	0.62	0.74	0.21
P02671	Fibrinogen alpha chain	3.37E+05	3.15E+05	3.23E+05	2.35E+05	1.22E+05	1.07	0.77	2.58	0.00	1.04	0.76	2.64	0.00	1.37	0.64	1.92	0.00
P02763	Alpha-1-acid glycoprotein 1	4.47E+06	1.78E+07	4.75E+07	1.42E+06	4.24E+06	0.25	0.90	4.19	0.84	0.09	0.04	11.19	0.05	33.55	0.05	0.33	0.36
P02787	Serotransferrin	1.36E+06	4.95E+05	4.81E+05	4.57E+05	6.81E+05	2.75	0.01	0.73	0.45	2.83	0.03	0.71	0.38	1.05	0.92	0.67	0.46
P04080	Cystatin-B	2.25E+04	3.82E+04	2.84E+05	8.99E+04	7.25E+04	0.59	0.15	0.53	0.87	0.08	0.01	3.92	0.23	3.16	0.73	1.24	0.05
P04083	Annexin A1	5.38E+04	2.93E+04	5.91E+05	1.99E+04	8.18E+04	1.84	0.41	0.36	0.27	0.09	0.03	7.22	0.21	29.66	0.13	0.24	0.49
P04216	Thy-1 membrane glycoprotein	8.08E+04	1.29E+05	2.94E+05	2.91E+05	1.51E+05	0.63	0.45	0.86	0.62	0.28	0.03	1.95	0.40	1.01	0.63	1.93	0.13
P04233	HLA class II histocompatibility antigen gamma chain	2.15E+05	4.09E+05	1.26E+06	4.70E+05	7.35E+05	0.53	0.09	0.56	0.66	0.17	0.04	1.71	0.35	2.67	0.78	0.64	0.53
P04632	Calpain small subunit 1	2.84E+04	1.77E+04	3.93E+05	1.90E+04	3.47E+04	1.61	0.05	0.51	0.16	0.07	0.01	11.32	0.11	20.70	0.08	0.55	0.31

P05062	Fructose-bisphosphate aldolase B	2.58E+05	2.47E+05	1.08E+05	2.01E+05	1.67E+05	1.04	0.67	1.48	0.40	2.38	0.01	0.65	0.10	0.54	0.33	1.20	0.99
P05067	Amyloid-beta precursor protein	1.90E+05	1.57E+05	1.03E+06	2.22E+05	2.71E+05	1.21	0.97	0.58	0.97	0.18	0.04	3.79	0.17	4.63	0.49	0.82	0.53
P05981	Serine protease hepsin	2.60E+04	3.66E+04	6.31E+04	4.95E+04	3.91E+04	0.71	0.03	0.94	0.40	0.41	0.02	1.61	0.29	1.27	0.99	1.27	0.32
P06127	T-cell surface glycoprotein CD5	2.82E+04	0.00E+00	4.52E+04	5.83E+04	1.51E+04	—	—	—	—	0.62	0.21	2.99	0.03	0.78	0.58	3.85	0.02
P06734	Low affinity immunoglobulin epsilon Fc receptor	1.43E+05	1.44E+05	3.53E+05	8.27E+04	1.23E+05	0.99	0.04	1.17	0.09	0.40	0.04	2.88	0.13	4.27	0.14	0.68	0.58
P07148	Fatty acid-binding protein, liver	2.32E+05	1.66E+05	1.19E+05	1.85E+05	7.22E+04	1.40	0.45	2.29	0.01	1.94	0.42	1.65	0.62	0.64	0.13	2.57	0.01
P07333	Macrophage colony-stimulating factor 1 receptor	5.04E+04	3.53E+04	1.85E+05	4.92E+04	5.20E+04	1.43	0.17	0.68	0.08	0.27	0.03	3.56	0.17	3.76	0.25	0.95	0.71
P07478	Trypsin-2	2.36E+05	1.73E+05	4.43E+05	1.29E+05	3.42E+05	1.36	0.87	0.51	0.17	0.53	0.03	1.29	0.29	3.44	0.08	0.38	0.34
P07948	Tyrosine-protein kinase Lyn	2.68E+04	2.25E+04	2.53E+05	1.34E+05	3.29E+04	1.19	0.28	0.68	0.61	0.11	0.00	7.69	0.06	1.89	0.86	4.06	0.08
P08174	Complement decay-accelerating factor	6.57E+05	1.03E+06	4.27E+06	9.78E+05	6.26E+05	0.64	0.21	1.64	0.49	0.15	0.03	6.83	0.16	4.37	0.69	1.56	0.29
P08727	Keratin, type I cytoskeletal 19	6.94E+04	6.45E+04	1.60E+05	7.29E+04	3.05E+04	1.08	0.54	2.11	0.12	0.43	0.14	5.25	0.02	2.20	0.71	2.39	0.04
P08887	Interleukin-6 receptor subunit alpha	3.32E+04	3.66E+04	1.24E+05	4.25E+04	3.17E+04	0.91	0.23	1.16	0.47	0.27	0.04	3.91	0.13	2.91	0.56	1.34	0.43
P09131	P3 protein	2.54E+04	2.96E+04	1.55E+05	4.84E+04	2.93E+04	0.86	0.59	1.01	0.29	0.16	0.05	5.28	0.13	3.19	0.80	1.65	0.15
P09237	Matrilysin	1.00E+05	1.22E+05	5.40E+04	7.41E+04	2.69E+04	0.82	0.65	4.53	0.01	1.86	0.34	2.01	0.01	0.73	0.56	2.76	0.01
P09486	SPARC	5.06E+04	3.70E+04	9.40E+04	4.50E+04	3.88E+04	1.37	0.30	0.95	0.55	0.54	0.05	2.42	0.06	2.09	0.18	1.16	0.83
P10253	Lysosomal alpha-glucosidase	6.68E+05	9.77E+05	2.35E+06	7.26E+05	5.90E+05	0.68	0.92	1.66	0.64	0.28	0.01	3.99	0.05	3.24	0.28	1.23	0.47

P10809	60 kDa heat shock protein, mitochondrial	0.00E+00	2.12E+04	4.64E+04	2.70E+04	5.80E+04	—	—	0.37	0.93	—	—	0.80	0.70	1.72	0.97	0.47	0.69
P10909	Clusterin	2.54E+06	1.71E+06	9.72E+05	1.41E+06	1.04E+06	1.49	0.07	1.64	0.16	2.61	0.01	0.93	0.39	0.69	0.37	1.35	0.42
P11117	Lysosomal acid phosphatase	5.55E+05	4.35E+05	1.37E+06	4.55E+05	9.00E+05	1.27	0.56	0.48	0.02	0.41	0.02	1.52	0.45	3.01	0.11	0.50	0.18
P12318	Low affinity immunoglobulin gamma Fc region receptor II-a	3.26E+04	5.43E+04	8.87E+04	9.65E+04	9.47E+03	0.60	0.06	5.74	0.00	0.37	0.20	9.36	0.04	0.92	0.60	10.19	0.01
P12319	High affinity immunoglobulin epsilon receptor subunit alpha	0.00E+00	0.00E+00	3.26E+05	6.24E+04	5.52E+04	—	—	—	—	—	—	5.90	0.34	5.22	0.38	1.13	0.62
P12830	Cadherin-1	8.24E+05	1.41E+06	4.41E+06	1.82E+06	9.84E+05	0.58	0.04	1.44	0.21	0.19	0.02	4.49	0.11	2.42	0.48	1.86	0.57
P13598	Intercellular adhesion molecule 2	3.72E+05	4.03E+05	3.49E+06	4.51E+05	3.21E+05	0.92	0.35	1.26	0.70	0.11	0.02	10.87	0.15	7.74	0.58	1.40	0.32
P13746	HLA class I histocompatibility antigen, A alpha chain	0.00E+00	1.85E+04	1.57E+05	3.53E+04	1.83E+04	—	—	1.01	0.90	—	—	8.58	0.20	4.44	0.64	1.93	0.54
P13798	Acylamino-acid-releasing enzyme	7.61E+04	1.52E+05	6.75E+05	1.30E+05	5.14E+05	0.50	0.42	0.30	0.92	0.11	0.03	1.31	0.36	5.21	0.68	0.25	0.61
P14778	Interleukin-1 receptor type 1	1.93E+04	1.82E+04	5.33E+04	3.77E+04	2.58E+04	1.06	0.95	0.70	0.78	0.36	0.02	2.06	0.12	1.41	0.79	1.46	0.24
P14784	Interleukin-2 receptor subunit beta	4.74E+04	4.82E+04	1.14E+05	9.92E+04	4.95E+04	0.98	0.84	0.97	0.96	0.42	0.04	2.30	0.10	1.15	0.99	2.00	0.11
P15144	Aminopeptidase N	1.91E+05	1.28E+05	1.20E+05	1.36E+05	1.34E+05	1.49	0.03	0.96	0.41	1.60	0.04	0.89	0.23	0.88	1.00	1.01	0.31
P15151	Poliovirus receptor	5.42E+05	6.65E+05	4.05E+06	1.20E+06	7.12E+05	0.81	0.13	0.93	0.45	0.13	0.02	5.68	0.18	3.37	0.82	1.69	0.17
P15907	Beta-galactoside alpha-2,6-sialyltransferase 1	3.31E+04	4.74E+04	4.71E+05	1.09E+05	2.23E+04	0.70	0.26	2.13	0.05	0.07	0.77	21.12	0.51	4.32	0.41	4.89	0.01
P16112	Aggrecan core protein	2.82E+05	2.47E+05	3.13E+06	6.78E+05	4.97E+05	1.14	0.23	0.50	0.94	0.09	0.04	6.29	0.30	4.61	0.99	1.36	0.22
P16401	Histone H1.5	0.00E+00	3.32E+04	4.23E+05	6.27E+04	3.24E+04	—	—	1.02	0.40	—	—	13.03	0.72	6.74	0.93	1.93	0.75

P16444	Dipeptidase 1	4.60E+05	3.40E+05	2.18E+05	2.54E+05	2.96E+05	1.35	0.31	1.15	0.67	2.11	0.04	0.74	0.22	0.86	0.87	0.86	0.42
P17931	Galectin-3	7.34E+04	8.66E+04	3.40E+05	1.00E+05	9.68E+04	0.85	0.24	0.89	0.33	0.22	0.01	3.52	0.32	3.39	0.57	1.04	0.73
P18428	Lipopolysaccharide-binding protein	6.44E+04	5.96E+04	9.89E+04	2.01E+04	1.98E+04	1.08	0.85	3.02	0.00	0.65	0.68	5.00	0.03	4.93	0.11	1.01	0.87
P18627	Lymphocyte activation gene 3 protein	0.00E+00	1.32E+04	7.36E+04	4.24E+04	1.91E+04	—	—	0.69	0.73	—	—	3.84	0.53	1.73	0.75	2.22	0.24
P19022	Cadherin-2	3.79E+05	4.85E+05	2.36E+06	8.27E+05	4.33E+05	0.78	0.17	1.12	0.63	0.16	0.00	5.45	0.06	2.85	0.59	1.91	0.19
P19652	Alpha-1-acid glycoprotein 2	7.37E+05	2.12E+06	6.75E+06	6.32E+05	8.29E+05	0.35	0.87	2.55	0.88	0.11	0.01	8.15	0.07	10.68	0.14	0.76	0.60
P19801	Amiloride-sensitive amine oxidase [copper-containing]	2.15E+05	2.07E+05	1.12E+05	1.50E+05	1.56E+05	1.04	0.72	1.32	0.58	1.93	0.02	0.71	0.16	0.74	0.64	0.96	0.50
P20138	Myeloid cell surface antigen CD33	4.85E+04	6.33E+04	1.44E+05	9.71E+04	6.66E+04	0.77	0.41	0.95	0.48	0.34	0.01	2.15	0.18	1.48	0.69	1.46	0.44
P20774	Mimecan	4.21E+04	4.98E+04	3.04E+04	6.37E+04	2.59E+04	0.84	0.17	1.92	0.01	1.38	0.65	1.17	0.47	0.48	0.09	2.46	0.03
P20933	N(4)-(beta-N-acetylglucosaminyl)-L-asparaginase	5.47E+04	7.74E+04	1.81E+05	7.91E+04	3.54E+04	0.71	0.45	2.18	0.04	0.30	0.08	5.10	0.03	2.28	0.63	2.23	0.11
P21291	Cysteine and glycine-rich protein 1	2.04E+04	1.31E+04	1.75E+05	2.88E+04	2.28E+04	1.56	0.93	0.57	0.97	0.12	0.02	7.67	0.12	6.06	0.63	1.27	0.25
P21583	Kit ligand	0.00E+00	2.21E+04	1.22E+05	4.09E+04	2.21E+04	—	—	1.00	0.64	—	—	5.52	0.08	2.98	0.87	1.85	0.07
P21926	CD9 antigen	1.02E+05	7.95E+04	4.06E+04	1.00E+05	7.54E+04	1.29	0.36	1.05	0.64	2.52	0.02	0.54	0.30	0.41	0.26	1.33	0.60
P22894	Neutrophil collagenase	0.00E+00	1.76E+04	7.34E+04	2.90E+04	1.97E+04	—	—	0.89	0.84	—	—	3.72	0.70	2.53	0.83	1.47	0.93
P23467	Receptor-type tyrosine-protein phosphatase beta	0.00E+00	0.00E+00	1.11E+05	2.24E+04	3.96E+04	—	—	—	—	—	—	2.79	0.87	4.93	0.58	0.57	0.53
P26842	CD27 antigen	4.04E+05	6.72E+05	2.80E+06	9.16E+05	1.25E+06	0.60	0.46	0.54	0.42	0.14	0.03	2.25	0.55	3.06	0.79	0.73	0.78

P28300	Protein-lysine 6-oxidase	1.11E+05	1.47E+05	3.35E+05	1.35E+05	1.69E+05	0.75	0.30	0.87	0.55	0.33	0.03	1.98	0.26	2.48	0.16	0.80	0.31
P28676	Grancalcin	0.00E+00	1.66E+04	5.41E+04	3.25E+04	1.58E+04	—	—	1.05	0.81	—	—	3.42	0.97	1.66	0.31	2.06	0.07
P30086	Phosphatidylethanolamine-binding protein 1	5.18E+05	5.91E+05	5.44E+05	6.09E+05	3.31E+05	0.88	0.51	1.79	0.03	0.95	0.34	1.64	0.77	0.89	0.36	1.84	0.03
P30453	HLA class I histocompatibility antigen, A alpha chain	3.94E+04	4.30E+04	1.49E+05	4.84E+04	8.80E+04	0.92	0.94	0.49	0.79	0.26	0.03	1.69	0.15	3.08	0.47	0.55	0.47
P30530	Tyrosine-protein kinase receptor UFO	1.22E+06	1.24E+06	5.51E+06	2.61E+06	1.91E+06	0.99	0.36	0.65	0.23	0.22	0.01	2.89	0.26	2.11	0.93	1.37	0.22
P31785	Cytokine receptor common subunit gamma	0.00E+00	1.17E+04	4.32E+04	2.00E+04	1.71E+04	—	—	0.69	0.38	—	—	2.53	0.11	2.16	0.40	1.17	0.64
P31997	Carcinoembryonic antigen-related cell adhesion molecule 8	4.37E+04	4.49E+04	1.14E+05	6.55E+04	2.33E+04	0.97	0.25	1.93	0.03	0.38	0.43	4.90	0.21	1.75	0.60	2.81	0.01
P32942	Intercellular adhesion molecule 3	5.72E+04	5.25E+04	3.25E+05	7.14E+04	5.09E+04	1.09	0.70	1.03	0.58	0.18	0.05	6.39	0.11	4.56	0.69	1.40	0.16
P35556	Fibrillin-2	2.87E+04	4.14E+04	1.09E+05	3.47E+04	2.01E+04	0.69	0.43	2.06	0.00	0.26	0.17	5.40	0.01	3.14	0.81	1.72	0.03
P35613	Basigin	1.07E+05	1.53E+05	5.65E+05	1.38E+05	1.74E+05	0.70	0.14	0.88	0.86	0.19	0.02	3.24	0.29	4.09	0.57	0.79	0.63
P35916	Vascular endothelial growth factor receptor 3	0.00E+00	1.56E+04	2.16E+04	0.00E+00	1.27E+04	—	—	1.23	0.58	—	—	1.70	0.70	—	—	—	—
P36578	60S ribosomal protein L4	0.00E+00	0.00E+00	9.40E+04	2.05E+04	9.34E+03	—	—	—	—	—	—	10.07	0.41	4.58	0.91	2.20	0.23
P36897	TGF-beta receptor type-1	0.00E+00	1.86E+04	7.41E+04	4.21E+04	1.30E+04	—	—	1.43	0.21	—	—	5.71	0.03	1.76	0.85	3.25	0.07
P39059	Collagen alpha-1	1.68E+05	1.83E+05	3.69E+05	3.72E+05	2.83E+05	0.92	0.67	0.65	0.04	0.46	0.03	1.30	0.68	0.99	0.73	1.31	0.87
P41222	Prostaglandin-H2 D-isomerase	2.01E+07	2.45E+07	7.17E+07	3.37E+07	5.95E+06	0.82	0.20	4.12	0.01	0.28	0.63	12.05	0.19	2.13	0.46	5.67	0.01
P42357	Histidine ammonia-lyase	0.00E+00	0.00E+00	3.33E+04	1.11E+04	2.71E+04	—	—	—	—	—	—	1.23	0.44	3.00	0.61	0.41	0.09

P43490	Nicotinamide phosphoribosyltransferase	0.00E+00	1.85E+04	9.87E+04	0.00E+00	1.54E+04	—	—	1.20	0.63	—	—	6.40	0.32	—	—	—	—
P43626	Killer cell immunoglobulin-like receptor 2DL1	0.00E+00	4.88E+04	9.12E+04	0.00E+00	1.12E+05	—	—	0.43	0.38	—	—	0.81	0.62	—	—	—	—
P47756	F-actin-capping protein subunit beta	4.79E+04	3.52E+04	5.87E+05	6.90E+04	1.58E+05	1.36	0.22	0.22	0.32	0.08	0.04	3.73	0.34	8.51	0.52	0.44	0.81
P48643	T-complex protein 1 subunit epsilon	2.89E+04	4.84E+04	2.39E+05	2.10E+04	2.47E+05	0.60	0.67	0.20	0.88	0.12	0.03	0.97	0.38	11.35	0.38	0.09	0.97
P49720	Proteasome subunit beta type-3	0.00E+00	1.49E+04	8.00E+04	0.00E+00	3.32E+04	—	—	0.45	0.15	—	—	2.41	0.85	—	—	—	—
P49841	Glycogen synthase kinase-3 beta	0.00E+00	1.05E+04	1.64E+05	1.77E+04	2.02E+04	—	—	0.52	0.64	—	—	8.14	0.69	9.29	0.94	0.88	0.54
P49908	Selenoprotein P	1.24E+05	1.75E+05	2.18E+06	9.50E+04	7.96E+05	0.71	0.85	0.22	0.23	0.06	0.02	2.74	0.42	22.95	0.41	0.12	0.85
P50053	Ketohexokinase	5.58E+04	6.85E+04	3.68E+04	6.21E+04	2.85E+04	0.81	0.61	2.41	0.14	1.52	0.03	1.29	0.97	0.59	0.08	2.18	0.02
P50135	Histamine N-methyltransferase	0.00E+00	2.49E+04	1.64E+05	2.13E+04	4.26E+04	—	—	0.59	0.85	—	—	3.85	0.27	7.71	0.48	0.50	0.75
P55103	Inhibin beta C chain	3.17E+04	4.22E+04	2.11E+05	3.88E+04	5.96E+04	0.75	0.22	0.71	0.74	0.15	0.01	3.54	0.19	5.44	0.23	0.65	0.80
P55210	Caspase-7	2.71E+06	9.09E+04	1.11E+05	5.56E+04	1.59E+04	29.77	0.67	5.72	0.01	24.43	0.59	6.97	0.20	1.99	0.73	3.50	0.04
P55786	Puromycin-sensitive aminopeptidase	4.08E+04	3.63E+05	1.52E+06	1.83E+04	2.78E+04	0.11	0.00	13.06	0.02	0.03	0.01	54.61	0.05	82.96	0.15	0.66	0.76
P55899	IgG receptor FcRn large subunit p51	0.00E+00	1.79E+04	7.72E+04	0.00E+00	2.06E+04	—	—	0.87	0.66	—	—	3.75	0.75	—	—	—	—
P56537	Eukaryotic translation initiation factor 6	3.80E+05	2.40E+05	1.44E+05	3.06E+05	1.82E+05	1.58	0.04	1.32	0.28	2.65	0.00	0.79	0.31	0.47	0.42	1.68	0.74
P57087	Junctional adhesion molecule B	2.01E+04	1.96E+04	3.69E+05	2.09E+05	2.10E+04	1.02	0.36	0.94	0.33	0.05	0.04	17.61	0.14	1.76	0.92	9.99	0.13
P60033	CD81 antigen	5.02E+04	2.37E+04	8.96E+04	5.02E+04	1.96E+04	2.12	0.03	1.21	0.93	0.56	0.61	4.57	0.14	1.79	0.83	2.56	0.05

P60842	Eukaryotic initiation factor 4A-I	4.69E+04	5.05E+04	5.48E+05	1.19E+05	3.13E+04	0.93	0.86	1.61	0.34	0.09	0.03	17.49	0.06	4.59	0.76	3.81	0.07
P60953	Cell division control protein 42 homolog	4.56E+04	2.40E+04	4.07E+05	2.16E+04	3.19E+04	1.90	1.00	0.75	0.81	0.11	0.03	12.76	0.07	18.86	0.21	0.68	0.74
P61160	Actin-related protein 2	3.87E+04	3.53E+04	6.46E+05	7.87E+04	6.28E+04	1.10	0.32	0.56	0.30	0.06	0.02	10.29	0.22	8.21	0.71	1.25	0.27
P62140	Serine/threonine-protein phosphatase PP1-beta catalytic subunit	2.16E+04	1.66E+04	1.73E+05	1.43E+04	2.27E+04	1.30	0.33	0.73	0.51	0.12	0.03	7.63	0.12	12.09	0.23	0.63	0.93
P62820	Ras-related protein Rab-1A	6.29E+04	2.84E+04	2.54E+05	2.59E+04	2.65E+04	2.21	0.04	1.07	0.44	0.25	0.07	9.60	0.04	9.81	0.12	0.98	0.48
P80404	4-aminobutyrate aminotransferase, mitochondrial	0.00E+00	9.89E+04	1.43E+05	5.19E+04	2.39E+04	—	—	4.15	0.03	—	—	5.99	0.20	2.75	0.61	2.17	0.05
P82987	ADAMTS-like protein 3	0.00E+00	2.39E+04	7.19E+04	2.31E+04	4.84E+04	—	—	0.49	0.92	—	—	1.48	0.95	3.11	0.63	0.48	0.54
P98172	Ephrin-B1	9.71E+04	1.70E+05	5.34E+05	2.90E+05	1.19E+05	0.57	0.06	1.43	0.45	0.18	0.01	4.49	0.11	1.84	0.86	2.44	0.13
Q00796	Sorbitol dehydrogenase	6.07E+04	4.03E+04	2.21E+05	7.61E+04	2.21E+04	1.50	0.39	1.82	0.41	0.27	0.10	10.00	0.03	2.90	0.71	3.44	0.02
Q01151	CD83 antigen	0.00E+00	1.50E+04	9.78E+04	1.31E+04	7.44E+03	—	—	2.02	0.02	—	—	13.14	0.18	7.45	0.96	1.76	0.11
Q06124	Tyrosine-protein phosphatase non-receptor type 11	3.01E+04	2.17E+05	5.28E+05	3.03E+04	4.53E+04	0.14	0.02	4.79	0.25	0.06	0.02	11.66	0.19	17.41	0.28	0.67	0.86
Q07075	Glutamyl aminopeptidase	1.73E+05	1.72E+05	3.81E+05	9.86E+04	5.03E+04	1.01	0.94	3.41	0.00	0.45	0.71	7.58	0.16	3.87	0.92	1.96	0.03
Q07960	Rho GTPase-activating protein 1	3.15E+04	2.75E+04	2.06E+05	5.25E+04	4.77E+04	1.15	0.42	0.58	0.23	0.15	0.01	4.31	0.14	3.91	0.42	1.10	0.61
Q08629	Testican-1	3.04E+04	3.38E+04	1.30E+05	3.49E+04	4.36E+04	0.90	0.90	0.78	0.57	0.23	0.04	2.99	0.33	3.73	0.47	0.80	1.00
Q10588	ADP-ribosyl cyclase/cyclic ADP-ribose hydrolase 2	5.05E+04	5.03E+04	1.40E+05	6.20E+04	1.57E+04	1.00	0.84	3.20	0.04	0.36	0.11	8.92	0.01	2.26	0.50	3.94	0.11
Q12906	Interleukin enhancer-binding factor 3	1.90E+05	1.35E+05	6.56E+05	4.46E+04	7.13E+05	1.41	0.60	0.19	0.00	0.29	0.31	0.92	0.34	14.71	0.18	0.06	0.00

Q13277	Syntaxin-3	1.45E+04	1.32E+04	1.06E+05	8.98E+03	1.78E+04	1.10	0.32	0.74	0.49	0.14	0.05	5.94	0.20	11.77	0.20	0.50	0.61
Q13705	Activin receptor type-2B	0.00E+00	1.81E+04	1.68E+05	3.86E+04	1.61E+04	—	—	1.12	0.83	—	—	10.43	0.17	4.37	0.74	2.39	0.29
Q14050	Collagen alpha-3	2.88E+04	3.81E+04	1.44E+05	1.98E+04	3.69E+04	0.76	0.85	1.03	0.81	0.20	0.04	3.90	0.14	7.25	0.24	0.54	0.89
Q14055	Collagen alpha-2	0.00E+00	1.67E+04	1.29E+05	2.67E+04	3.04E+04	—	—	0.55	0.73	—	—	4.25	0.66	4.83	0.90	0.88	0.78
Q14165	Malectin	2.17E+04	1.88E+04	1.92E+05	6.39E+04	3.52E+04	1.15	0.78	0.53	0.19	0.11	0.03	5.44	0.36	3.00	0.95	1.81	0.39
Q14314	Fibroleukin	2.99E+04	2.88E+04	7.58E+05	1.26E+04	2.72E+04	1.04	0.92	1.06	0.96	0.04	0.03	27.81	0.15	60.12	0.12	0.46	0.13
Q14677	Clathrin interactor 1	0.00E+00	0.00E+00	1.19E+05	4.23E+04	3.08E+04	—	—	—	—	—	—	3.85	0.17	2.81	0.66	1.37	0.38
Q14982	Opioid-binding protein/cell adhesion molecule	2.53E+05	3.07E+05	4.99E+05	4.88E+05	3.57E+05	0.83	0.42	0.86	0.24	0.51	0.04	1.40	0.71	1.02	0.90	1.37	0.89
Q15043	Metal cation symporter ZIP14	1.73E+04	1.93E+04	9.85E+04	2.30E+04	2.11E+04	0.90	0.82	0.91	0.76	0.18	0.03	4.67	0.11	4.28	0.36	1.09	0.70
Q15198	Platelet-derived growth factor receptor-like protein	2.71E+04	3.33E+04	1.86E+05	1.64E+04	4.92E+04	0.81	0.78	0.68	0.91	0.15	0.01	3.77	0.11	11.30	0.09	0.33	0.46
Q15751	Probable E3 ubiquitin-protein ligase HERC1	0.00E+00	1.16E+06	1.33E+06	6.44E+05	1.79E+05	—	—	6.48	0.29	—	—	7.41	0.69	2.06	0.90	3.59	0.82
Q15831	Serine/threonine-protein kinase STK11	1.71E+04	1.24E+04	1.96E+05	1.64E+04	1.74E+04	1.38	0.46	0.71	0.47	0.09	0.01	11.23	0.06	11.99	0.29	0.94	0.45
Q16543	Hsp90 co-chaperone Cdc37	0.00E+00	4.15E+04	1.45E+05	0.00E+00	4.38E+04	—	—	0.95	0.75	—	—	3.30	0.60	—	—	—	—
Q24JP5	Transmembrane protein 132A	6.54E+04	5.67E+04	7.96E+04	3.35E+05	2.59E+04	1.15	0.97	2.19	0.02	0.82	0.71	3.07	0.08	0.24	0.27	12.91	0.01
Q5VW32	BRO1 domain-containing protein BROX	2.59E+05	1.89E+05	1.50E+05	3.85E+05	1.70E+05	1.38	0.07	1.11	0.49	1.73	0.02	0.88	0.19	0.39	0.10	2.27	0.02
Q66K79	Carboxypeptidase Z	0.00E+00	4.70E+04	3.50E+04	2.40E+04	2.42E+04	—	—	1.94	0.09	—	—	1.44	0.29	1.46	0.75	0.99	0.55

Q6FHJ7	Secreted frizzled-related protein 4	0.00E+00	4.86E+04	3.42E+05	4.49E+04	3.65E+04	—	—	1.33	0.91	—	—	9.38	0.11	7.63	0.69	1.23	0.17
Q6P1N0	Coiled-coil and C2 domain-containing protein 1A	3.32E+04	2.41E+04	1.31E+05	3.80E+04	5.50E+04	1.37	0.06	0.44	0.53	0.25	0.05	2.39	0.17	3.45	0.45	0.69	0.55
Q6P4A8	Phospholipase B-like 1	0.00E+00	0.00E+00	1.07E+05	5.94E+04	1.90E+04	—	—	—	—	—	—	5.62	0.51	1.80	0.58	3.12	0.14
Q6UW49	Sperm equatorial segment protein 1	0.00E+00	0.00E+00	6.12E+04	1.15E+04	1.96E+04	—	—	—	—	—	—	3.12	0.15	5.33	0.09	0.59	0.51
Q6ZNA5	Ferric-chelate reductase 1	7.86E+04	1.50E+05	5.10E+05	1.44E+05	2.25E+05	0.52	0.23	0.67	0.45	0.15	0.00	2.27	0.24	3.55	0.31	0.64	0.90
Q6ZQN7	Solute carrier organic anion transporter family member 4C1	2.52E+04	2.05E+04	8.32E+04	3.28E+04	3.62E+04	1.23	0.62	0.57	0.72	0.30	0.05	2.30	0.21	2.54	0.44	0.91	0.79
Q86V85	Integral membrane protein GPR180	2.89E+04	3.10E+04	1.59E+05	3.36E+04	3.00E+04	0.93	0.77	1.03	0.59	0.18	0.04	5.30	0.24	4.72	0.55	1.12	0.50
Q86Y78	Ly6/PLAUR domain-containing protein 6	0.00E+00	2.68E+04	5.71E+04	2.42E+04	3.43E+04	—	—	0.78	0.61	—	—	1.67	0.86	2.36	0.90	0.70	0.74
Q86YQ8	Copine-8	4.74E+04	2.58E+04	2.56E+05	1.58E+04	3.85E+04	1.84	0.40	0.67	0.52	0.19	0.04	6.64	0.11	16.18	0.07	0.41	0.34
Q8N114	Protein shisa-5	0.00E+00	3.51E+05	1.63E+06	0.00E+00	6.92E+05	—	—	0.51	0.15	—	—	2.36	0.74	—	—	—	—
Q8N2U0	Transmembrane protein 256	2.87E+05	1.22E+05	5.49E+05	9.00E+04	2.12E+05	2.35	0.03	0.58	0.14	0.52	0.11	2.60	0.12	6.11	0.04	0.43	0.50
Q8NFQ8	Torsin-1A-interacting protein 2	0.00E+00	1.35E+04	1.88E+05	0.00E+00	2.79E+04	—	—	0.48	0.09	—	—	6.75	0.43	—	—	—	—
Q8NHJ6	Leukocyte immunoglobulin-like receptor subfamily B member 4	7.94E+04	8.19E+04	2.75E+05	1.21E+05	5.10E+04	0.97	0.09	1.61	0.03	0.29	0.09	5.40	0.09	2.28	0.87	2.37	0.01
Q8NI32	Ly6/PLAUR domain-containing protein 6B	0.00E+00	2.20E+04	8.74E+04	1.76E+04	1.58E+04	—	—	1.39	0.35	—	—	5.54	0.63	4.96	0.33	1.12	0.15
Q8TBP5	Membrane protein FAM174A	0.00E+00	0.00E+00	7.74E+04	1.49E+04	6.71E+04	—	—	—	—	—	—	1.15	0.66	5.19	0.36	0.22	0.51
Q8TE58	A disintegrin and metalloproteinase with thrombospondin motifs 15	2.13E+05	2.12E+05	1.83E+06	1.36E+05	2.73E+05	1.00	0.53	0.78	0.43	0.12	0.02	6.72	0.08	13.42	0.21	0.50	0.73

Q8WVV5	Butyrophilin subfamily 2 member A2	1.10E+05	1.52E+05	3.19E+05	2.20E+05	1.07E+05	0.73	0.22	1.42	0.61	0.34	0.04	2.99	0.12	1.45	0.93	2.06	0.11
Q92542	Nicastrin	3.19E+04	4.86E+04	9.58E+04	2.81E+04	3.23E+04	0.66	0.39	1.51	0.68	0.33	0.01	2.96	0.06	3.41	0.10	0.87	0.36
Q92563	Testican-2	5.98E+04	4.19E+04	9.93E+04	3.07E+04	3.61E+04	1.43	0.18	1.16	0.48	0.60	0.19	2.75	0.03	3.23	0.04	0.85	0.57
Q93088	Betaine--homocysteine S-methyltransferase 1	3.53E+05	2.72E+05	1.73E+05	1.99E+05	2.26E+05	1.30	0.05	1.20	0.80	2.04	0.01	0.76	0.47	0.87	0.88	0.88	0.67
Q96A25	Transmembrane protein 106A	5.30E+04	7.73E+04	1.20E+06	5.67E+04	6.56E+04	0.69	0.23	1.18	0.78	0.04	0.01	18.37	0.15	21.26	0.17	0.86	0.34
Q96AP7	Endothelial cell-selective adhesion molecule	7.32E+04	1.12E+05	3.11E+05	1.58E+05	1.85E+05	0.65	0.15	0.60	0.37	0.24	0.01	1.68	0.53	1.97	0.61	0.85	0.97
Q96CN7	Isochorismatase domain-containing protein 1	0.00E+00	2.10E+04	5.09E+05	8.13E+04	4.23E+04	—	—	0.50	0.87	—	—	12.04	0.12	6.25	0.93	1.93	0.04
Q96CS7	Pleckstrin homology domain-containing family B member 2	2.37E+04	1.74E+04	1.46E+05	2.80E+04	2.82E+04	1.37	0.43	0.62	0.08	0.16	0.04	5.17	0.28	5.20	0.53	0.99	0.75
Q96EG1	Arylsulfatase G	0.00E+00	1.05E+04	9.01E+04	1.46E+04	1.53E+04	—	—	0.69	0.86	—	—	5.88	0.55	6.17	0.82	0.95	0.29
Q96GP6	Scavenger receptor class F member 2	0.00E+00	2.82E+04	4.21E+04	1.97E+04	1.45E+04	—	—	1.94	0.06	—	—	2.90	0.79	2.13	0.61	1.36	0.20
Q96JY6	PDZ and LIM domain protein 2	0.00E+00	2.54E+04	7.97E+04	0.00E+00	2.76E+04	—	—	0.92	0.30	—	—	2.89	0.97	—	—	—	—
Q99650	Oncostatin-M-specific receptor subunit beta	2.17E+04	1.58E+04	8.97E+04	2.81E+04	2.39E+04	1.38	0.58	0.66	0.99	0.24	0.05	3.76	0.12	3.20	0.30	1.17	0.94
Q99969	Retinoic acid receptor responder protein 2	6.57E+04	9.25E+04	1.80E+05	7.58E+04	6.85E+04	0.71	0.10	1.35	0.55	0.36	0.03	2.63	0.17	2.38	0.22	1.11	0.38
Q9BQI0	Allograft inflammatory factor 1-like	0.00E+00	1.92E+04	7.60E+04	2.32E+04	1.99E+04	—	—	0.96	0.57	—	—	3.81	0.19	3.28	0.80	1.16	0.23
Q9BRK5	45 kDa calcium-binding protein	5.68E+04	2.54E+04	1.41E+05	3.54E+04	3.34E+04	2.24	0.02	0.76	0.23	0.40	0.15	4.23	0.05	3.99	0.23	1.06	0.56
Q9BTN0	Leucine-rich repeat and fibronectin type-III domain-containing protein 3	0.00E+00	1.53E+04	7.29E+04	4.77E+04	9.40E+03	—	—	1.63	0.28	—	—	7.75	0.10	1.53	0.57	5.07	0.01

Q9BVM4	Gamma-glutamylaminecyclotransferase	6.23E+04	3.09E+04	9.06E+04	2.47E+04	6.90E+04	2.02	0.10	0.45	0.01	0.69	0.97	1.31	0.42	3.67	0.38	0.36	0.03
Q9BW04	Specifically androgen-regulated gene protein	2.40E+04	3.88E+04	3.37E+05	2.48E+05	4.49E+04	0.62	0.40	0.86	0.28	0.07	0.04	7.51	0.12	1.36	0.78	5.51	0.05
Q9BW30	Tubulin polymerization-promoting protein family member 3	3.92E+04	3.06E+04	1.97E+05	3.20E+04	4.55E+04	1.28	0.97	0.67	0.65	0.20	0.01	4.33	0.10	6.16	0.10	0.70	0.50
Q9BXI6	TBC1 domain family member 10A	3.18E+04	2.59E+04	2.19E+05	4.34E+04	5.90E+04	1.23	0.10	0.44	0.21	0.14	0.01	3.72	0.23	5.05	0.52	0.74	0.57
Q9BXN2	C-type lectin domain family 7 member A	3.37E+04	5.70E+04	3.52E+05	1.12E+05	5.86E+04	0.59	0.04	0.97	0.05	0.10	0.01	6.01	0.06	3.15	0.80	1.91	0.01
Q9H0X4	Protein FAM234A	2.38E+04	2.40E+04	7.73E+04	4.00E+04	2.18E+04	0.99	0.67	1.10	0.90	0.31	0.04	3.54	0.12	1.93	0.95	1.83	0.08
Q9H444	Charged multivesicular body protein 4b	4.74E+04	3.01E+04	4.08E+05	3.08E+04	1.13E+05	1.57	0.07	0.27	0.03	0.12	0.01	3.61	0.32	13.23	0.13	0.27	0.34
Q9H5Y7	SLIT and NTRK-like protein 6	0.00E+00	1.33E+04	7.94E+04	0.00E+00	3.12E+04	—	—	0.42	0.33	—	—	2.55	0.59	—	—	—	—
Q9H6S3	Epidermal growth factor receptor kinase substrate 8-like protein 2	6.74E+04	5.17E+04	9.49E+04	9.37E+04	2.99E+04	1.30	0.07	1.73	0.04	0.71	0.93	3.18	0.08	1.01	0.49	3.14	0.01
Q9H6X2	Anthrax toxin receptor 1	1.29E+05	1.16E+05	1.37E+05	1.18E+05	2.84E+05	1.12	0.91	0.41	0.00	0.94	0.92	0.48	0.04	1.16	0.88	0.42	0.03
Q9H6Y7	E3 ubiquitin-protein ligase RNF167	3.92E+04	3.96E+04	5.85E+05	6.97E+04	8.75E+04	0.99	0.61	0.45	0.85	0.07	0.01	6.68	0.14	8.39	0.45	0.80	0.54
Q9H756	Leucine-rich repeat-containing protein 19	7.34E+04	7.41E+04	5.11E+05	2.19E+05	1.16E+05	0.99	0.94	0.64	0.85	0.14	0.01	4.40	0.10	2.33	0.53	1.89	0.45
Q9HB40	Retinoid-inducible serine carboxypeptidase	1.24E+05	1.10E+05	6.93E+04	7.25E+04	8.80E+04	1.13	0.28	1.25	0.63	1.78	0.02	0.79	0.10	0.96	0.79	0.82	0.15
Q9HBB8	Cadherin-related family member 5	5.69E+04	6.41E+04	2.79E+05	9.24E+04	6.86E+04	0.89	0.32	0.93	0.36	0.20	0.05	4.06	0.16	3.02	0.75	1.35	0.23
Q9HBH0	Rho-related GTP-binding protein RhoF	3.17E+04	2.84E+04	1.16E+05	5.48E+04	4.44E+04	1.12	0.73	0.64	0.30	0.27	0.02	2.61	0.21	2.11	0.48	1.24	0.73
Q9NP79	Vacuolar protein sorting-associated protein VTA1 homolog	1.95E+05	8.33E+04	2.26E+05	8.68E+04	9.26E+04	2.34	0.01	0.90	0.29	0.86	0.39	2.45	0.05	2.61	0.12	0.94	0.55

Q9NU53	Glycoprotein integral membrane protein 1	1.19E+05	1.42E+05	4.66E+05	2.69E+05	1.92E+05	0.84	0.20	0.74	0.78	0.26	0.01	2.43	0.16	1.73	0.74	1.40	0.32
Q9NZN3	EH domain-containing protein 3	5.01E+04	3.34E+04	3.63E+05	2.75E+04	5.02E+04	1.50	0.05	0.67	0.08	0.14	0.02	7.24	0.07	13.18	0.06	0.55	0.19
Q9P2R3	Rabankyrin-5	6.34E+04	5.72E+04	1.43E+05	4.71E+04	1.17E+04	1.11	0.40	4.89	0.01	0.44	0.45	12.24	0.04	3.03	0.96	4.03	0.05
Q9UBP0	Spastin	3.53E+04	3.11E+04	2.56E+05	2.15E+04	5.12E+04	1.14	0.26	0.61	0.27	0.14	0.04	5.00	0.24	11.89	0.15	0.42	0.35
Q9UBR1	Beta-ureidopropionase	1.79E+05	4.29E+05	2.11E+06	2.39E+05	1.36E+05	0.42	0.07	3.16	0.27	0.08	0.02	15.58	0.10	8.85	0.58	1.76	0.36
Q9UBV8	Peflin	3.44E+04	4.00E+04	1.00E+05	3.81E+04	7.17E+04	0.86	0.67	0.56	0.34	0.34	0.02	1.40	0.39	2.64	0.17	0.53	0.62
Q9UIV8	Serpin B13	0.00E+00	0.00E+00	1.82E+05	1.35E+04	2.39E+04	—	—	—	—	—	—	7.60	0.22	13.47	0.33	0.56	0.98
Q9UKK9	ADP-sugar pyrophosphatase	1.66E+04	2.54E+04	7.10E+04	3.24E+04	1.17E+04	0.65	0.16	2.17	0.02	0.23	0.23	6.06	0.11	2.19	0.80	2.76	0.03
Q9ULI3	Protein HEG homolog 1	8.40E+04	1.65E+05	1.08E+06	1.21E+05	2.09E+05	0.51	0.66	0.79	0.50	0.08	0.01	5.19	0.36	8.96	0.49	0.58	0.92
Q9ULK6	RING finger protein 150	4.42E+04	3.63E+04	1.81E+05	4.14E+04	7.25E+04	1.22	0.10	0.50	0.19	0.24	0.04	2.49	0.31	4.36	0.23	0.57	0.52
Q9UM22	Mammalian ependymin-related protein 1	3.99E+04	7.02E+04	1.21E+05	6.85E+04	4.52E+04	0.57	0.36	1.55	0.36	0.33	0.02	2.69	0.11	1.77	0.73	1.52	0.22
Q9UN37	Vacuolar protein sorting-associated protein 4A	2.14E+05	1.25E+05	1.55E+05	2.47E+05	1.06E+05	1.72	0.01	1.18	0.64	1.38	0.40	1.46	0.34	0.63	0.13	2.34	0.00
Q9UN74	Protocadherin alpha-4	2.29E+04	2.43E+04	8.71E+04	2.74E+04	1.16E+04	0.94	0.95	2.09	0.03	0.26	0.26	7.50	0.06	3.18	0.88	2.36	0.04
Q9UQP3	Tenascin-N	0.00E+00	0.00E+00	1.42E+05	1.62E+04	3.81E+04	—	—	—	—	—	—	3.73	0.45	8.76	0.33	0.43	0.63
Q9Y376	Calcium-binding protein 39	2.32E+04	1.56E+04	7.77E+05	1.44E+04	3.11E+04	1.49	0.17	0.50	0.11	0.03	0.03	25.01	0.21	53.96	0.17	0.46	0.27
Q9Y3Q3	Transmembrane emp24 domain-containing protein 3	0.00E+00	0.00E+00	6.52E+04	3.59E+04	4.98E+04	—	—	—	—	—	—	1.31	0.58	1.81	0.63	0.72	0.95

Q9Y4C0	Neurexin-3	4.25E+04	5.62E+04	1.53E+05	1.11E+05	8.30E+04	0.76	0.07	0.68	0.93	0.28	0.02	1.85	0.39	1.38	0.91	1.34	0.49
Q9Y5K2	Kallikrein-4	0.00E+00	0.00E+00	4.65E+04	1.77E+04	1.51E+04	—	—	—	—	—	—	3.09	0.16	2.63	0.89	1.17	0.11
Q9Y653	Adhesion G-protein coupled receptor G1	4.47E+04	5.83E+04	1.93E+05	7.98E+04	4.09E+04	0.77	0.17	1.43	0.18	0.23	0.01	4.72	0.08	2.42	0.70	1.95	0.07
A0A075B6S5	Immunoglobulin kappa variable 1-27	2.13E+04	5.10E+04	1.82E+05	3.16E+04	0.00E+00	0.42	0.01	—	—	0.12	0.15	—	—	5.77	0.96	—	—
A0A0C4DH33	Immunoglobulin heavy variable 1-24	2.10E+04	0.00E+00	1.25E+05	2.68E+04	0.00E+00	—	—	—	—	0.17	0.47	—	—	4.68	0.85	—	—
O00267	Transcription elongation factor SPT5	3.55E+04	3.27E+04	6.86E+04	1.69E+04	0.00E+00	1.09	0.87	—	—	0.52	0.53	—	—	4.07	0.97	—	—
O43657	Tetraspanin-6	1.68E+04	1.21E+04	7.17E+04	0.00E+00	0.00E+00	1.38	0.42	—	—	0.23	0.61	—	—	—	—	—	—
O95989	Diphosphoinositol polyphosphate phosphohydrolase 1	2.04E+04	1.47E+04	5.91E+04	0.00E+00	0.00E+00	1.39	0.64	—	—	0.34	0.45	—	—	—	—	—	—
P00167	Cytochrome b5	1.88E+04	1.73E+04	5.68E+04	4.10E+04	0.00E+00	1.09	0.58	—	—	0.33	0.36	—	—	1.38	0.27	—	—
P00326	Alcohol dehydrogenase 1C	2.52E+04	3.03E+04	1.19E+05	0.00E+00	0.00E+00	0.83	0.19	—	—	0.21	0.22	—	—	—	—	—	—
P00505	Aspartate aminotransferase, mitochondrial	1.92E+04	1.90E+04	1.15E+05	7.02E+03	0.00E+00	1.01	0.81	—	—	0.17	0.25	—	—	16.31	0.17	—	—
P01911	HLA class II histocompatibility antigen, DRB1 beta chain	3.16E+04	0.00E+00	1.02E+05	1.71E+04	0.00E+00	—	—	—	—	0.31	0.13	—	—	5.97	0.20	—	—
P02655	Apolipoprotein C-II	1.92E+04	1.99E+04	2.29E+04	0.00E+00	0.00E+00	0.97	0.20	—	—	0.84	0.62	—	—	—	—	—	—
P05161	Ubiquitin-like protein ISG15	2.74E+04	2.92E+04	7.94E+04	0.00E+00	0.00E+00	0.94	0.36	—	—	0.35	0.47	—	—	—	—	—	—
P11216	Glycogen phosphorylase, brain form	1.98E+04	1.96E+04	7.84E+04	0.00E+00	0.00E+00	1.01	0.76	—	—	0.25	0.49	—	—	—	—	—	—
P12081	Histidine--tRNA ligase, cytoplasmic	1.91E+04	2.81E+04	1.34E+05	0.00E+00	0.00E+00	0.68	0.59	—	—	0.14	0.44	—	—	—	—	—	—

P13761	HLA class II histocompatibility antigen, DRB1 beta chain	3.44E+04	2.42E+04	1.46E+05	0.00E+00	0.00E+00	1.42	0.31	—	—	0.24	0.78	—	—	—	—	—	—
P17612	cAMP-dependent protein kinase catalytic subunit alpha	1.85E+04	0.00E+00	5.28E+04	2.31E+04	0.00E+00	—	—	—	—	0.35	0.50	—	—	2.28	0.82	—	—
P27816	Microtubule-associated protein 4	2.51E+04	7.01E+04	1.46E+05	7.91E+03	0.00E+00	0.36	0.40	—	—	0.17	0.62	—	—	18.43	0.92	—	—
P51170	Amiloride-sensitive sodium channel subunit gamma	4.71E+04	7.74E+04	6.94E+04	8.86E+04	0.00E+00	0.61	0.93	—	—	0.68	0.74	—	—	0.78	0.24	—	—
P51858	Hepatoma-derived growth factor	1.81E+04	2.25E+04	1.08E+05	0.00E+00	0.00E+00	0.80	0.56	—	—	0.17	0.05	—	—	—	—	—	—
P55327	Tumor protein D52	1.98E+04	1.33E+04	9.94E+04	1.62E+04	0.00E+00	1.49	0.53	—	—	0.20	0.54	—	—	6.13	0.80	—	—
Q01628	Interferon-induced transmembrane protein 3	6.08E+04	4.44E+04	1.50E+05	5.71E+04	0.00E+00	1.37	0.96	—	—	0.41	0.88	—	—	2.62	0.55	—	—
Q13261	Interleukin-15 receptor subunit alpha	1.15E+05	1.09E+05	3.16E+05	0.00E+00	0.00E+00	1.05	0.16	—	—	0.36	0.37	—	—	—	—	—	—
Q13938	Calcyphosin	2.49E+04	2.23E+04	9.47E+04	2.24E+04	0.00E+00	1.12	0.88	—	—	0.26	0.18	—	—	4.23	0.66	—	—
Q6UXC1	Apical endosomal glycoprotein	1.82E+06	1.90E+06	4.71E+06	2.60E+06	0.00E+00	0.96	0.80	—	—	0.39	0.77	—	—	1.81	0.98	—	—
Q92876	Kallikrein-6	5.80E+04	2.25E+04	5.94E+04	8.00E+04	0.00E+00	2.57	0.19	—	—	0.98	0.15	—	—	0.74	0.08	—	—
Q9NZJ5	Eukaryotic translation initiation factor 2-alpha kinase 3	1.08E+06	2.61E+06	2.10E+06	2.53E+06	0.00E+00	0.41	0.00	—	—	0.51	0.11	—	—	0.83	0.48	—	—
Q9P1F3	Costars family protein ABRACL	4.02E+04	1.39E+05	1.55E+05	8.37E+04	0.00E+00	0.29	0.25	—	—	0.26	0.33	—	—	1.85	0.53	—	—
Q9UNZ2	NSFL1 cofactor p47	4.04E+04	1.31E+04	4.24E+04	2.16E+04	0.00E+00	3.08	0.53	—	—	0.95	0.59	—	—	1.96	0.84	—	—

RMS: rhabdomyosarcoma. HC: healthy control. QC: quality control samples. RN0: newly diagnosed RMS at the time points of admission. RN1: newly diagnosed RMS at the time points after 4 cycles of chemotherapy. RS0: RMS undergone surgery at the time points of admission. RS1: RMS undergone surgery at the time points after 4 cycles of chemotherapy.
