# Supplementary material for: Quantitative proteomics identifies and validates urinary biomarkers of rhabdomyosarcoma in children
Source: Clin Proteomics. 2023 Mar 14;20:10. doi: 10.1186/s12014-023-09401-4 (PMC10012572; doi:10.1186/s12014-023-09401-4)
Supplement: Supplementary file 2 — Additional file 2: Table S2. Biological processes analysis of 251 differential proteins. [file 12014_2023_9401_MOESM2_ESM.pdf]

Table S2 Biological processes analysis of 251 differential proteins.

Category	Biological Processes	P Value	-log(P-Value)	Fold Enrichment	Number of proteins	UniProt accession
Tumor invasion	neutrophil degranulation	4.79E-14	13.32	5.37	32	P31997, P12318, Q10588, P19652, P10253, P19801, P20933, P20138, P61160, P04080, Q9HBH0, P23467, P22894, P02763, O75629, P07478, P01011, P28676, O43242, Q14314, Q9Y376, O75015, P13798, P11216, O75131, P17931, P15144, P08174, P01009, Q92542, Q6ZQN7, Q14165
Tumor invasion	platelet degranulation	4.82E-09	8.32	9.07	14	P01011, P02671, P07948, P19652, O43707, P21926, P49908, P01009, P02787, P09486, P05067, P02763, Q99969, P10909
Tumor invasion	angiogenesis	2.54E-04	3.59	3.93	12	Q07075, P15144, P39059, P04216, Q9Y4C0, Q9Y653, P35916, P23467, O14786, O75340, P35613, Q9NZJ5
Tumor invasion	cell adhesion	4.98E-04	3.30	2.64	18	O75309, P32942, P04216, Q9HBB8, Q9Y653, P19022, P13598, P35613, P16112, P20138, Q08629, P39059, P21926, P21583, Q14982, P98172, P05067, Q9UN74
Tumor invasion	positive regulation of endothelial cell migration	8.87E-03	2.05	6.13	5	P35916, O14786, P09486, O75340, P35613
Tumor invasion	endocytosis	1.37E-02	1.86	3.16	8	Q14677, P06127, Q9NZN3, Q96GP6, Q9P2R3, P05067, P62820, P60953
Tumor invasion	positive regulation of substrate adhesion-dependent cell spreading	1.58E-02	1.80	7.53	4	P02671, O15144, O14786, P60953
Tumor invasion	positive regulation of cell proliferation	1.59E-02	1.80	2.11	14	P04632, Q10588, P43490, P07948, P35916, P36897, P60033, Q99650, Q9BXN2, P21583, P01225, P08887, Q13277, P07333
Tumor invasion	adherens junction organization	1.89E-02	1.72	7.04	4	P15151, P19022, P12830, P60953
Inflammation	positive regulation of interleukin-6 production	4.70E-06	5.33	7.94	10	P18428, Q96A25, P22894, Q9BXN2, Q06124, P05067, P08887, P35613, P10809, P04233
Inflammation	positive regulation of tumor necrosis factor production	5.97E-06	5.22	7.71	10	P18428, Q96A25, P22894, Q9BXN2, Q06124, P19652, P05067, P02763, P10809, P10909
Inflammation	positive regulation of chemokine production	2.24E-03	2.65	9.00	5	P18428, P05067, P08887, P07333, P04233

Inflammation	cytokine-mediated signaling pathway	2.28E-03	2.64	3.01	12	P17612, P06734, P18428, Q99650, P04083, Q06124, P14778, P08887, Q13277, P07333, P14784, P31785
Inflammation	leukocyte migration	2.99E-03	2.52	3.72	9	P31997, P57087, P07948, P01624, Q06124, P35613, P04233, P01782, Q96AP7
Inflammation	positive regulation of interleukin-1 beta production	7.97E-03	2.10	6.32	5	Q96A25, Q9BXN2, P19652, P05067, P02763
Inflammation	response to interleukin-1	1.30E-02	1.89	8.10	4	P04083, P14778, P05067, P50135
Inflammation	positive regulation of interleukin-10 production	1.39E-02	1.86	7.90	4	P05161, Q01151, Q9BXN2, P10809
Inflammation	negative regulation of inflammatory response to antigenic stimulus	1.58E-02	1.80	3.07	8	P16444, P17612, P12318, P01911, P07948, P01624, P01225, P01782
Inflammation	positive regulation of interferon-gamma production	1.63E-02	1.79	5.12	5	P05161, Q9BXN2, P14778, P05067, P10809
Immunity	regulation of immune response	7.52E-05	4.12	4.52	12	P32942, P20138, P43626, P12318, P18627, P60033, P15151, P12319, P01624, O75015, P13598, P01782
Immunity	T cell costimulation	4.36E-03	2.36	7.50	5	P06127, P07948, Q06124, P98172, P60953
Immunity	immune response	6.39E-03	2.19	2.37	14	P31997, A0A075B6S5, O75015, P14778, P55899, P31785, P43626, A0A0C4DH24, P01911, P01624, A0A0B4J2D9, Q01628, P04233, P01782
Immunity	innate immune response	7.95E-03	2.10	2.14	16	P02671, A0A075B6Q5, Q96A25, P07948, P30530, A0A0C4DH33, P17931, P18428, P08174, P04083, Q9BXN2, P05067, P07333, Q99969, P01782, P10909
Neuronal damage	neuron projection development	8.27E-04	3.08	5.27	8	P27816, P02649, P07948, P12830, P49841, P05067, Q13277, Q15751
Neuronal damage	axonogenesis	3.60E-03	2.44	5.85	6	Q15831, Q9UQP3, Q9UBP0, Q06124, P05067, Q9H5Y7
Neuronal damage	response to calcium ion	6.73E-03	2.17	6.64	5	P02671, Q9UBV8, O43865, P09486, O75340
Neuronal damage	positive regulation of neuron death	1.48E-02	1.83	7.71	4	P02649, P49841, P05067, P10909
Neuronal damage	neuron apoptotic process	1.78E-02	1.75	7.20	4	O95831, P55210, Q92542, P05067
