# Supplementary material for: Quantitative proteomics identifies and validates urinary biomarkers of rhabdomyosarcoma in children
Source: Clin Proteomics. 2023 Mar 14;20:10. doi: 10.1186/s12014-023-09401-4 (PMC10012572; doi:10.1186/s12014-023-09401-4)
Supplement: Supplementary file 3 — Additional file 3: Table S3. Pathway analysis of 251 differential proteins. [file 12014_2023_9401_MOESM3_ESM.pdf]

Table S3 Pathway analysis of 251 differential proteins.

ID	Ingenuity Canonical Pathways	-log(p-value)	Ratio	Number of proteins	Proteins
1	Epithelial Adherens Junction Signaling	4.07	0.0581	9	ACTR2,ACVR2B,ARPC2,ARPC3,CDC42,CDH1,CDH2,STK11,TGFBR1
2	Fcγ Receptor-mediated Phagocytosis in Macrophages and Monocytes	3.97	0.0753	7	ACTR2,ARPC2,ARPC3,CDC42,FCGR2A,FCGR3A/FCGR3B,LYN
3	Integrin Signaling	3.83	0.0488	10	ACTN4,ACTR2,ARPC2,ARPC3,CAPNS1,CDC42,GSK3B,PPP1CB,RHOF,TSPAN6
4	SPINK1 Pancreatic Cancer Pathway	3.36	0.0909	5	CPZ,KLK4,KLK6,PRSS2,TGFBR1
5	Th1 and Th2 Activation Pathway	3.12	0.0479	8	ACVR2B,HLA-A,HLA-DRB1,IL2RB,IL2RG,IL6R,NCSTN,TGFBR1
6	RHOGDI Signaling	3.06	0.0425	9	ACTR2,ARHGAP1,ARPC2,ARPC3,CDC42,CDH1,CDH16,CDH2,RHOF
7	Th2 Pathway	3.03	0.0526	7	ACVR2B,HLA-A,HLA-DRB1,IL2RB,IL2RG,NCSTN,TGFBR1
8	STAT3 Pathway	2.99	0.0519	7	FLT4,IL15RA,IL1R1,IL2RB,IL2RG,IL6R,TGFBR1
9	PD-1, PD-L1 cancer immunotherapy pathway	2.89	0.0583	6	GSK3B,HLA-A,HLA-DRB1,IL2RB,IL2RG,PTPN11
10	NAD Signaling Pathway	2.81	0.0483	7	AIFM1,BST1,EIF2AK3,GSK3B,H1-5,HSPD1,NAMPT
11	Regulation of Actin-based Motility by Rho	2.74	0.0545	6	ACTR2,ARPC2,ARPC3,CDC42,PPP1CB,RHOF
12	Natural Killer Cell Signaling	2.72	0.0415	8	CDC42,FCGR2A,FCGR3A/FCGR3B,HLA-A,IL2RB,KIR2DL1/KIR2DL3,PTPN11,PVR
13	Role of JAK family kinases in IL-6-type Cytokine Signaling	2.53	0.1200	3	IL6R,OSMR,PTPN11
14	WNT/β-catenin Signaling	2.42	0.0412	7	ACVR2B,CDH1,CDH2,GSK3B,MMP7,SFRP4,TGFBR1
15	Colorectal Cancer Metastasis Signaling	2.42	0.0341	9	CDC42,CDH1,GSK3B,IL6R,MMP7,MMP8,PRKACA,RHOF,TGFBR1
16	B Cell Receptor Signaling	2.41	0.0300	11	CDC42,FCGR2A,GSK3B,IGHV1-24,IGHV3-64,IGKV1-27,IGKV1D-13,IGKV3-15,IGKV6-21,LYN,PTPN11
17	IL-12 Signaling and Production in Macrophages	2.32	0.0448	6	APOC2,APOE,CLU,ORM1,ORM2,SERPINA1

18	Role of JAK1 and JAK3 in γc Cytokine Signaling	2.12	0.0597	4	IL15RA,IL2RB,IL2RG,PTPN11
19	FAK Signaling	2.09	0.0218	18	ACTR2,ACVR2B,ADGRB2,ADGRG1,ARPC2,ARPC3,CAPNS1,CDH1,EFNB1,FLT4,GPR180,GSK3B,IL15RA,IL1R1,IL2RB,IL2RG,IL6R,TGFBR1
20	PI3K/AKT Signaling	2.08	0.0355	7	CDC37,GSK3B,IL15RA,IL1R1,IL2RB,IL2RG,IL6R
21	Antigen Presentation Pathway	2.02	0.0789	3	CD74,HLA-A,HLA-DRB1
22	Macropinocytosis Signaling	1.93	0.0526	4	ACTN4,ANKFY1,CDC42,CSF1R
23	Signaling by Rho Family GTPases	1.9	0.0302	8	ACTR2,ARPC2,ARPC3,CDC42,CDH1,CDH16,CDH2,RHOF
24	RHOA Signaling	1.85	0.0410	5	ACTR2,ARHGAP1,ARPC2,ARPC3,PPP1CB
25	Regulation Of The Epithelial Mesenchymal Transition In Development Pathway	1.76	0.0471	4	CDH1,GSK3B,LOX,NCSTN
26	IL-4 Signaling	1.68	0.0444	4	FCER2,HLA-A,HLA-DRB1,IL2RG
27	Actin Cytoskeleton Signaling	1.65	0.0292	7	ACTN4,ACTR2,ARPC2,ARPC3,CDC42,LBP,PPP1CB
28	TGF-β Signaling	1.59	0.0417	4	ACVR2B,CDC42,INHBC,TGFBR1
29	ID1 Signaling Pathway	1.54	0.0302	6	ACVR2B,APP,GSK3B,IL6R,LYN,TGFBR1
30	CDC42 Signaling	1.36	0.0237	8	ACTR2,ARPC2,ARPC3,CDC42,GSK3B,HLA-A,HLA-DRB1,PPP1CB
31	Sonic Hedgehog Signaling	1.35	0.0690	2	GSK3B,PRKACA
32	Th1 Pathway	1.32	0.0342	4	HLA-A,HLA-DRB1,IL6R,NCSTN
33	Sirtuin Signaling Pathway	1.32	0.0246	7	APP,CDH1,GOT2,GSK3B,H1-5,NAMPT,STK11
