# Supplementary material for: Quantitative proteomics identifies and validates urinary biomarkers of rhabdomyosarcoma in children
Source: Clin Proteomics. 2023 Mar 14;20:10. doi: 10.1186/s12014-023-09401-4 (PMC10012572; doi:10.1186/s12014-023-09401-4)
Supplement: Supplementary file 4 — Additional file 4: Table S4. Diseases annotation of 251 differential proteins. [file 12014_2023_9401_MOESM4_ESM.pdf]

Table S4 Diseases annotation of 251 differential proteins.

Categories	Diseases or Functions Annotation	P-value	-log10p-value	Number of proteins	Proteins
Cancer,Organismal Injury and Abnormalities	Advanced extracranial solid tumor	2.76E-06	5.56	26	ABAT,ANPEP,AXL,BSG,CD9,CDH2,CLU,COL15A1,CSF1R,EIF2AK3,EIF4A1,ENPEP,FBN2,FCGR2A,FCGR3A/FCGR3B,FLT4,IL2RB,IL2RG,KLK6,LAG3,LGALS3,LYN,MAP4,MMP7,TF,TGFBR1
Cancer,Organismal Injury and Abnormalities	Advanced malignant solid tumor	5.58E-05	4.25	27	ABAT,ANPEP,ANXA1,AXL,BSG,CD9,CDH2,CLU,COL15A1,CSF1R,EIF2AK3,EIF4A1,ENPEP,FBN2,FCGR2A,FCGR3A/FCGR3B,FLT4,IL2RB,IL2RG,KLK6,LAG3,LGALS3,LYN,MAP4,MMP7,TF,TGFBR1
Cancer,Organismal Injury and Abnormalities	Metastatic solid tumor	5.85E-05	4.23	24	ABAT,ANPEP,ANXA1,AXL,BSG,CD9,CDH2,CLU,CSF1R,EIF2AK3,EIF4A1,FBN2,FCGR2A,FCGR3A/FCGR3B,FLT4,IL2RB,IL2RG,LAG3,LGALS3,LYN,MAP4,MMP7,TF,TGFBR1
Cancer,Organismal Injury and Abnormalities	Primary solid tumor	7.85E-05	4.11	19	AOC1,AXL,CCT5,CLU,CSF1R,FCER2,FLT4,GOT2,IL2RB,IL2RG,ILF3,KLK6,LGALS3,MMP7,OSMR,SERPINA1,SLC9A3R1,SORD,TF
Cancer,Organismal Injury and Abnormalities	Stage IV solid tumor	1.42E-04	3.85	10	AXL,CSF1R,FCGR2A,FCGR3A/FCGR3B,FLT4,IL2RB,IL2RG,LAG3,LYN,TF
Cancer,Organismal Injury and Abnormalities	Unresectable solid tumor	2.98E-04	3.53	9	AXL,BSG,CLU,CSF1R,FLT4,IL2RB,IL2RG,LAG3,LYN
Cellular Movement	Cell movement of sarcoma cell lines	7.71E-05	4.11	9	ACTN4,ARPC2,CDC42,CDH1,CDH2,IL6R,KITLG,RARRES2,SLC9A3R1
Cellular Movement	Migration of sarcoma cell lines	1.41E-04	3.85	8	ACTN4,ARPC2,CDC42,CDH2,IL6R,KITLG,RARRES2,SLC9A3R1
Cellular Development,Cellular Growth and Proliferation,Organ Development,Skeletal and Muscular System Development and Function,Tissue Development	Proliferation of muscle cells	1.57E-04	3.80	9	APOE,FCER1A,FCER2,GSK3B,HSPD1,IL6R,MMP7,PTPN11,RARRES2
Cancer,Organismal Injury and Abnormalities,Skeletal and Muscular Disorders	Muscle tumor	3.87E-04	3.41	25	ADAMTSL3,AHCYL1,ANXA1,CDH1,COL15A1,CREG1,CSF1R,FCGR3A/FCGR3B,FLT4,HERC1,HNMT,ISOC1,KRT19,LYN,MAP4,MYO1B,NPEPPS,OGN,PLBD1,PTPN11,SERPINA1,STK11,STX3,SUPT5H,TSPAN6
