# Supplementary material for: Quantitative proteomics identifies and validates urinary biomarkers of rhabdomyosarcoma in children
Source: Clin Proteomics. 2023 Mar 14;20:10. doi: 10.1186/s12014-023-09401-4 (PMC10012572; doi:10.1186/s12014-023-09401-4)
Supplement: Supplementary file 5 — Additional file 5: Table S5. Tissue distribution analysis of 251 differential proteins. [file 12014_2023_9401_MOESM5_ESM.pdf]

Table S5 Tissue distribution analysis of 251 differential proteins.

UniProt accession	adipose tissue	adrenal gland	appendix	bone marrow	breast	bronchus	cartilage	caudate	cerebellum	cerebral cortex	cervix	colon	duodenum	endometrium	epididymis	esophagus	eye	fallopian tube	gallbladder	heart muscle	hippocampus	hypothalamus	kidney	liver
ABAT		Medium				High		High	High	Medium			Medium			Medium		Medium	Medium		Medium		High	High
ABRACL			Medium		Medium	Medium		Medium		Medium		High	Medium	Medium	High			Medium	Medium				Medium	
ACAN							High																	
ACTN4		Medium	Medium		Medium	Medium				Medium		High	Medium	Medium	Medium			Medium	Medium				Medium	High
ACTR2		Medium	Medium	Medium	Medium	Medium			Medium	Medium	Medium	Medium	Medium	Medium	Medium			Medium	Medium		Medium		Medium	Medium
ACVR2B		Medium				Medium																		
ADGRG1		Medium	Medium	High		High					Medium	High	High		Medium	Medium		Medium	Medium				Medium	Medium
ADH1C			Medium		Medium							Medium	Medium		Medium	Medium			Medium				Medium	High
AGA		High	Medium		Medium	Medium			Medium		Medium	Medium	High	Medium	High			Medium	High	Medium			High	Medium
AHCYL1													Medium	Medium				Medium						
AIF1L					Medium					Medium		Medium		Medium	Medium								High	
AIFM1		High	Medium		Medium	Medium					Medium		Medium	High	High			High	Medium	Medium				Medium
ALDH1L1	Medium	Medium						Medium	Medium	Medium	Medium												High	High
ALDOB													Medium										High	High
ANKFY1		Medium						Medium	Medium	Medium		Medium	Medium	Medium	Medium			Medium	Medium	Medium	High		Medium	Medium
ANPEP			High									High	High						High				Medium	High
ANXA1	Medium		Medium	High	Medium	High				Medium	Medium	Medium		Medium	Medium	High		High					High	
AOC1			Medium									High	Medium											
APEH		Medium	Medium			Medium			Medium			Medium	Medium	Medium	Medium	Medium		Medium	Medium				High	
APOE		High						High	Medium	Medium											Medium		Medium	Medium
APP								Medium	Medium	Medium			Medium								Medium			
ARHGAP1		High		Medium	Medium	High				Medium	Medium	Medium	High	Medium	Medium	High		High	High	Medium			Medium	Medium
ARPC2	Medium	Medium	Medium	High	Medium	Medium		Medium	Medium	Medium	Medium	High	High	Medium	High	High		High	High	Medium	High		Medium	
ARPC3		Medium	High	High	Medium	Medium		Medium	Medium	High		High	Medium	Medium	Medium			Medium			High			
AXL			Medium									Medium	Medium	Medium					Medium				Medium	
BHMT																							High	High
BROX	Medium	Medium	Medium			Medium		Medium					Medium	Medium		Medium		Medium	Medium				Medium	
BSG			Medium							Medium		High	High	Medium	High	Medium			Medium	Medium			Medium	
BST1													Medium			Medium								
BTN2A2	Medium	Medium	Medium		Medium							Medium	High		Medium				Medium	Medium			High	High
CAB39		Medium	Medium	Medium	Medium	High		Medium	Medium	Medium	Medium	Medium	Medium	Medium	High	Medium		Medium	Medium	Medium	Medium		Medium	
CAPNS1	Medium	High	Medium	High	High	High		High	Medium	Medium	High	Medium		Medium	High	Medium		High	Medium		High		High	High
CAPS						High								Medium				High						
CAPZB		Medium	High	Medium	Medium	Medium				Medium	Medium	Medium	Medium	Medium	High	Medium		Medium	High					Medium
CASP7			High			Medium			High		Medium	Medium	High		Medium	Medium		Medium	Medium					
CC2D1A		Medium	High		Medium	Medium		Medium	Medium	Medium	Medium	Medium	High	Medium		Medium		Medium	High	Medium	Medium		Medium	Medium
CCS		High	Medium		Medium	Medium			Medium		Medium	High	High	Medium	Medium	Medium		Medium	High	Medium			High	High
CCT5	Medium	Medium	Medium	Medium				Medium	Medium	Medium		Medium	Medium	Medium	Medium			High			Medium			
CD5			Medium	Medium																				

CD9		Medium	Medium			Medium		Medium	Medium	Medium	Medium	Medium		Medium	Medium	Medium		High					Medium	
CD27			Medium																					
CD33															Medium								Medium	
CD55		Medium							Medium	Medium													Medium	
CD74			High			Medium			High			High	Medium	Medium				Medium					Medium	
CD81			Medium							Medium				Medium										
CD83			Medium	Medium					Medium															
CDC37	Medium	Medium	Medium	Medium	Medium	Medium		Medium	Medium	Medium	Medium	Medium	Medium	Medium	Medium	Medium		Medium	Medium		Medium		Medium	
CDC42		Medium	Medium	Medium	Medium	Medium		Medium	Medium	Medium		Medium	Medium	Medium	Medium			Medium	Medium		Medium		Medium	
CDH1			Medium		Medium	Medium					Medium	High	Medium	High	High	High		High	High				Medium	Medium
CDH2		High							High	Medium		High	Medium		Medium			Medium		High				High
CDH16																							High	
CDHR5			High									High	Medium		Medium				High				Medium	
CHMP2A		High	High	Medium	Medium			Medium	Medium	Medium	Medium	High	High	Medium	High	Medium		High	High		Medium		Medium	Medium
CHMP4B		Medium	High		Medium	Medium		Medium	Medium	Medium		Medium	High	Medium		Medium		Medium	Medium	Medium	Medium		Medium	
CLINT1	Medium	High	Medium	Medium	Medium	High		Medium	Medium	Medium	Medium	High	High	Medium	High	Medium		Medium	High	Medium				Medium
CLU						High				Medium	High		High	Medium	High			Medium			Medium			
COL9A2							High										High						Medium	
COL9A3							High										High							
COL15A1										Medium		Medium								Medium			Medium	
CPNE3		Medium	Medium		Medium	Medium		Medium		Medium		Medium	High	Medium	Medium			Medium	Medium	Medium			Medium	Medium
CPNE8	High	High	Medium	Medium	Medium	Medium			Medium		Medium	Medium	Medium	Medium	Medium	Medium		Medium	Medium	High	Medium		Medium	Medium
CSF1R		Medium	Medium	Medium		Medium			Medium	Medium	Medium	Medium	Medium	Medium	Medium	Medium		Medium	Medium	Medium	Medium		Medium	
CSRP1			High		Medium				High	Medium	Medium	High	High	Medium	High	Medium			High				High	
CSTB																High								
CUTA	Medium	High	Medium		Medium	Medium		Medium	Medium	Medium	Medium	Medium	Medium	Medium	Medium				Medium	Medium				Medium
CYB5A		Medium			Medium	Medium							Medium						Medium				Medium	Medium
DPEP1												High	High										Medium	
EFNB1	Medium	Medium	Medium	High	High	High		Medium	Medium	Medium	Medium	Medium	High	Medium	High	High		High	High	Medium	Medium		Medium	Medium
EHD3	Medium		Medium							Medium		High											High	
EIF2AK3		Medium	Medium		High	High		Medium	Medium	Medium	Medium	Medium	Medium	Medium	High	High			Medium	Medium	Medium			
EIF4A1			Medium	High		Medium					Medium	High	Medium			Medium								
EIF6		High	Medium	Medium	Medium			Medium	Medium	Medium	Medium	Medium	High	Medium	Medium	High		High	High	Medium	Medium		High	Medium
ENPEP													High										High	Medium
EPDR1		High		Medium	High	Medium		Medium	High	High			High	Medium	Medium			Medium	High	High	Medium		Medium	Medium
EPS8L2		Medium	High		Medium	High		Medium		Medium	High	High	Medium	Medium	Medium	High		Medium	High		Medium		High	
ESAM		Medium	Medium	Medium	Medium	Medium			Medium	Medium	Medium		Medium	Medium		Medium		Medium	Medium	Medium			Medium	Medium
FABP1			High									High	Medium											High
FAM174A		Medium	Medium		Medium	High		Medium	Medium	Medium	Medium	Medium	Medium	Medium	High	Medium		High	Medium		High			
FAM234A		Medium	High	High	High	Medium		Medium	Medium	Medium	Medium	Medium	High	Medium	Medium	Medium		Medium	Medium		Medium		Medium	Medium
FBN2																								
FCER1A								Medium			Medium								Medium					Medium
FCER2			Medium	Medium																				
FCGR2A				Medium																				
FCGRT									Medium															

FGL2				Medium				Medium	Medium	Medium		Medium												
FLT4		High	High		Medium	High			Medium	Medium	Medium	Medium	Medium		Medium	Medium		Medium	Medium	High			Medium	
FRRS1		Medium	Medium		Medium	Medium		Medium	Medium	Medium		Medium	Medium	Medium	High					Medium	Medium		Medium	Medium
FSHB																								
GAA		High			High							High		High	High				High		High		Medium	High
GCA				High																			Medium	
GGACT																							Medium	
GH1																								
GINM1		High	Medium		Medium	Medium			Medium	Medium		Medium	High	Medium		Medium		Medium	High	High			High	
GOT2		Medium	Medium		Medium	Medium		Medium	High	Medium	Medium	Medium	Medium	Medium	High	Medium		Medium	Medium	High	High		Medium	High
GPR180	Medium	Medium	Medium	Medium		Medium		Medium	Medium	Medium	Medium	Medium	Medium	Medium	Medium				High		Medium		Medium	Medium
GSK3B			Medium		Medium	Medium		High	Medium	Medium		Medium	Medium					Medium	Medium		High		Medium	
H1-5		Medium	High	High	Medium	High		Medium	Medium	Medium	High	Medium	High	Medium	Medium	High		High	High	Medium	Medium			
HAL	Medium	Medium	Medium	Medium	Medium	Medium			Medium	Medium	Medium	Medium	Medium	Medium	Medium	Medium		Medium	Medium	Medium			Medium	High
HARS1		Medium	High	High	High	High		Medium	Medium	Medium	Medium	Medium	High	Medium	High	High		High	High		Medium		Medium	Medium
HDGF	Medium	High	High	High	High	High		Medium	Medium	Medium	High	High	High	Medium	High	High		High	High	High	Medium			Medium
HEG1				Medium								Medium	Medium	Medium	Medium			Medium						Medium
HERC1																								
HLA-DRB1			Medium	Medium														High						
HNMT	Medium	Medium	Medium	Medium	Medium	High		Medium	Medium	Medium	Medium	Medium	High	Medium	Medium	Medium		High	High		Medium		Medium	Medium
HPN	Medium	Medium	Medium	Medium	Medium	Medium		Medium	Medium	Medium	Medium	Medium	Medium	Medium	High			Medium	High	High	Medium			Medium
HSPD1		High	High	Medium		High						Medium	High	Medium	Medium	Medium		High	Medium				High	Medium
ICAM3			High	High																				
IFITM3		Medium		High	Medium	Medium				Medium		Medium		Medium	High	Medium		High					High	
IL2RB				Medium																				
IL2RG												High												
IL15RA	Medium	Medium	Medium	Medium	Medium	Medium		Medium	Medium	Medium		Medium	High	Medium	Medium			Medium	High	Medium			Medium	
ILF3	High	High	High	High				High	High	High	High	High	High	High	High	High		High	High	High	High		High	Medium
KHK													Medium											High
KITLG				Medium																				
KLK4																								
KRT19			High		High	High					High	High	High	High	High	Medium		High	High				Medium	High
LGALS3	Medium		High		Medium	Medium			Medium	Medium	Medium	Medium	Medium	Medium	Medium			Medium	Medium	Medium			High	
LRFN3		Medium	High			Medium		Medium	Medium	Medium		Medium	Medium					Medium	Medium	Medium			Medium	Medium
LYN			Medium	Medium									Medium						Medium					
LYPD6																								
MAP4			Medium		Medium	Medium		High	High	High	High	Medium	Medium	Medium	High	Medium		Medium	Medium	Medium	High		High	Medium
MMP7																			Medium				Medium	
MMP8				High																				
MYO1B			Medium		Medium	Medium				Medium	Medium	Medium	Medium		Medium	Medium			Medium				Medium	Medium
NAMPT		Medium	Medium		Medium					Medium	Medium	Medium	Medium		Medium				Medium					
NCSTN		Medium	Medium			Medium			Medium	Medium	Medium	Medium	Medium	Medium	Medium	Medium		Medium	Medium		Medium		Medium	Medium
NPEPPS			Medium			Medium				Medium		Medium	Medium	Medium		Medium		Medium	Medium	Medium			Medium	Medium
NPTXR										Medium														
NRP1		Medium	Medium	Medium	Medium	High						Medium	Medium	Medium		Medium		High	Medium					Medium

NRXN3										Medium														
NSFL1C		Medium			Medium	Medium			Medium	Medium	Medium	Medium	Medium	Medium	Medium	High		Medium	Medium				Medium	Medium
NUDT3				High							High					Medium							Medium	Medium
NUDT5																								Medium
ORM1																								
OSMR	Medium	Medium			Medium	Medium		Medium	Medium	High	Medium	Medium	Medium	Medium	Medium	Medium		Medium	Medium		Medium		Medium	Medium
PCDHA4									Medium	Medium									Medium		Medium			
PDLIM2			Medium									Medium	High			Medium			High				High	
PEBP1		High			Medium			Medium	Medium	Medium		Medium	Medium		Medium			Medium	Medium	Medium	Medium		Medium	Medium
PGLS	High	High	Medium	High	Medium	High				Medium	Medium	Medium	High	Medium	Medium	Medium		High	High	Medium	Medium		High	Medium
PLBD1				Medium																				
PLEKHB2			High			Medium		Medium	Medium	Medium	High	Medium	High	High	High			High	High					
PPP1CB			Medium		Medium	Medium			Medium	Medium	Medium	Medium	Medium	Medium	Medium	Medium		Medium	High				Medium	
PRKACA	Medium	Medium	Medium			Medium		Medium	Medium	High	Medium	Medium		Medium	Medium			Medium		Medium	Medium			
PRSS2													High											
PSMB3	Medium	Medium	Medium	Medium	Medium	Medium		Medium	Medium	Medium	Medium	Medium	Medium	Medium	Medium	Medium		Medium	Medium				Medium	Medium
PSMD3		High	Medium	High	Medium	Medium		Medium	High	Medium	Medium	Medium	Medium	Medium	High	High		Medium	High	High	High		Medium	Medium
PTGDS	High	Medium	Medium	Medium	Medium	Medium			Medium	High		Medium	Medium	Medium	High	Medium		Medium	Medium		Medium		Medium	
PTPN11	High		Medium		Medium	Medium		High	High	Medium	Medium	Medium	Medium	High	High	Medium		High	High		High		Medium	
PTPRB			Medium		Medium	Medium				Medium					High			High	Medium	Medium	Medium			
PVR		Medium	Medium					Medium		Medium		Medium							Medium	High			Medium	
PYGB			Medium		Medium			Medium	Medium	Medium									Medium	Medium				
RAB1A		Medium	Medium		Medium	Medium			Medium	Medium		Medium	High	Medium	High			Medium	Medium	Medium			Medium	
RHOF			Medium		Medium					Medium		Medium		Medium	Medium									
RNF150		High	Medium		Medium	Medium				Medium	Medium	Medium	High	Medium	Medium	Medium		High	High	Medium			High	Medium
RNF167		Medium	Medium					Medium		Medium		Medium		Medium	Medium			Medium	Medium		Medium		Medium	
RPL4		Medium	Medium			Medium		Medium	Medium	Medium	Medium	Medium	Medium			Medium		Medium	Medium		Medium			
SCARF2		Medium	Medium	Medium	Medium	Medium		Medium	Medium	Medium	High	Medium	High	Medium	High	Medium		Medium	Medium		Medium		Medium	Medium
SCPEP1		High			Medium					Medium		Medium		Medium	High			Medium	High	Medium				High
SDF4		Medium	High			Medium		Medium	Medium	Medium	Medium	High	High	Medium					High				High	Medium
SELENOP		High	Medium		Medium	High		Medium	Medium	Medium	Medium	Medium	Medium	Medium	High			Medium	Medium	Medium	Medium		High	Medium
SERPINA1													Medium										Medium	
SERPINA3					Medium				High		High	Medium											Medium	
SERPINB13											Medium					Medium								
SFRP4														Medium										
SH3BGRL		Medium	Medium	Medium				Medium		Medium				Medium				Medium						
SHISA5	Medium	High	Medium		Medium			Medium	Medium	Medium	Medium	Medium	Medium	Medium		Medium		Medium	High	High	Medium		High	Medium
SLC9A3R1		Medium	Medium		Medium	Medium					Medium	High	High	High	High	Medium		High	High				High	Medium
SLC10A3		Medium	Medium	Medium	Medium	Medium			Medium	Medium	Medium	Medium	High	Medium	Medium	Medium		Medium		Medium			High	Medium
SLC39A14		Medium	High								Medium	High	Medium	Medium		High				Medium			High	Medium
SLITRK6	High	Medium	Medium	Medium	Medium	High		Medium		Medium	Medium	Medium	Medium	Medium	Medium	High		Medium	High		Medium		Medium	High
SORD			Medium								High			High				High	Medium				Medium	High
SPARC		High		Medium				Medium	High	Medium	Medium	Medium		Medium							Medium		High	
SPAST	Medium	Medium	Medium	Medium	Medium	Medium		Medium	Medium	Medium	Medium	High	High	Medium	Medium	Medium		Medium	Medium	High			Medium	Medium
SPESP1																								

SPINT1	High		High		High	Medium		Medium		Medium	Medium	Medium	High	Medium	Medium	Medium		Medium	High	Medium	Medium		Medium	Medium
SPINT2			Medium	Medium	Medium	Medium		Medium	Medium	Medium	Medium	Medium	Medium	Medium	Medium			Medium	Medium		Medium		Medium	
SPOCK2		Medium	Medium			Medium		High	Medium	Medium			Medium						Medium		Medium		Medium	
ST6GAL1			High						High			Medium											High	Medium
STAM2		High	High	High	Medium	High		Medium	Medium	Medium	Medium	Medium	High	Medium	Medium	High		High	High	Medium			Medium	Medium
STK11		Medium	Medium	Medium	Medium	Medium		Medium	Medium	Medium	Medium	Medium	Medium		Medium	Medium		Medium	Medium	Medium	Medium		Medium	Medium
STX3		Medium	Medium	Medium	Medium							Medium	Medium	Medium	Medium			High	Medium				High	Medium
SUPT5H	Medium	Medium	Medium	Medium	Medium	Medium			Medium	Medium	Medium	Medium	Medium	Medium	Medium	Medium		Medium	Medium	Medium			Medium	
TBC1D10A		High	High	Medium	Medium	High		Medium	Medium	Medium	Medium	Medium	High	High	Medium	High		Medium	High	High	High		High	Medium
TF													Medium											
TGFBR1			Medium			Medium			Medium	Medium	Medium	Medium	Medium					Medium	Medium				Medium	
THY1								Medium		Medium											Medium		Medium	
TMED3			Medium		Medium	Medium					Medium	High		High	High	High		Medium	Medium					
TMEM132A			Medium			High			Medium	Medium		Medium						High						
TPD52	Medium		Medium	Medium	Medium	Medium				Medium		Medium	Medium	High	High	Medium		High	High		High		Medium	Medium
TPPP3						Medium					Medium	Medium	Medium	Medium	Medium			Medium				Medium	High	
TSPAN6			Medium		High	High				Medium	High	Medium		High	Medium	High		High	Medium				Medium	Medium
UPB1																							Medium	Medium
VPS4A		Medium	Medium		Medium	Medium		Medium	Medium	Medium	Medium	Medium	Medium	Medium	Medium	Medium		Medium	Medium		Medium		Medium	
VTA1		Medium	Medium			High						Medium	Medium		Medium	Medium		Medium	Medium					Medium

lung	lymph node	nasopharynx	oral mucosa	ovary	pancreas	parathyroid gland	pituitary gland	placenta	prostate	rectum	salivary gland	seminal vesicle	skeletal muscle	skin	small intestine	smooth muscle	soft tissue	spleen	stomach	testis	thyroid gland	tonsil	urinary bladder	vagina
		High			Medium	Medium			Medium			Medium			Medium		Medium		Medium	High	Medium			
Medium	Medium							High		Medium		Medium		Medium	Medium			Medium		High	High	Medium	Medium	
																	High							
Medium					Medium			Medium		Medium	Medium	Medium		Medium	Medium	Medium		Medium	Medium	Medium	Medium	Medium	High	
Medium	High	Medium			Medium	Medium		Medium	Medium	Medium		Medium		Medium	Medium		Medium	Medium	Medium	Medium	Medium	Medium	Medium	
		Medium				Medium		Medium									High			High	Medium			
	Medium	Medium	Medium		Medium			Medium	Medium	High	Medium	Medium		Medium	High				Medium	Medium	Medium	Medium	Medium	
			Medium							Medium		Medium			Medium				Medium				Medium	
Medium		Medium		Medium	Medium	High		Medium	High	Medium	Medium	High			High		Medium		Medium	High	Medium	Medium		
					Medium																Medium			
										High		Medium		Medium						Medium				
Medium					Medium	High			Medium	Medium	Medium	High			Medium			High	High	High		Medium	Medium	
				Medium						Medium	Medium									High			Medium	
															High									
Medium		Medium						Medium							Medium					High				
					High			Medium	Medium					Medium	High									
Medium	Medium	High	High					High	High		Medium	Medium		Medium			Medium	High				Medium	Medium	Medium
								Medium	Medium	Medium					Medium					Medium				
		Medium		Medium	Medium	Medium		Medium	Medium	Medium	Medium	Medium		Medium	Medium			Medium	Medium		Medium	Medium	Medium	
								Medium						Medium										
										Medium										Medium				
Medium	Medium	Medium	Medium	Medium	Medium			High	Medium	High	Medium			Medium	Medium	High	Medium	Medium	High	Medium	High	Medium	High	Medium
Medium	High	Medium	High	Medium	Medium	Medium		High	Medium	High	Medium	High		Medium	High	Medium	Medium	High	High	High	Medium	High	High	Medium
High	High	Medium						High		Medium					Medium		Medium	Medium	High	Medium	Medium	Medium	High	
Medium										Medium			High	Medium	Medium		Medium			Medium				
		Medium	Medium		Medium			Medium		Medium				Medium	Medium		Medium		Medium	Medium	Medium		Medium	
			Medium					Medium	Medium	Medium				Medium	High				High	High				
			Medium											Medium	Medium	Medium		Medium		Medium		Medium		Medium
						Medium				Medium					High	Medium			High					
Medium	Medium	High		High	Medium			Medium	Medium	Medium	High	Medium	High	Medium	Medium	Medium	Medium	Medium	Medium	Medium	High	Medium	Medium	Medium
Medium	Medium	High	Medium		High	High		Medium	Medium	Medium	High	High		Medium		Medium	Medium	Medium	High	Medium	High	Medium	High	Medium
		Medium																						
Medium	High	Medium	Medium	Medium	Medium	Medium		High	Medium	Medium	Medium	Medium		Medium	Medium		Medium	Medium	Medium	Medium	Medium	Medium	High	
Medium		Medium		Medium	Medium	Medium		Medium	Medium	High	Medium	Medium		Medium	Medium				High	Medium		Medium	Medium	
Medium	Medium	Medium	Medium	Medium				Medium		Medium	Medium	Medium	Medium	Medium	High				Medium	Medium	Medium	Medium	Medium	Medium
Medium	Medium	Medium		High		Medium			Medium	Medium	High	High	Medium	Medium	High	Medium	Medium	Medium	Medium	Medium	High	Medium	Medium	
Medium	High	Medium			Medium	High		Medium		Medium							Medium		Medium	Medium	Medium	Medium	Medium	
	Medium																	Medium		Medium		Medium		

High		High	High	Medium	Medium	High			High	High	High	High		Medium	High	Medium	Medium	Medium	Medium	Medium	Medium		High	High
	Medium																	Medium				Medium		
	Medium										Medium							Medium		Medium		Medium		
Medium				High				High			Medium			Medium						High				
Medium	Medium							Medium		High	Medium			Medium	High		Medium	Medium				Medium	Medium	
	Medium			High				High						Medium		Medium	Medium			Medium		High		
Medium	Medium																	Medium		Medium		Medium		
Medium	Medium	Medium	Medium	Medium		Medium		Medium	Medium	Medium	Medium			Medium	Medium			Medium	Medium	Medium	Medium	Medium	Medium	
	Medium				Medium			Medium		Medium	Medium	Medium	Medium		Medium			Medium	Medium	Medium	Medium	Medium	Medium	
		Medium	High		High	High		High	High	High	High	High		Medium	Medium				High		High	High	High	High
					Medium					High									Medium	High				
																					High			
										High					High									
Medium		Medium	Medium	Medium	Medium	High		Medium	Medium	High	Medium	High		Medium	High		Medium		Medium	Medium	High	Medium	High	
	Medium	Medium			Medium			Medium	Medium	Medium				Medium	High			Medium	Medium	Medium	Medium			
Medium	Medium	High		Medium	Medium	High		Medium	Medium	High	High	High		Medium	High		Medium	Medium	High	Medium	Medium	Medium	High	Medium
					Medium										High				Medium	Medium		High		
														Medium										
														Medium			Medium							
Medium			High	Medium	Medium	Medium		Medium	Medium	Medium	Medium	Medium	Medium	Medium	High	Medium	Medium	Medium	Medium	Medium	High		Medium	
Medium		High	Medium		Medium	Medium		Medium	Medium	Medium	High	High	Medium	Medium	Medium	Medium	Medium		Medium	Medium	Medium		Medium	Medium
Medium	Medium	Medium	Medium			Medium		Medium		Medium		Medium	Medium		Medium			Medium	Medium	Medium	Medium	Medium	Medium	
	Medium				Medium	Medium			Medium	High	High				High			Medium	Medium		Medium	Medium		Medium
			Medium																			Medium	Medium	Medium
High		Medium		Medium	Medium	Medium		Medium	Medium	Medium	Medium	Medium		Medium	Medium		Medium	Medium	Medium	Medium	High			Medium
High												Medium			Medium					High			Medium	
					High										High					Medium				
Medium	Medium	High	Medium	Medium	Medium	High		Medium	Medium	High	Medium	Medium	High	Medium	Medium		Medium		High	Medium	High	Medium	High	Medium
Medium	Medium			Medium				Medium								Medium				Medium		Medium		
Medium		Medium	Medium		Medium	Medium		Medium	Medium	Medium	Medium	High	Medium	Medium	High	Medium	Medium	Medium	Medium	Medium	High	Medium	Medium	Medium
	Medium	Medium	Medium		Medium			High		High	Medium				Medium			Medium	High	Medium		Medium	Medium	Medium
Medium	Medium	Medium	Medium	Medium	Medium	Medium		Medium	Medium	High	Medium			Medium	High		Medium	Medium	High	Medium	Medium	Medium	Medium	High
						High									High									
				Medium	Medium				High	Medium	Medium		Medium		Medium	Medium	Medium	Medium		Medium	High	Medium		
Medium		Medium	Medium		Medium			High	Medium	High	High	Medium		High	High	Medium			Medium		High	High	High	High
Medium	Medium	Medium	Medium	Medium	Medium	Medium		Medium	Medium	Medium	Medium	Medium		Medium	Medium	Medium		Medium	Medium	Medium	Medium	Medium	Medium	
										High					Medium									
		High	Medium		Medium	Medium		Medium	High	Medium	Medium	High			Medium		Medium		High	Medium	Medium	Medium	Medium	
High	Medium		Medium	High	Medium	High		High	Medium	High		Medium		Medium	High		Medium	Medium	Medium	Medium	High	Medium	Medium	Medium
								Medium																
Medium	Medium														Medium				Medium	Medium		Medium		
	Medium																	Medium				Medium		
High																		Medium				Medium		

	Medium							Medium						High				High						
Medium		High							Medium	High	Medium	Medium	High	Medium	High				Medium	Medium		Medium	Medium	
Medium	Medium	Medium				Medium		Medium						Medium	Medium		Medium		Medium	Medium	Medium	Medium		
							High																	
Medium					Medium	High		Medium	High		Medium	High		High					Medium	Medium	High			
Medium														Medium				High						
							High	High																
Medium		Medium			Medium	High		Medium		Medium	Medium	Medium	Medium	Medium	High				High	Medium	Medium	Medium	Medium	
	Medium	Medium		High	Medium	Medium			Medium	Medium		High		Medium	Medium			Medium	Medium	Medium	Medium	Medium	High	
High				Medium	Medium			Medium	Medium	Medium		Medium					Medium	High	Medium	Medium		Medium		
	Medium		Medium			Medium		Medium		Medium	Medium			Medium	Medium				Medium	Medium		Medium	Medium	
High	High	High	High	Medium	Medium	Medium		Medium		High	Medium	Medium	Medium	Medium	High		Medium	High	High	Medium	Medium	High	High	High
Medium	Medium	Medium	Medium		Medium	Medium		Medium	Medium	Medium	Medium	Medium	Medium	Medium	Medium	Medium	Medium	Medium	Medium	Medium	Medium	Medium	Medium	Medium
Medium	High	High	High	Medium	Medium	High		Medium	High	High	High	High		Medium	High		Medium	Medium	High	Medium	Medium	High	High	High
Medium	Medium	High	High	Medium	High	Medium		High	High	High	High	High	High	High	High	High	Medium	Medium	High	Medium	High	Medium	High	High
	Medium			Medium	Medium			Medium	High	Medium					Medium				Medium	Medium	High	Medium		
	Medium																	Medium						
Medium	High	Medium																				High		
Medium	Medium	Medium	Medium	Medium	Medium	Medium		Medium	Medium	Medium	Medium	Medium		Medium	Medium	Medium	Medium		Medium	Medium	High	Medium	Medium	Medium
Medium		Medium			Medium	Medium		Medium	Medium	High		High		Medium	Medium		Medium		Medium	Medium	High	Medium	Medium	Medium
Medium					Medium	Medium		Medium	Medium	High				Medium	Medium	Medium			Medium	Medium		Medium		
	High													Medium				High				High		
Medium		Medium		Medium		High							Medium	Medium		High	Medium		High	High	High	Medium		
	Medium																	Medium				Medium		
										High								Medium						
Medium	Medium	Medium	Medium	Medium	Medium			Medium	Medium	High	High	Medium	Medium	Medium	High		Medium		High	Medium	Medium	Medium	High	
High	High	High	High	High	High	High		High	High	High	High	High	High	Medium	High	High	High	High	High	High	High	High	High	High
															Medium									
									High															
High		High			High	Medium		High	High	High	High	High		Medium	High				High		High	High	High	Medium
High								Medium	Medium	Medium		Medium	Medium	Medium	Medium	Medium	Medium		Medium	Medium	Medium	Medium	High	Medium
		Medium			Medium	Medium		Medium		Medium	Medium		High	Medium	Medium				Medium	Medium				
Medium	High							High										High				High		
																				Medium				
Medium	Medium			Medium	Medium			Medium		Medium		Medium	Medium	Medium	High	Medium	High	Medium		Medium		Medium	High	Medium
																		High						
Medium	Medium		Medium	Medium	Medium					Medium		Medium		Medium	Medium			Medium	Medium	Medium		Medium	Medium	
					Medium					Medium					Medium		Medium	Medium	Medium	Medium		Medium		
High		Medium		Medium	Medium				Medium	Medium		Medium		Medium	Medium				Medium	Medium		Medium		
		Medium			Medium	Medium				Medium			Medium		Medium				Medium	Medium	Medium		Medium	
	Medium	High			Medium	Medium		Medium		Medium					Medium	Medium		Medium	Medium	Medium				

	Medium	Medium	Medium	Medium	Medium	Medium		Medium	High	Medium	Medium	Medium		Medium	Medium	Medium		Medium	Medium	Medium	Medium	Medium	Medium	High
	Medium		Medium										Medium	High				Medium		Medium	Medium	Medium		High
																				Medium				
																				Medium				
Medium		Medium	Medium					Medium		Medium		Medium	High	Medium	Medium		Medium			Medium	Medium	Medium	Medium	Medium
										Medium					Medium									
Medium			Medium		Medium			High					Medium	Medium	High			Medium				Medium		
Medium				Medium	Medium	High			Medium	Medium	Medium			Medium	Medium		Medium			Medium	High			
Medium	Medium	High	Medium	Medium	High	Medium		Medium	Medium	High	Medium	High	High	Medium	Medium	Medium	Medium	High	Medium	Medium	High	Medium		Medium
High	Medium																	Medium				Medium		
High					High			Medium	High	High	Medium	High		High	High				Medium	Medium	High	Medium		
Medium		Medium	Medium	Medium	Medium			Medium		Medium	Medium	Medium		Medium	Medium	Medium	Medium		Medium	Medium	Medium		Medium	
Medium	Medium	Medium	Medium	Medium	Medium	Medium		Medium	Medium		Medium	Medium	Medium	Medium			High	Medium	Medium	Medium	Medium	Medium	Medium	
					High										High									
Medium		Medium	Medium	Medium	Medium	Medium		Medium		Medium	Medium	Medium		Medium	Medium	High	Medium		Medium	Medium	Medium	Medium	Medium	Medium
Medium	Medium	High	Medium	Medium	Medium	High		High	Medium	High				Medium	High				High	High	Medium	Medium	Medium	Medium
Medium		Medium	Medium		Medium			Medium	Medium					Medium	Medium		Medium		Medium	Medium		Medium	Medium	Medium
Medium	High	Medium	Medium	High	Medium	Medium		High	Medium	High	High			Medium	Medium		Medium	Medium	High	Medium	High	High	High	Medium
	Medium				Medium				High			Medium			Medium		Medium	Medium	High	High		Medium		
					Medium	Medium		Medium	Medium	Medium		Medium							Medium	Medium	Medium	Medium		
					Medium				High	Medium	Medium								Medium	Medium				
	Medium	Medium		High	Medium			Medium	Medium	Medium	High	Medium		Medium	High		Medium		Medium	Medium	Medium	Medium		
Medium	Medium																	Medium	High	Medium		High		
Medium		Medium	Medium		Medium	High		Medium	High	High	Medium	Medium		Medium	High		Medium		High	Medium	Medium	Medium	Medium	Medium
								Medium		Medium		Medium		Medium		Medium				Medium	Medium	Medium		Medium
		Medium	Medium		Medium					Medium				Medium					Medium	Medium		Medium		Medium
Medium		Medium	Medium		Medium			High	High	High	Medium			Medium	High		Medium		Medium	Medium	Medium	Medium	Medium	Medium
High					Medium	High			High		High	High		Medium	High				High	High	High	High	High	
		Medium			Medium	Medium		High	High	High					High					High		Medium		
Medium		High	Medium	Medium	Medium	High		Medium	High	Medium	High	Medium	Medium	Medium	Medium		Medium	Medium	High	Medium	High	Medium	Medium	
High										Medium					Medium									
								Medium	Medium	Medium														
														Medium								Medium		High
																				Medium				
Medium	Medium										Medium	Medium				Medium	Medium	High		Medium	Medium	Medium	Medium	
Medium		High	High	Medium	Medium	Medium		Medium	Medium	Medium	Medium	Medium	High	Medium	Medium	Medium	Medium		Medium	Medium	Medium	Medium	Medium	Medium
Medium		High	Medium		Medium			High		Medium	Medium	High			High			Medium	High	Medium				
Medium	Medium	Medium	Medium	Medium	Medium	Medium		Medium	High	High	Medium	High	Medium	Medium	High	Medium	Medium	Medium	High	High	High	Medium	High	Medium
					Medium	Medium				High			Medium		High				Medium					High
High		High	Medium		Medium			High	Medium	Medium	Medium	Medium	Medium	Medium	Medium	Medium	Medium		Medium	Medium	High	Medium	Medium	High
		Medium							High			High			Medium				Medium		High		Medium	
Medium						Medium		Medium						Medium			Medium	Medium		High				
Medium	High	High	Medium	Medium	Medium	Medium		Medium		High	Medium	Medium	High	Medium	High	Medium	Medium	High	Medium	Medium	Medium	Medium	Medium	Medium
																				High				

Medium		High	Medium	Medium	Medium	Medium		High	High	High		Medium	Medium	Medium	High	Medium	Medium		High	Medium	Medium	Medium	High	
Medium	Medium	Medium		Medium	Medium	Medium		High	Medium	Medium	Medium	High		Medium	Medium				Medium	Medium	Medium	Medium	Medium	
Medium	Medium	Medium	Medium											Medium	Medium			Medium	Medium	Medium		Medium	Medium	
High	Medium								High	Medium								Medium	Medium	Medium	Medium	Medium		
Medium	High	High	Medium	Medium	Medium	Medium		High	Medium	High	High	Medium	High	Medium	High	High	Medium	Medium	High	Medium	High	High	High	Medium
Medium		Medium	Medium	Medium	Medium	Medium		Medium	Medium	Medium	Medium	Medium	Medium	Medium	Medium		High	Medium	Medium	Medium	Medium	Medium	Medium	Medium
	Medium	High			Medium			Medium	Medium	Medium	Medium	High			Medium					Medium		Medium	Medium	
Medium	Medium	Medium	Medium		Medium			Medium	Medium	Medium	Medium	Medium	Medium	Medium	Medium				Medium	Medium	Medium	Medium	Medium	Medium
Medium	Medium	High	Medium		Medium	High		High	Medium	High	Medium	High	Medium	Medium	High	Medium		Medium	High	Medium	High	Medium	Medium	Medium
								Medium							Medium									
		Medium								Medium		Medium			Medium		Medium		Medium					
														Medium										
	Medium		High		High			Medium		High	High	Medium		Medium	Medium				High	Medium		High	High	Medium
		High								Medium	Medium		Medium		Medium									
High	Medium	High		High	High	Medium		Medium	High	Medium	High	Medium		Medium	Medium			High	High	Medium	High	Medium	High	
High		Medium			Medium					High		Medium		Medium	High		Medium		Medium					
		High	Medium		Medium	Medium		High	Medium	Medium	Medium	High		Medium	Medium				Medium		Medium	High	High	High
Medium	Medium	Medium	Medium	Medium	Medium	Medium			Medium	Medium	Medium	Medium		Medium	Medium		Medium	Medium	Medium	Medium	Medium	Medium	Medium	Medium
		Medium		High	Medium					Medium		Medium			Medium		Medium		High	Medium	Medium	Medium	Medium	
