# Supplementary material for: Quantitative proteomics identifies and validates urinary biomarkers of rhabdomyosarcoma in children
Source: Clin Proteomics. 2023 Mar 14;20:10. doi: 10.1186/s12014-023-09401-4 (PMC10012572; doi:10.1186/s12014-023-09401-4)
Supplement: Supplementary file 6 — Additional file 6: Table S6. Distribution of differential proteins in each comparison based on PRM data. [file 12014_2023_9401_MOESM6_ESM.pdf]

Table S6 Distribution of differential proteins in each comparison based on PRM data.

Peptide name	UniProt accession	Protein name	RN0	RN1	RS0	RS1	HC	RN0 vs RN1		RN1 vs HC		RN0 vs RS0		RS0 vs HC		RS0 vs RS1		RS1 vs HC	
								**Fold Change**	***P***	**Fold Change**	***P***	**Fold Change**	***P***	**Fold Change**	***P***	**Fold Change**	***P***	**Fold Change**	***P***
FLENEDR	P01009	Alpha-1-antitrypsin	5.69E+08	5.88E+08	4.12E+08	2.06E+08	3.22E+08	0.97	0.89	1.83	0.02	1.38	0.51	1.28	0.58	2.00	0.44	0.64	0.42
AVLTIDEK	P01009	Alpha-1-antitrypsin	6.27E+08	5.44E+08	3.60E+08	6.84E+08	2.97E+08	1.15	0.53	1.83	0.02	1.74	0.29	1.21	0.69	0.53	0.31	2.30	0.04
SPLFMGK	P01009	Alpha-1-antitrypsin	2.89E+08	3.07E+08	2.13E+08	1.98E+08	1.25E+08	0.94	0.80	2.47	0.00	1.36	0.57	1.71	0.21	1.08	0.92	1.59	0.27
VELEDWAGNEAYAEYH[illegible]	P02671	Fibrinogen alpha chain	6.31E+07	5.28E+07	7.41E+07	1.84E+07	3.98E+07	1.20	0.75	1.33	0.39	0.85	0.86	1.86	0.20	4.02	0.08	0.46	0.41
NNSPYEIENGVVWVSFR	P02671	Fibrinogen alpha chain	3.04E+07	4.78E+07	1.19E+08	1.06E+07	1.23E+07	0.64	0.26	3.90	0.04	0.25	0.00	9.74	0.00	11.31	0.12	0.86	0.77
VIEIMQKPR	P09237	Matrilysin	2.29E+06	2.74E+06	1.30E+06	3.72E+06	1.65E+05	0.84	0.79	16.60	0.00	1.76	0.75	7.88	0.09	0.35	0.41	22.59	0.00
EIPLHFR	P09237	Matrilysin	1.31E+07	1.32E+07	1.38E+07	4.35E+06	5.73E+06	1.00	1.00	2.30	0.05	0.96	0.95	2.40	0.10	3.16	0.28	0.76	0.74
GFIPNIR	P35556	Fibrillin-2	1.42E+06	1.61E+06	7.31E+05	3.90E+05	6.93E+05	0.88	0.83	2.32	0.24	1.94	0.64	1.05	0.95	1.87	0.62	0.56	0.62
IPLLIFDQDEK	P51170	Amiloride-sensitive sodium channel subunit gamma	9.12E+06	6.22E+06	6.11E+06	5.11E+06	9.42E+06	1.47	0.38	0.66	0.24	1.49	0.64	0.65	0.53	1.20	0.85	0.54	0.43
SLDADEFR	Q13938	Calcyphosin	2.68E+06	2.88E+06	4.83E+05	1.66E+06	1.47E+06	0.93	0.77	1.96	0.03	5.54	0.07	0.33	0.17	0.29	0.28	1.13	0.83
SGDGVVTVDDLR	Q13938	Calcyphosin	4.16E+06	7.16E+06	3.50E+06	5.69E+06	2.61E+06	0.58	0.02	2.74	0.00	1.19	0.71	1.34	0.56	0.62	0.32	2.18	0.05
SGEWTEDEVLR	Q13938	Calcyphosin	1.84E+06	2.08E+06	8.20E+04	1.00E+07	7.29E+05	0.89	0.76	2.85	0.03	22.47	0.19	0.11	0.23	0.01	0.29	13.74	0.03
GVMSLVNVR	Q9P2R3	Rabankyrin-5	4.10E+05	4.10E+05	5.79E+05	1.27E+05	2.00E+05	1.00	1.00	2.05	0.39	0.71	0.80	2.90	0.17	4.55	0.31	0.64	0.75

EFVAVTGA EEDR	Q9UNZ2	NSFL1 cofactor p47	2.84E+06	1.22E+06	1.39E+06	1.34E+06	4.26E+06	2.32	0.16	0.29	0.04	2.03	0.51	0.33	0.30	1.04	0.97	0.31	0.32
FVTAVGTQ GAISK	O14786	Neuropilin-1	2.26E+06	2.26E+06	5.75E+05	3.09E+06	1.56E+06	1.00	1.00	1.45	0.37	3.93	0.27	0.37	0.40	0.19	0.16	1.98	0.30
SFEGNNNY DTPELR	O14786	Neuropilin-1	3.40E+06	2.72E+06	9.46E+05	3.53E+06	1.46E+06	1.25	0.62	1.86	0.32	3.59	0.31	0.65	0.78	0.27	0.28	2.42	0.34
SELEEQLTP VAEETR	P02649	Apolipoprotein E	1.43E+08	1.30E+08	8.93E+07	1.09E+08	1.01E+08	1.10	0.72	1.28	0.35	1.60	0.42	0.88	0.81	0.82	0.77	1.08	0.88
LGPLVEQGR	P02649	Apolipoprotein E	1.23E+08	1.09E+08	9.70E+07	1.07E+08	7.72E+07	1.13	0.59	1.41	0.15	1.27	0.59	1.26	0.58	0.90	0.88	1.39	0.38
AATVGSLA GQPLQER	P02649	Apolipoprotein E	6.51E+07	5.20E+07	4.70E+07	5.87E+07	3.03E+07	1.25	0.52	1.72	0.13	1.39	0.62	1.55	0.32	0.80	0.76	1.93	0.13
EGTCPEAPT DECKPVK	P02787	Serotransferrin	9.21E+07	8.69E+07	1.84E+07	1.13E+08	6.43E+07	1.06	0.83	1.35	0.26	5.01	0.11	0.29	0.11	0.16	0.02	1.76	0.15
CDEWSVNS VGK	P02787	Serotransferrin	1.62E+08	1.56E+08	5.05E+07	2.09E+08	6.91E+07	1.04	0.90	2.25	0.01	3.21	0.22	0.73	0.56	0.24	0.02	3.02	0.00
EGYYGYTG AFR	P02787	Serotransferrin	1.08E+08	1.34E+08	5.74E+07	8.98E+07	6.13E+07	0.81	0.51	2.18	0.01	1.89	0.46	0.94	0.91	0.64	0.51	1.47	0.48
NSLPDTVQI R	P56537	Eukaryotic translation initiation factor 6	8.84E+07	1.08E+08	5.95E+07	1.70E+08	5.81E+07	0.82	0.38	1.87	0.03	1.49	0.42	1.03	0.95	0.35	0.14	2.92	0.00
LSALGNVTT CNDYVALV HPDLDR	P56537	Eukaryotic translation initiation factor 6	1.39E+07	2.09E+07	1.93E+07	1.19E+07	9.87E+06	0.66	0.10	2.12	0.02	0.72	0.41	1.95	0.11	1.61	0.34	1.21	0.72
ETEEILADV LK	P56537	Eukaryotic translation initiation factor 6	5.47E+07	6.38E+07	3.83E+07	3.85E+07	4.85E+07	0.86	0.60	1.31	0.41	1.43	0.54	0.79	0.65	1.00	0.99	0.79	0.68
LVEPGSPAE K	O14745	Na(+)/H(+) exchange regulatory cofactor NHE-RF1	1.21E+07	1.80E+07	1.10E+07	1.92E+07	7.04E+06	0.68	0.13	2.55	0.01	1.11	0.83	1.56	0.31	0.57	0.38	2.73	0.00

LVEVNGENVEK	O14745	Na(+)/H(+) exchange regulatory cofactor NHE-RF1	8.15E+06	1.80E+07	3.88E+06	2.09E+07	4.59E+06	0.45	0.00	3.93	0.00	2.10	0.30	0.84	0.80	0.19	0.03	4.56	0.00
SVDPDSPAEASGLR	O14745	Na(+)/H(+) exchange regulatory cofactor NHE-RF1	1.70E+07	3.25E+07	1.40E+07	4.01E+07	8.88E+06	0.52	0.01	3.66	0.00	1.21	0.76	1.57	0.33	0.35	0.07	4.52	0.00
IQNTLHCCGVTDYR	O43657	Tetraspanin-6	9.83E+05	5.14E+05	0.00	7.55E+05	1.56E+05	1.91	0.52	3.30	0.23	—	—	—	—	—	—	4.85	0.14
ESLSSYWESAK	P02655	Apolipoprotein C-II	8.03E+06	2.76E+06	3.77E+06	3.58E+05	8.09E+06	2.91	0.15	0.34	0.03	2.13	—	0.47	—	10.53	0.30	0.04	0.13
GLQYAAQEGLLALQSEL[illegible]	P18428	Lipopolysaccharide-binding protein	8.55E+05	4.86E+05	4.73E+05	2.02E+05	1.41E+05	1.76	0.28	3.44	0.07	1.81	—	3.35	—	2.35	0.46	1.43	0.70
LAEGFPLPLLK	P18428	Lipopolysaccharide-binding protein	4.57E+06	2.69E+06	1.31E+06	2.03E+06	2.38E+06	1.70	0.36	1.13	0.83	3.50	—	0.55	—	0.65	0.71	0.85	0.89
ALLSPEQR	Q10588	ADP-ribosyl cyclase/cyclic ADP-ribose hydrolase 2	1.96E+07	2.92E+07	4.28E+07	2.50E+07	1.04E+07	0.67	0.09	2.80	0.00	0.46	—	4.11	—	1.71	0.56	2.40	0.07
GFFADYEIPNLQK	Q10588	ADP-ribosyl cyclase/cyclic [illegible]	7.34E+06	1.02E+07	1.77E+07	6.57E+06	4.84E+06	0.72	0.15	2.11	0.00	0.41	—	3.66	—	2.70	0.09	1.36	0.45
LLLIGDSGVGK	P62820	Ras-related protein Rab-1A	2.59E+07	3.30E+07	3.24E+07	1.76E+07	1.63E+07	0.78	0.32	2.02	0.01	0.80	—	1.99	—	1.84	0.52	1.08	0.87
LQIWDTAGQER	P62820	Ras-related protein Rab-1A	4.64E+07	5.70E+07	4.02E+07	5.86E+07	3.77E+07	0.82	0.37	1.51	0.15	1.16	—	1.06	—	0.69	0.59	1.55	0.40
TITSSYYR	P62820	Ras-related protein Rab-1A	3.85E+06	7.91E+06	2.31E+06	6.05E+06	3.15E+06	0.49	0.03	2.51	0.03	1.67	—	0.73	—	0.38	0.20	1.92	0.13
DLGGFDEDAEPR	Q9BRK5	45 kDa calcium-binding protein	2.75E+07	1.97E+07	1.99E+07	2.61E+07	1.70E+07	1.40	0.20	1.16	0.52	1.39	—	1.17	—	0.76	0.68	1.54	0.29

DPVVAYYCR	Q9NP79	Vacuolar protein sorting-associated protein VTA1 homolog	7.20E+06	8.97E+06	2.98E+05	7.07E+06	2.83E+06	0.80	0.59	3.16	0.03	24.13	—	0.11	—	0.04	0.01	2.50	0.22
LYAMQTGMK	Q9NP79	Vacuolar protein sorting-associated protein VTA1 homolog	1.03E+07	1.38E+07	7.46E+06	7.15E+06	2.79E+06	0.75	0.51	4.93	0.03	1.39	—	2.67	—	1.04	0.97	2.56	0.22
LMDQLEALK	Q9NP79	Vacuolar protein sorting-associated protein VTA1 homolog	1.52E+07	1.70E+07	2.74E+07	4.74E+06	1.24E+07	0.89	0.72	1.37	0.41	0.56	—	2.21	—	5.77	0.24	0.38	0.26
LLIYDASSLESGVPSR	A0A0B4J2D9	Immunoglobulin kappa variable 1D-13	1.49E+07	1.90E+07	2.09E+07	1.31E+07	6.66E+06	0.79	0.68	2.84	0.05	0.72	—	3.13	—	1.59	0.62	1.97	0.19
GDTEGTFGLDWEPDSGH[illegible]	O75309	Cadherin-16	5.91E+06	7.82E+06	5.63E+06	3.33E+06	3.82E+06	0.76	0.30	2.05	0.04	1.05	—	1.47	—	1.69	0.55	0.87	0.77
QQDVLGFLEANK	O75368	Adapter SH3BGRL	4.25E+06	4.09E+06	4.33E+06	3.13E+06	1.94E+06	1.04	0.91	2.11	0.08	0.98	—	2.24	—	1.38	0.54	1.62	0.38
LMNSQLVTTEK	P15907	Beta-galactoside alpha-2,6-sialyltransferase 1	5.71E+06	6.29E+06	2.19E+06	9.88E+06	2.48E+06	0.91	0.79	2.54	0.03	2.61	—	0.88	—	0.22	0.10	3.99	0.01
ATLPGFR	P15907	Beta-galactoside alpha-2,6-sialyltransferase 1	4.89E+06	5.40E+06	1.95E+06	6.39E+06	3.01E+06	0.91	0.69	1.79	0.13	2.51	—	0.65	—	0.31	0.30	2.12	0.24
LYTLVLTDPDAPSR	P30086	Phosphatidylethanolamine-binding protein 1	1.86E+08	2.35E+08	1.93E+08	1.46E+08	1.11E+08	0.79	0.26	2.12	0.00	0.97	—	1.73	—	1.32	0.67	1.31	0.57
CDEPILSNR	P30086	Phosphatidylethanolamine-binding protein 1	1.37E+08	1.46E+08	1.51E+08	2.16E+08	8.88E+07	0.94	0.77	1.64	0.06	0.91	—	1.70	—	0.70	0.60	2.43	0.04

LYEQLSGK	P30086	Phosphatidylethanolamine-binding protein 1	1.27E+08	1.62E+08	1.66E+08	1.98E+08	7.95E+07	0.79	0.20	2.03	0.01	0.76	—	2.09	—	0.84	0.83	2.49	0.06
EVLLLVHNLPQDPR	P31997	Carcinoembryonic antigen-related cell adhesion molecule 8	5.15E+06	6.31E+06	3.56E+06	1.42E+05	1.06E+06	0.82	0.74	5.95	0.07	1.44	—	3.36	—	25.15	0.34	0.13	0.63
IIGYVISNQQITPGPAYSNR	P31997	Carcinoembryonic antigen-related cell adhesion molecule 8	3.09E+06	2.06E+06	0.00	5.56E+05	1.94E+05	1.50	0.77	10.58	0.08	—	—	—	—	—	—	2.86	0.24
TLTLLSVTR	P31997	Carcinoembryonic antigen-related cell adhesion molecule 8	1.39E+07	2.17E+07	2.37E+07	1.09E+07	8.89E+06	0.64	0.05	2.44	0.00	0.59	—	2.67	—	2.18	0.35	1.22	0.74
SPHWGSTYSVSVVETDYDQYALLYSQGSK	P41222	Prostaglandin-H2 D-isomerase	2.27E+08	2.82E+08	4.16E+08	1.06E+08	2.66E+08	0.80	0.52	1.06	0.88	0.54	—	1.56	—	3.93	0.04	0.40	0.40
AQGFTEDTIVFLPQTDK	P41222	Prostaglandin-H2 D-isomerase	1.47E+09	2.28E+09	2.63E+09	1.25E+09	1.48E+09	0.64	0.05	1.54	0.11	0.56	—	1.78	—	2.12	0.03	0.84	0.66
ETNLLYDPK	Q07075	Glutamyl aminopeptidase	1.09E+07	1.67E+07	4.76E+06	1.25E+07	4.05E+06	0.65	0.12	4.12	0.00	2.28	—	1.17	—	0.38	0.28	3.08	0.03
GCQMYLEK	Q07075	Glutamyl aminopeptidase	1.02E+07	1.23E+07	5.13E+06	8.31E+06	3.36E+06	0.83	0.60	3.65	0.00	1.98	—	1.53	—	0.62	0.57	2.47	0.07
TSDFWAALEEASR	Q07075	Glutamyl aminopeptidase	1.03E+07	1.41E+07	1.56E+07	5.02E+06	5.85E+06	0.73	0.32	2.41	0.03	0.66	—	2.67	—	3.11	0.40	0.86	0.81
AVSVEAAVTPAEPYAR	Q24JP5	Transmembrane protein 132A	1.51E+06	1.80E+06	4.12E+05	1.43E+06	4.57E+05	0.84	0.66	3.94	0.02	3.66	—	0.90	—	0.29	0.47	3.14	0.22
AEELVNTAPLTGVPQHVPVR	Q24JP5	Transmembrane protein 132A	3.89E+06	6.62E+06	2.59E+06	4.81E+06	2.33E+06	0.59	0.10	2.85	0.02	1.50	—	1.11	—	0.54	0.43	2.07	0.36
EPGVTSIEVR	Q24JP5	Transmembrane protein 132A	5.23E+06	4.69E+06	4.16E+06	1.01E+07	2.98E+06	1.12	0.73	1.57	0.22	1.26	—	1.39	—	0.41	0.25	3.38	0.02

LDKEESPAP WDR	Q8NHJ6	Leukocyte immunoglobulin-like receptor subfamily B member 4	9.20E+06	1.51E+07	4.97E+06	9.88E+06	7.82E+06	0.61	0.02	1.93	0.01	1.85	—	0.64	—	0.50	0.19	1.26	0.56
FSIPSMTED YAGR	Q8NHJ6	Leukocyte immunoglobulin-like receptor subfamily B member 4	1.70E+07	3.39E+07	1.95E+07	1.80E+07	1.20E+07	0.50	0.00	2.83	0.00	0.87	—	1.63	—	1.08	0.88	1.51	0.29
SVTLLCQSR	Q8NHJ6	Leukocyte immunoglobulin-like receptor subfamily B member 4	5.98E+06	1.60E+07	7.29E+06	5.93E+06	4.38E+06	0.37	0.00	3.64	0.00	0.82	—	1.66	—	1.23	0.75	1.35	0.64
VGPQVPLSE PGFR	Q9H6S3	Epidermal growth factor receptor kinase substrate 8-like protein 2	1.17E+07	1.08E+07	9.14E+06	1.92E+07	3.78E+06	1.09	0.82	2.85	0.01	1.28	—	2.42	—	0.48	0.42	5.09	0.01
SQPVSQPLT YESGPDEVR	Q9H6S3	Epidermal growth factor receptor kinase substrate 8-like protein 2	4.97E+06	4.84E+06	9.59E+05	1.28E+07	1.45E+06	1.03	0.93	3.34	0.00	5.18	—	0.66	—	0.07	0.00	8.85	0.00
QYIISEELIS EGK	Q9UKK9	ADP-sugar pyrophosphatase	7.73E+05	1.09E+06	6.57E+05	1.14E+06	4.06E+05	0.71	0.35	2.68	0.01	1.18	—	1.62	—	0.57	0.46	2.82	0.06
EQTADGVA VIPVLQR	Q9UKK9	ADP-sugar pyrophosphatase	3.27E+06	5.43E+06	1.99E+06	5.41E+06	1.71E+06	0.60	0.12	3.18	0.02	1.64	—	1.17	—	0.37	0.32	3.17	0.06
VGLYTGEIS TTR	Q9UN74	Protocadherin alpha-4	1.03E+07	1.02E+07	4.68E+06	7.99E+06	4.69E+06	1.01	0.99	2.18	0.03	2.19	—	1.00	—	0.59	0.37	1.71	0.22
FDSDVGEFR	P01911	HLA class II histocompatibility antigen, DRB1 beta chain	3.97E+06	5.71E+06	3.06E+06	3.96E+06	1.33E+06	0.70	0.38	4.29	0.03	1.30	—	2.29	—	0.77	0.66	2.98	0.02

VGWEQLLTTIAR	O43707	Alpha-actinin-4	3.24E+05	8.29E+05	0.00	6.14E+04	8.35E+05	0.39	0.12	0.99	0.99	—	—	—	—	—	—	0.07	0.42
GISQEQMQEFR	O43707	Alpha-actinin-4	8.46E+06	3.62E+06	6.00E+06	1.88E+06	3.10E+06	2.34	0.13	1.17	0.73	1.41	—	1.94	—	3.19	0.33	0.61	0.50
LSWYDPDFQAR	P06127	T-cell surface glycoprotein CD5	2.61E+06	2.41E+06	2.84E+06	2.39E+06	7.18E+05	1.08	0.84	3.36	0.02	0.92	—	3.96	—	1.19	0.86	3.33	0.10
LASYLDK	P08727	Keratin, type I cytoskeletal 19	7.59E+07	7.66E+07	2.42E+08	7.23E+07	1.28E+08	0.99	0.98	0.60	0.29	0.31	—	1.89	—	3.34	0.39	0.56	0.56
VAIEPGAPR	Q00796	Sorbitol dehydrogenase	1.97E+07	1.61E+07	1.51E+07	2.71E+07	1.04E+07	1.22	0.52	1.56	0.24	1.30	—	1.46	—	0.56	0.46	2.62	0.08
LPDNVTFEEGALIEPLSVGIHACR	Q00796	Sorbitol dehydrogenase	1.01E+06	1.23E+06	1.05E+06	9.29E+05	7.59E+05	0.83	0.70	1.61	0.46	0.96	—	1.39	—	1.13	0.88	1.22	0.83
AMGAAQVVVTDLSATR	Q00796	Sorbitol dehydrogenase	2.55E+06	4.47E+06	1.42E+06	1.74E+06	1.39E+06	0.57	0.14	3.21	0.06	1.80	—	1.02	—	0.81	0.83	1.25	0.86
VCIAQGYQR	Q92563	Testican-2	1.26E+07	1.40E+07	5.21E+06	1.87E+07	1.73E+06	0.90	0.79	8.10	0.00	2.42	—	3.02	—	0.28	0.27	10.82	0.00
DWFQLLHENSK	Q92563	Testican-2	1.92E+06	2.88E+06	4.89E+06	1.04E+06	1.22E+06	0.67	0.22	2.37	0.04	0.39	—	4.02	—	4.68	0.08	0.86	0.79
EKPPCLAELER	Q92563	Testican-2	4.34E+06	5.50E+06	6.79E+06	5.94E+06	3.83E+06	0.79	0.32	1.44	0.20	0.64	—	1.77	—	1.14	0.74	1.55	0.36
DLGEAALNEYLR	O75891	Cytosolic 10-formyltetrahydrofolate dehydrogenase	6.39E+06	5.65E+06	2.25E+06	3.92E+06	6.51E+06	1.13	0.70	0.87	0.82	2.83	—	0.35	—	0.57	0.51	0.60	0.73
WTLGFCDER	O95336	6-phosphogluconolactonase	1.74E+07	2.53E+07	3.87E+07	1.77E+07	1.62E+07	0.69	0.15	1.56	0.15	0.45	—	2.39	—	2.19	0.56	1.09	0.89
IVAPISDSPKPPPQR	O95336	6-phosphogluconolactonase	2.10E+07	3.48E+07	2.90E+07	3.55E+07	1.95E+07	0.60	0.06	1.78	0.09	0.72	—	1.48	—	0.81	0.82	1.82	0.25

DEELSCTVV ELK	P01011	Alpha-1-antichymotrypsin	5.87E+07	6.99E+07	4.50E+07	3.26E+07	3.62E+07	0.84	0.60	1.93	0.09	1.31	—	1.24	—	1.38	0.64	0.90	0.90
MEEVEAML LPETLK	P01011	Alpha-1-antichymotrypsin	1.15E+07	1.56E+07	4.23E+06	1.14E+07	1.44E+07	0.74	0.59	1.08	0.91	2.72	—	0.29	—	0.37	0.44	0.79	0.88
GILAADESV GTMGNR	P05062	Fructose-bisphosphate aldolase B	8.02E+07	1.01E+08	5.62E+07	6.36E+07	3.61E+07	0.79	0.42	2.81	0.01	1.43	—	1.56	—	0.88	0.86	1.76	0.24
LDQGGAPL AGTNK	P05062	Fructose-bisphosphate aldolase B	1.07E+08	1.38E+08	4.55E+07	1.18E+08	5.96E+07	0.78	0.46	2.31	0.03	2.36	—	0.76	—	0.39	0.17	1.97	0.15
ETTIQGLDG LSER	P05062	Fructose-bisphosphate aldolase B	1.18E+08	1.71E+08	5.98E+07	1.51E+08	7.97E+07	0.69	0.15	2.15	0.04	1.97	—	0.75	—	0.39	0.15	1.90	0.25
KTLLSNLEE AK	P10909	Clusterin	7.34E+06	1.58E+07	7.36E+05	3.32E+07	7.21E+06	0.46	0.17	2.20	0.25	9.97	—	0.10	—	0.02	0.31	4.60	0.13
IDSLLENDR	P10909	Clusterin	6.07E+08	6.22E+08	4.44E+08	1.87E+08	3.87E+08	0.98	0.91	1.61	0.03	1.37	—	1.15	—	2.37	0.29	0.48	0.15
VVATTQMQ AADAR	P15144	Aminopeptidase N	1.77E+08	1.33E+08	1.35E+08	1.19E+08	8.35E+07	1.33	0.40	1.59	0.31	1.31	—	1.62	—	1.14	0.86	1.42	0.67
AVGFGGDF DGVPR	P16444	Dipeptidase 1	9.78E+07	1.01E+08	7.16E+07	8.53E+07	6.21E+07	0.97	0.89	1.63	0.15	1.37	—	1.15	—	0.84	0.82	1.37	0.62
VPEGLEDVS KYPDLIAEL [illegible]	P16444	Dipeptidase 1	1.72E+06	2.98E+06	8.15E+05	8.25E+06	1.84E+05	0.58	0.46	16.20	0.12	2.11	—	4.43	—	0.10	0.34	44.85	0.02
CLAFTDVAP R	P19801	Amiloride-sensitive amine oxidase	1.65E+07	2.15E+07	1.10E+07	3.08E+07	1.57E+07	0.77	0.29	1.37	0.26	1.51	—	0.70	—	0.36	0.09	1.96	0.07
ALCLFEMPT GVPLR	P19801	Amiloride-sensitive amine oxidase	2.81E+06	3.37E+06	1.39E+06	4.16E+06	1.93E+06	0.83	0.57	1.75	0.15	2.03	—	0.72	—	0.33	0.15	2.16	0.11
DTVIVWPR	P19801	Amiloride-sensitive amine oxidase	1.83E+07	2.47E+07	1.69E+07	1.54E+07	1.49E+07	0.74	0.24	1.67	0.09	1.08	—	1.14	—	1.10	0.92	1.04	0.95

EVQEFYK	P21926	CD9 antigen	8.06E+07	7.35E+07	5.52E+07	4.96E+07	7.76E+07	1.10	0.69	0.95	0.84	1.46	—	0.71	—	1.11	0.90	0.64	0.35
AIHYALNCCGLAGGVEQ[illegible]	P21926	CD9 antigen	2.17E+06	2.41E+06	5.06E+05	8.32E+05	2.04E+06	0.90	0.86	1.18	0.84	4.29	—	0.25	—	0.61	0.66	0.41	0.71
KDVLETFTVK	P21926	CD9 antigen	1.39E+07	2.98E+07	3.06E+06	1.36E+07	1.28E+07	0.47	0.03	2.34	0.06	4.52	—	0.24	—	0.23	0.24	1.06	0.94
VDLTQFK	P50053	Ketohexokinase	7.95E+06	8.82E+06	4.34E+06	8.84E+06	6.48E+06	0.90	0.70	1.36	0.44	1.83	—	0.67	—	0.49	0.33	1.36	0.68
HLGFQSAEEALR	P50053	Ketohexokinase	2.76E+06	3.44E+06	1.88E+06	9.66E+05	1.38E+06	0.80	0.57	2.50	0.09	1.47	—	1.36	—	1.94	0.46	0.70	0.67
ATAPVSFNYYGVVTGPSASK	Q5VW32	BRO1 domain-containing protein BROX	6.78E+06	9.21E+06	5.82E+06	7.79E+06	4.44E+06	0.74	0.43	2.08	0.19	1.16	—	1.31	—	0.75	0.77	1.75	0.56
ADHTLSSLEPAYSAK	Q5VW32	BRO1 domain-containing protein BROX	1.87E+07	2.11E+07	2.36E+07	4.38E+07	1.39E+07	0.89	0.67	1.52	0.23	0.79	—	1.69	—	0.54	0.39	3.14	0.01
IPTEAPQLELK	Q5VW32	BRO1 domain-containing protein BROX	8.48E+07	9.38E+07	7.92E+07	1.08E+08	5.28E+07	0.90	0.70	1.78	0.13	1.07	—	1.50	—	0.74	0.70	2.04	0.14
ISGQEVNEAACDIAR	Q93088	Betaine--homocysteine S-methyltransferase 1	7.05E+07	7.71E+07	2.84E+07	1.07E+08	3.93E+07	0.91	0.75	1.96	0.06	2.48	—	0.72	—	0.26	0.11	2.74	0.06
EAYNLGVR	Q93088	Betaine--homocysteine S-methyltransferase 1	5.43E+07	5.94E+07	2.31E+07	6.80E+07	3.03E+07	0.91	0.76	1.96	0.05	2.35	—	0.76	—	0.34	0.16	2.24	0.11
AIAEELAPER	Q93088	Betaine--homocysteine S-methyltransferase 1	1.38E+08	1.29E+08	6.29E+07	1.52E+08	6.77E+07	1.07	0.85	1.90	0.04	2.19	—	0.93	—	0.41	0.18	2.25	0.06

EVWDYVTVR	Q9HB40	Retinoid-inducible serine carboxypeptidase	2.63E+07	2.37E+07	2.37E+07	1.23E+07	1.87E+07	1.11	0.73	1.27	0.43	1.11	—	1.27	—	1.93	0.42	0.66	0.55
VAEQVLNAVNK	Q9HB40	Retinoid-inducible serine carboxypeptidase	2.79E+07	3.02E+07	1.87E+07	1.55E+07	1.53E+07	0.92	0.79	1.97	0.04	1.49	—	1.22	—	1.20	0.80	1.01	0.98
DALSQLMNGPIR	Q9HB40	Retinoid-inducible serine carboxypeptidase	1.09E+07	1.06E+07	1.18E+07	7.81E+06	6.06E+06	1.02	0.93	1.75	0.10	0.92	—	1.95	—	1.51	0.59	1.29	0.69
LLAPAALAFR	O60241	Adhesion G protein-coupled receptor B2	1.77E+06	1.51E+06	1.11E+06	1.80E+06	2.52E+06	1.17	0.67	0.60	0.15	1.59	—	0.44	—	0.62	0.54	0.71	0.57
WVFKEEDPIHLR	O75015	Low affinity immunoglobulin gamma Fc region receptor III-B	1.09E+07	1.41E+07	1.20E+07	2.35E+07	7.74E+05	0.77	0.81	18.21	0.08	0.91	—	15.47	—	0.51	0.65	30.42	0.03
LEQELPAR	O95502	Neuronal pentraxin receptor	2.04E+07	4.71E+06	7.37E+06	1.01E+06	2.92E+07	4.32	0.07	0.16	0.00	2.76	—	0.25	—	7.28	0.32	0.03	0.08
IPAMTIAK	P10809	60 kDa heat shock protein, mitochondrial	2.71E+06	3.28E+06	5.53E+05	1.59E+06	6.32E+05	0.83	0.70	5.19	0.05	4.90	—	0.87	—	0.35	0.15	2.52	0.11
LLCEVTPVSGQER	P18627	Lymphocyte activation gene 3 protein	1.89E+06	1.70E+06	3.09E+06	2.76E+06	1.23E+06	1.11	0.59	1.38	0.26	0.61	—	2.52	—	1.12	0.86	2.25	0.05
GEILFFK	P22894	Neutrophil collagenase	2.63E+05	4.39E+05	2.84E+05	1.39E+06	4.16E+05	0.60	0.37	1.05	0.92	0.92	—	0.68	—	0.20	0.21	3.35	0.07
SISGAFPGIESK	P22894	Neutrophil collagenase	7.75E+05	1.66E+06	0.00	1.38E+06	4.88E+05	0.47	0.11	3.40	0.06	—	—	—	—	—	—	2.82	0.15

YVESFR	P50135	Histamine N-methyltransferase	7.04E+06	5.73E+06	2.27E+06	4.44E+06	2.69E+06	1.23	0.60	2.13	0.05	3.10	—	0.85	—	0.51	0.46	1.65	0.42
ECIYYNANWELER	Q13705	Activin receptor type-2B	3.61E+05	4.08E+05	2.49E+06	4.96E+05	1.39E+05	0.88	0.79	2.94	0.14	0.15	—	17.92	—	5.01	0.48	3.58	0.07
GCWLDDFNCYDR	Q13705	Activin receptor type-2B	2.27E+06	1.65E+06	8.84E+06	1.79E+06	2.97E+06	1.38	0.54	0.56	0.24	0.26	—	2.98	—	4.94	0.21	0.60	0.51
LLGCAVLAPR	Q66K79	Carboxypeptidase Z	3.66E+06	3.62E+06	3.08E+06	9.90E+06	7.48E+06	1.01	0.97	0.48	0.11	1.19	—	0.41	—	0.31	0.39	1.32	0.69
CEGGWVR	Q66K79	Carboxypeptidase Z	4.51E+06	2.46E+06	5.55E+06	9.81E+06	1.07E+07	1.83	0.29	0.23	0.01	0.81	—	0.52	—	0.57	0.52	0.92	0.90
ARDDCEPLMK	Q6FHJ7	Secreted frizzled-related protein 4	8.39E+06	8.64E+06	2.11E+07	2.17E+07	9.94E+06	0.97	0.93	0.87	0.74	0.40	—	2.12	—	0.97	0.97	2.19	0.13
DDCEPLMK	Q6FHJ7	Secreted frizzled-related protein 4	5.32E+06	3.88E+06	7.32E+06	1.62E+06	6.04E+06	1.37	0.36	0.64	0.19	0.73	—	1.21	—	4.51	0.28	0.27	0.07
DFVNMMLGK	Q9BQI0	Allograft inflammatory factor 1-like	7.24E+05	1.13E+06	2.06E+06	6.16E+05	3.98E+05	0.64	0.26	2.83	0.06	0.35	—	5.17	—	3.34	0.39	1.55	0.72
LVMMFEGK	Q9BQI0	Allograft inflammatory factor 1-like	4.85E+05	7.13E+05	1.93E+05	0.00	2.06E+05	0.68	0.44	3.47	0.06	2.52	—	0.94	—	—	0.20	—	—
DWVIPPISCPENEK	P12830	Cadherin-1	2.85E+08	4.03E+08	4.03E+08	3.01E+08	2.12E+08	0.71	0.09	1.90	0.01	0.71	—	1.90	—	1.34	0.67	1.42	0.55
DTANWLEINPDTGAISTR	P12830	Cadherin-1	2.64E+08	2.73E+08	2.88E+08	2.86E+08	1.37E+08	0.97	0.88	1.98	0.00	0.92	—	2.10	—	1.01	0.99	2.08	0.17
MALEVGDYK	P12830	Cadherin-1	3.06E+08	3.67E+08	5.40E+08	2.56E+08	3.00E+08	0.83	0.38	1.22	0.37	0.57	—	1.80	—	2.11	0.11	0.86	0.77
TFLVGNLEIR	O75339	Cartilage intermediate layer protein 1	1.55E+06	7.81E+05	2.61E+06	1.20E+06	2.78E+06	1.98	0.18	0.28	0.03	0.59	—	0.94	—	2.17	0.43	0.43	0.41

TGFLSNPR	O75339	Cartilage intermediate layer protein 1	4.19E+06	4.11E+06	3.83E+06	2.54E+06	7.93E+06	1.02	0.95	0.52	0.08	1.10	—	0.48	—	1.50	0.70	0.32	0.21
ACEEAPPSAAHFR	O75339	Cartilage intermediate layer protein 1	3.58E+06	2.94E+06	4.52E+06	4.82E+06	4.61E+06	1.22	0.70	0.64	0.34	0.79	—	0.98	—	0.94	0.93	1.04	0.95
EGMLQHWELGQALR	P11117	Lysosomal acid phosphatase	3.45E+07	4.23E+07	5.75E+07	1.78E+07	2.36E+07	0.82	0.61	1.79	0.23	0.60	—	2.44	—	3.23	0.09	0.76	0.61
FLFGIYQQAEK	P11117	Lysosomal acid phosphatase	1.37E+07	2.01E+07	2.53E+07	3.48E+06	9.77E+06	0.68	0.20	2.06	0.06	0.54	—	2.59	—	7.29	0.02	0.36	0.28
LQGGVLLAQIR	P11117	Lysosomal acid phosphatase	2.92E+07	4.04E+07	5.50E+07	5.98E+06	2.17E+07	0.72	0.32	1.86	0.16	0.53	—	2.53	—	9.19	0.10	0.28	0.20
GGPTPQEAIQR	Q9H444	Charged multivesicular body protein 4b	2.85E+07	2.65E+07	2.44E+07	4.62E+07	1.44E+07	1.08	0.76	1.84	0.07	1.17	—	1.69	—	0.53	0.34	3.20	0.00
NLLEISGPETVPLPNVPSIALPSKPAK	Q9H444	Charged multivesicular body protein 4b	4.66E+05	9.91E+05	0.00	0.00	9.35E+04	0.47	0.44	10.60	0.30	—	—	—	—	—	—	—	—
ASEQIYYENR	Q9H6X2	Anthrax toxin receptor 1	3.58E+07	3.96E+07	4.28E+07	8.00E+07	4.23E+07	0.91	0.79	0.94	0.87	0.84	—	1.01	—	0.53	0.50	1.89	0.29
DLGAIVYCVGVK	Q9H6X2	Anthrax toxin receptor 1	1.70E+06	2.46E+06	9.51E+05	2.51E+06	1.01E+06	0.69	0.34	2.43	0.07	1.79	—	0.94	—	0.38	0.46	2.48	0.16
VLCSFK	Q9H6X2	Anthrax toxin receptor 1	2.52E+07	1.79E+07	2.23E+07	1.44E+07	3.36E+07	1.41	0.27	0.53	0.02	1.13	—	0.67	—	1.56	0.64	0.43	0.16
VWDVIGR	O14618	Copper chaperone for superoxide dismutase	9.70E+05	1.25E+06	6.22E+05	8.88E+05	7.95E+05	0.78	0.43	1.57	0.11	1.56	—	0.78	—	0.70	0.62	1.12	0.82
DLLNCSFK	P43490	Nicotinamide phosphoribosyltransferase	4.98E+06	1.73E+07	1.64E+07	3.37E+06	1.05E+07	0.29	0.05	1.65	0.50	0.30	—	1.57	—	4.86	0.48	0.32	0.65

MTQDLAGTYR	P43626	Killer cell immunoglobulin-like receptor 2DL1	2.76E+06	4.39E+06	3.18E+06	1.83E+06	1.90E+06	0.63	0.31	2.31	0.17	0.87	—	1.67	—	1.74	0.43	0.96	0.96
LNLYELK	P49720	Proteasome subunit beta type-3	1.37E+06	1.69E+06	5.55E+05	1.33E+06	1.50E+06	0.81	0.59	1.13	0.82	2.46	—	0.37	—	0.42	0.26	0.89	0.91
FGPYYTEPVIAGLDPK	P49720	Proteasome subunit beta type-3	3.90E+05	1.72E+05	0.00	0.00	6.23E+04	2.26	0.48	2.77	0.52	—	—	—	—	—	—	—	—
FVWSEER	Q8N114	Protein shisa-5	4.32E+08	8.18E+07	2.92E+08	2.65E+07	5.99E+08	5.28	0.04	0.14	0.00	1.48	—	0.49	—	11.02	0.38	0.04	0.05
GLGSTVQEIDLTGVK	Q96CN7	Isochorismatase domain-containing protein 1	3.53E+05	1.17E+06	2.52E+05	1.97E+06	6.57E+04	0.30	0.06	17.89	0.04	1.40	—	3.84	—	0.13	0.25	30.06	0.01
QCWQLGSNLLK	Q9BXN2	C-type lectin domain family 7 member A	3.94E+06	7.27E+06	6.72E+06	3.96E+06	4.66E+06	0.54	0.03	1.56	0.20	0.59	—	1.44	—	1.70	0.46	0.85	0.80
IDSSNELGFIVK	Q9BXN2	C-type lectin domain family 7 member A	5.64E+06	8.35E+06	1.38E+07	4.46E+06	5.08E+06	0.68	0.06	1.64	0.03	0.41	—	2.72	—	3.10	0.12	0.88	0.76
DRPFVCAPSSK	P36897	TGF-beta receptor type-1	2.46E+06	1.68E+06	2.64E+06	5.30E+05	2.35E+06	1.47	0.54	0.72	0.39	0.93	—	1.13	—	4.99	0.11	0.23	0.19
TGSVTTTYCCNQDHCNK	P36897	TGF-beta receptor type-1	7.16E+05	5.69E+05	5.30E+05	3.60E+03	7.91E+05	1.26	0.60	0.72	0.42	1.35	—	0.67	—	147.16	0.18	0.00	0.08
LVEGEVYAVDER	Q9BVM4	Gamma-glutamylaminecyclotransferase	1.46E+07	2.24E+07	1.04E+07	3.45E+07	1.70E+07	0.65	0.09	1.32	0.39	1.40	—	0.61	—	0.30	0.04	2.03	0.12
FLDDFESCPALYQR	Q9BVM4	Gamma-glutamylaminecyclotransferase	1.01E+07	2.63E+07	3.74E+07	1.05E+07	7.70E+06	0.39	0.02	3.41	0.04	0.27	—	4.86	—	3.56	0.45	1.36	0.61

VQLLEDR	Q9BVM4	Gamma-glutamylaminecyclotransferase	1.42E+07	1.61E+07	3.07E+07	1.61E+07	1.53E+07	0.88	0.57	1.06	0.86	0.46	—	2.01	—	1.91	0.50	1.05	0.92
QYDSFTYR	Q9Y3Q3	Transmembrane emp24 domain-containing protein 3	5.36E+06	6.17E+06	4.57E+06	1.15E+07	5.72E+06	0.87	0.67	1.08	0.86	1.17	—	0.80	—	0.40	0.25	2.02	0.32
IIEETLALK	O15144	Actin-related protein 2/3 complex subunit 2	1.78E+06	2.48E+06	1.38E+06	2.01E+06	9.32E+05	0.72	0.35	2.66	0.05	1.28	—	1.48	—	0.69	0.66	2.16	0.24
AWAGIDLK	O43278	Kunitz-type protease inhibitor 1	1.30E+07	1.60E+07	2.41E+07	9.70E+06	9.00E+06	0.81	0.20	1.78	0.00	0.54	—	2.68	—	2.48	0.10	1.08	0.80
DVENTDWR	O43278	Kunitz-type protease inhibitor 1	1.29E+07	1.49E+07	7.57E+06	1.15E+07	1.13E+07	0.87	0.63	1.32	0.34	1.70	—	0.67	—	0.66	0.46	1.02	0.97
EEECILACR	O43278	Kunitz-type protease inhibitor 1	3.10E+07	4.24E+07	4.05E+07	4.59E+07	2.93E+07	0.73	0.09	1.45	0.14	0.77	—	1.38	—	0.88	0.76	1.57	0.29
SIHDFCLVSK	O43291	Kunitz-type protease inhibitor [illegible]	4.88E+07	6.99E+07	1.01E+08	1.20E+08	5.63E+07	0.70	0.20	1.24	0.46	0.49	—	1.79	—	0.84	0.79	2.14	0.06
CATVTENATGDLATSR	O43291	Kunitz-type protease inhibitor [illegible]	1.39E+06	5.51E+06	1.91E+06	3.47E+06	2.07E+06	0.25	0.01	2.66	0.13	0.73	—	0.92	—	0.55	0.35	1.68	0.33
WYFDVER	O43291	Kunitz-type protease inhibitor [illegible]	9.31E+05	1.50E+06	1.93E+06	6.28E+05	1.02E+06	0.62	0.06	1.47	0.17	0.48	—	1.90	—	3.07	0.19	0.62	0.51
TPEELLR	O43633	Charged multivesicular [illegible]	6.10E+07	5.14E+07	6.72E+07	8.98E+07	3.87E+07	1.19	0.55	1.33	0.40	0.91	—	1.74	—	0.75	0.71	2.32	0.06
IMMEFER	O43633	Charged multivesicular [illegible]	3.64E+07	4.64E+07	5.98E+07	2.39E+07	1.67E+07	0.78	0.48	2.79	0.03	0.61	—	3.59	—	2.50	0.41	1.43	0.59

GIVEESVTG VHR	O43865	S-adenosylhomocysteine hydrolase-like protein 1	5.19E+05	7.74E+05	8.44E+05	1.21E+06	2.05E+05	0.67	0.38	3.78	0.01	0.62	—	4.12	—	0.70	0.68	5.91	0.00
TQSSLVPAL TDFVR	O60888	Protein CutA	7.88E+07	6.22E+07	9.16E+07	4.48E+07	4.59E+07	1.27	0.37	1.35	0.20	0.86	—	2.00	—	2.04	0.07	0.98	0.95
VALNVSCA NLLDK	O75131	Copine-3	1.69E+06	2.42E+06	3.50E+03	5.26E+06	2.02E+05	0.70	0.40	11.98	0.00	484.02	—	0.02	—	0.00	0.12	26.09	0.00
SPLGEVAIR	O75131	Copine-3	9.11E+06	8.01E+06	9.29E+06	4.73E+06	6.92E+06	1.14	0.64	1.16	0.65	0.98	—	1.34	—	1.97	0.53	0.68	0.61
SIISMFDR	O75340	Programmed cell death protein 6	1.83E+06	2.51E+06	2.55E+06	4.31E+05	4.77E+06	0.73	0.38	0.53	0.15	0.72	—	0.53	—	5.91	0.08	0.09	0.12
LSDQFHDILI R	O75340	Programmed cell death protein 6	1.89E+06	1.82E+06	1.59E+06	2.76E+05	3.95E+06	1.04	0.92	0.46	0.06	1.19	—	0.40	—	5.78	0.24	0.07	0.08
FVTHVSDW GALATISTL [illegible]	O75629	Protein CREG1	4.36E+06	3.91E+06	3.38E+06	1.38E+06	2.15E+06	1.12	0.86	1.81	0.39	1.29	—	1.57	—	2.45	0.56	0.64	0.67
TYMLAFDV NDEK	P02763	Alpha-1-acid glycoprotein 1	3.38E+08	6.89E+08	6.46E+08	9.52E+07	1.69E+08	0.49	0.07	4.08	0.01	0.52	—	3.83	—	6.78	0.02	0.56	0.32
NWGLSVYA DKPETTK	P02763	Alpha-1-acid glycoprotein 1	1.50E+08	3.28E+08	2.14E+08	3.11E+07	8.40E+07	0.46	0.02	3.91	0.01	0.70	—	2.54	—	6.88	0.00	0.37	0.11
SQVVAGTN YFIK	P04080	Cystatin-B	4.70E+06	7.10E+06	7.20E+06	2.22E+06	3.70E+06	0.66	0.14	1.92	0.07	0.65	—	1.95	—	3.24	0.15	0.60	0.41
VHVGDEDF VHLR	P04080	Cystatin-B	3.79E+06	6.53E+06	4.34E+06	5.53E+06	4.08E+06	0.58	0.12	1.60	0.27	0.87	—	1.06	—	0.78	0.75	1.36	0.49
SEDFGVNED LADSDAR	P04083	Annexin A1	2.85E+06	4.07E+06	9.30E+05	2.67E+06	3.67E+06	0.70	0.54	1.11	0.88	3.07	—	0.25	—	0.35	0.34	0.73	0.78
SEIDMNDIK	P04083	Annexin A1	2.35E+06	2.87E+06	7.62E+05	8.90E+05	2.30E+06	0.82	0.75	1.25	0.75	3.09	—	0.33	—	0.86	0.89	0.39	0.45
VTSLTACLV DQSLR	P04216	Thy-1 membrane glycoprotein	6.05E+06	4.70E+06	8.33E+06	4.49E+06	5.24E+06	1.29	0.34	0.90	0.68	0.73	—	1.59	—	1.86	0.24	0.86	0.77

CQEEVSHIPAVHPGSFRP[illegible]	P04233	HLA class II histocompatibility [illegible]	2.04E+06	2.71E+06	4.84E+05	4.80E+05	6.78E+05	0.75	0.69	3.99	0.32	4.21	—	0.71	—	1.01	0.99	0.71	0.80
SMVAVMDSDTTGK	P04632	Calpain small subunit 1	3.38E+06	6.53E+06	2.07E+06	3.61E+06	1.89E+06	0.52	0.01	3.45	0.00	1.63	—	1.09	—	0.57	0.44	1.91	0.19
CAPFFYGGCGGNR	P05067	Amyloid-beta precursor protein	1.68E+07	1.22E+07	1.19E+07	9.00E+06	1.67E+07	1.38	0.38	0.73	0.44	1.41	—	0.71	—	1.32	0.69	0.54	0.51
VESLEQEAANER	P05067	Amyloid-beta precursor protein	8.28E+06	1.11E+07	6.74E+06	6.30E+06	5.36E+06	0.74	0.41	2.08	0.17	1.23	—	1.26	—	1.07	0.94	1.17	0.81
VAGLSCEEMGFLR	P05981	Serine protease hepsin	8.39E+06	1.13E+07	1.16E+07	1.05E+07	5.90E+06	0.74	0.24	1.91	0.05	0.73	—	1.96	—	1.10	0.86	1.79	0.14
LLEVISVCDCPR	P05981	Serine protease hepsin	4.24E+06	6.80E+06	4.71E+06	6.65E+06	3.27E+06	0.62	0.04	2.08	0.01	0.90	—	1.44	—	0.71	0.60	2.03	0.08
GEFIWVDGSHVDYSNWAPGEPTSR	P06734	Low affinity immunoglobulin epsilon Fc receptor	1.18E+07	7.87E+06	2.06E+07	7.97E+06	1.26E+07	1.50	0.34	0.63	0.18	0.57	—	1.64	—	2.58	0.07	0.63	0.45
WNDAFCDR	P06734	Low affinity immunoglobulin epsilon Fc receptor	2.04E+07	1.17E+07	2.09E+07	3.76E+06	1.96E+07	1.75	0.20	0.60	0.06	0.98	—	1.06	—	5.55	0.01	0.19	0.01
LGAWVCDR	P06734	Low affinity immunoglobulin epsilon Fc receptor	1.34E+07	7.41E+06	2.12E+07	3.58E+06	1.20E+07	1.81	0.19	0.62	0.20	0.63	—	1.77	—	5.93	0.04	0.30	0.08
VIPGPPALTLVPAELVR	P07333	Macrophage colony-stimulating factor 1 receptor	4.59E+06	5.40E+06	6.99E+06	9.29E+06	4.70E+06	0.85	0.64	1.15	0.72	0.66	—	1.49	—	0.75	0.81	1.98	0.36
LAIPQQSDFHNNR	P07333	Macrophage colony-stimulating factor 1 receptor	4.04E+06	6.25E+06	6.44E+06	6.58E+06	4.48E+06	0.65	0.01	1.39	0.09	0.63	—	1.44	—	0.98	0.96	1.47	0.29
TSFPEDTVITYK	P08174	Complement decay-accelerating factor	9.59E+07	1.31E+08	1.46E+08	1.39E+08	6.28E+07	0.73	0.09	2.09	0.00	0.66	—	2.33	—	1.05	0.93	2.21	0.06

WSTAVEFCK	P08174	Complement decay-accelerating factor	4.25E+07	6.62E+07	1.04E+08	3.07E+07	4.08E+07	0.64	0.10	1.62	0.10	0.41	—	2.54	—	3.38	0.05	0.75	0.57
SCPNPGEIR	P08174	Complement decay-accelerating [illegible]	2.65E+08	3.64E+08	4.59E+08	3.24E+08	2.52E+08	0.73	0.06	1.45	0.06	0.58	—	1.82	—	1.41	0.31	1.29	0.38
KFQNSPAEDFQEPCQYSQESQK	P08887	Interleukin-6 receptor subunit alpha	2.05E+05	6.56E+05	2.36E+05	1.39E+06	7.20E+03	0.31	0.08	91.20	0.03	0.87	—	32.76	—	0.17	0.27	192.47	0.00
VTSLDTEVLTIK	P09131	P3 protein	2.29E+06	2.85E+06	4.92E+06	2.15E+06	2.42E+06	0.80	0.39	1.18	0.67	0.47	—	2.03	—	2.29	0.18	0.89	0.88
LHLDYIGPCK	P09486	SPARC	2.87E+06	4.95E+06	8.62E+06	4.05E+06	4.36E+06	0.58	0.09	1.14	0.71	0.33	—	1.98	—	2.13	0.25	0.93	0.89
STGGILDVYIFLGPEPK	P10253	Lysosomal alpha-glucosidase	1.84E+07	9.30E+06	1.05E+07	4.13E+06	4.29E+06	1.98	0.12	2.17	0.05	1.76	—	2.44	—	2.53	0.14	0.96	0.96
DFPAMVQELHQGGR	P10253	Lysosomal alpha-glucosidase	1.41E+08	1.62E+08	2.02E+08	6.76E+07	8.52E+07	0.87	0.62	1.90	0.03	0.70	—	2.37	—	2.99	0.09	0.79	0.71
VTSEGAGLQLQK	P10253	Lysosomal alpha-glucosidase	2.78E+08	2.88E+08	2.86E+08	2.50E+08	2.29E+08	0.96	0.85	1.26	0.41	0.97	—	1.25	—	1.14	0.78	1.09	0.87
ILLDEQAQWK	P13598	Intercellular adhesion molecule 2	5.40E+07	8.72E+07	1.63E+08	5.38E+07	5.89E+07	0.62	0.01	1.48	0.08	0.33	—	2.77	—	3.03	0.09	0.91	0.82
QVILTLQPTLVAVGK	P13598	Intercellular adhesion molecule 2	1.12E+07	1.54E+07	2.28E+07	7.31E+06	1.02E+07	0.73	0.21	1.51	0.25	0.49	—	2.23	—	3.12	0.22	0.71	0.73
ALDVSASDDEIAR	P13798	Acylamino-acid-releasing enzyme	3.51E+06	3.81E+06	1.98E+06	6.39E+06	2.61E+06	0.92	0.78	1.46	0.38	1.77	—	0.76	—	0.31	0.13	2.45	0.10
SALYYVDLIGGK	P13798	Acylamino-acid-releasing enzyme	2.04E+06	6.56E+06	1.90E+06	2.85E+06	8.79E+05	0.31	0.07	7.46	0.09	1.07	—	2.15	—	0.66	0.75	3.24	0.16
VTSVVVDVVPR	P13798	Acylamino-acid-releasing enzyme	7.59E+05	2.28E+06	1.05E+06	7.68E+05	3.52E+05	0.33	0.02	6.50	0.02	0.72	—	2.98	—	1.37	0.79	2.18	0.43

VEDSGHYY CVVR	P14778	Interleukin-1 receptor type 1	1.37E+06	2.34E+06	2.82E+06	1.88E+06	1.52E+06	0.58	0.12	1.54	0.21	0.49	—	1.85	—	1.50	0.33	1.24	0.71
VMAIQDFK PFENLR	P14784	Interleukin-2 receptor subunit beta	1.33E+06	1.41E+06	2.82E+06	8.44E+05	2.44E+06	0.94	0.87	0.58	0.17	0.47	—	1.16	—	3.34	0.31	0.35	0.26
HGESGSMA VFHQTQGPS [illegible]	P15151	Poliovirus receptor	1.88E+07	2.83E+07	4.98E+07	1.84E+07	1.86E+07	0.66	0.09	1.52	0.15	0.38	—	2.68	—	2.71	0.26	0.99	0.98
VQLTGEPVP MAR	P15151	Poliovirus receptor	1.27E+08	1.97E+08	2.03E+08	2.95E+08	7.67E+07	0.65	0.03	2.56	0.00	0.63	—	2.64	—	0.69	0.44	3.84	0.00
FTFQEAANE CR	P16112	Aggrecan core protein	1.32E+07	1.64E+07	2.34E+07	1.48E+07	1.66E+07	0.81	0.20	0.99	0.95	0.57	—	1.41	—	1.59	0.35	0.89	0.76
YPIVSPR	P16112	Aggrecan core protein	1.57E+08	1.52E+08	3.56E+08	1.37E+08	1.64E+08	1.03	0.89	0.93	0.78	0.44	—	2.17	—	2.59	0.31	0.84	0.68
YQCTEGFV QR	P16112	Aggrecan core protein	2.66E+07	3.27E+07	2.49E+07	3.20E+07	2.51E+07	0.81	0.26	1.30	0.25	1.07	—	0.99	—	0.78	0.53	1.28	0.49
VAVNDAHL LQYNHR	P17931	Galectin-3	1.66E+06	2.84E+06	1.20E+06	1.16E+06	1.39E+06	0.58	0.13	2.04	0.08	1.39	—	0.86	—	1.03	0.97	0.83	0.79
LSDPANWL K	P19022	Cadherin-2	8.53E+07	1.02E+08	1.48E+08	8.28E+07	5.20E+07	0.83	0.25	1.97	0.00	0.58	—	2.85	—	1.79	0.13	1.59	0.14
EHVAHLLFL R	P19652	Alpha-1-acid glycoprotein 2	9.98E+06	5.91E+06	1.30E+07	3.58E+06	7.25E+06	1.69	0.61	0.81	0.85	0.77	—	1.79	—	3.63	0.53	0.49	0.76
TLMFGSYL DDEK	P19652	Alpha-1-acid glycoprotein 2	1.97E+08	3.58E+08	3.47E+08	4.00E+07	1.28E+08	0.55	0.14	2.80	0.09	0.57	—	2.71	—	8.68	0.00	0.31	0.10
EQLGEFYEA LDCLCIPR	P19652	Alpha-1-acid glycoprotein 2	1.57E+08	1.07E+08	5.43E+08	7.01E+07	2.17E+08	1.47	0.32	0.49	0.07	0.29	—	2.50	—	7.74	0.08	0.32	0.21
LDQEVQEET QGR	P20138	Myeloid cell surface antigen CD33	6.61E+06	9.73E+06	9.52E+06	5.57E+06	7.62E+06	0.68	0.14	1.28	0.55	0.69	—	1.25	—	1.71	0.34	0.73	0.77
ILIPGTLEPG HSK	P20138	Myeloid cell surface antigen CD33	6.28E+06	8.40E+06	1.51E+07	7.65E+06	6.44E+06	0.75	0.20	1.31	0.22	0.42	—	2.35	—	1.98	0.26	1.19	0.69

LCCQMCEP GTFLVK	P26842	CD27 antigen	1.88E+08	2.01E+08	3.17E+08	1.49E+08	1.57E+08	0.94	0.77	1.28	0.33	0.59	—	2.02	—	2.13	0.23	0.95	0.91
NGWQCR	P26842	CD27 antigen	5.82E+07	7.57E+07	5.22E+07	2.96E+07	6.05E+07	0.77	0.38	1.25	0.56	1.12	—	0.86	—	1.76	0.32	0.49	0.22
DKECTECDP LPNPSLTAR	P26842	CD27 antigen	3.25E+07	3.44E+07	6.00E+07	4.34E+07	2.90E+07	0.94	0.84	1.19	0.51	0.54	—	2.07	—	1.38	0.60	1.50	0.36
TPILLIR	P28300	Protein-lysine 6-oxidase	2.25E+07	1.05E+07	1.65E+07	8.41E+06	2.03E+07	2.14	0.17	0.52	0.13	1.37	—	0.81	—	1.96	0.33	0.41	0.36
FIAVGYVDD TQFVR	P04439	HLA class I histocompatibility antigen, A alpha chain	3.27E+06	6.33E+06	7.52E+06	1.99E+06	3.77E+06	0.52	0.04	1.68	0.19	0.44	—	1.99	—	3.79	0.05	0.53	0.44
WAAVVVPS GEEQR	P04439	HLA class I histocompatibility antigen, A alpha chain	2.26E+07	2.22E+07	2.25E+07	3.66E+07	1.29E+07	1.02	0.96	1.72	0.24	1.00	—	1.74	—	0.61	0.33	2.83	0.02
TATITVLPQ QPR	P30530	Tyrosine-protein kinase receptor UFO	7.10E+08	5.90E+08	7.39E+08	6.28E+08	5.33E+08	1.20	0.36	1.11	0.61	0.96	—	1.39	—	1.18	0.73	1.18	0.66
APLQGTLLG YR	P30530	Tyrosine-protein kinase receptor UFO	2.96E+08	1.57E+08	3.11E+08	1.27E+08	2.37E+08	1.89	0.06	0.66	0.08	0.95	—	1.31	—	2.45	0.10	0.54	0.24
LAYQGQDT PEVLMDIGL R	P30530	Tyrosine-protein kinase receptor UFO	1.13E+08	7.91E+07	2.85E+08	1.30E+08	1.10E+08	1.42	0.27	0.72	0.43	0.39	—	2.58	—	2.19	0.26	1.18	0.75
SESVPPVTD WAWYK	P35613	Basigin	9.96E+06	1.07E+07	1.42E+07	1.44E+07	9.38E+06	0.93	0.76	1.14	0.55	0.70	—	1.52	—	0.99	0.98	1.54	0.20
FFVSSSQGR	P35613	Basigin	1.80E+07	2.10E+07	2.67E+07	2.38E+07	1.50E+07	0.86	0.43	1.40	0.14	0.67	—	1.78	—	1.12	0.84	1.59	0.17
GSDQAIITLR	P35613	Basigin	1.04E+07	1.52E+07	1.67E+07	2.07E+07	6.04E+06	0.69	0.29	2.51	0.04	0.63	—	2.76	—	0.81	0.80	3.42	0.06
IADGYEQAA R	P48643	T-complex protein 1 subunit epsilon	1.36E+06	2.38E+06	1.55E+06	2.14E+06	9.41E+05	0.57	0.23	2.53	0.12	0.88	—	1.65	—	0.72	0.76	2.28	0.26

LDVTSVED YK	P48643	T-complex protein 1 subunit epsilon	1.66E+06	1.67E+06	2.32E+06	2.61E+06	4.69E+05	1.00	0.99	3.56	0.03	0.72	—	4.95	—	0.89	0.92	5.57	0.06
QPPAWSIR	P49908	Selenoprotein P	2.60E+07	1.75E+07	2.54E+07	2.32E+06	3.16E+07	1.49	0.34	0.55	0.11	1.02	—	0.80	—	10.95	0.09	0.07	0.07
LDFHFSSDR	P55103	Inhibin beta C chain	1.25E+06	2.81E+06	1.86E+06	1.45E+06	1.37E+06	0.44	0.01	2.05	0.07	0.67	—	1.36	—	1.28	0.67	1.06	0.92
QILSADLR	P55786	Puromycin-sensitive aminopeptidase	1.86E+06	3.05E+06	6.71E+05	1.19E+06	9.92E+05	0.61	0.15	3.07	0.05	2.77	—	0.68	—	0.56	0.48	1.20	0.82
LDTGEYSCE AR	P57087	Junctional adhesion molecule [illegible]	5.82E+06	7.96E+06	6.71E+06	7.40E+06	4.49E+06	0.73	0.17	1.77	0.09	0.87	—	1.49	—	0.91	0.87	1.65	0.27
GYDVIAQA QSGTGK	P60842	Eukaryotic initiation factor 4A-I	1.32E+06	1.45E+06	1.02E+06	1.69E+06	4.61E+05	0.91	0.80	3.15	0.03	1.29	—	2.22	—	0.60	0.65	3.67	0.08
CVVVGDGA VGK	P60953	Cell division control protein 42 homolog	1.97E+07	3.01E+07	1.32E+07	2.28E+07	1.35E+07	0.65	0.10	2.23	0.02	1.49	—	0.98	—	0.58	0.38	1.69	0.18
NVFDEAILA ALEPPEPK	P60953	Cell division control protein 42 homolog	3.61E+06	2.26E+06	2.59E+06	7.66E+06	2.38E+06	1.60	0.28	0.95	0.88	1.39	—	1.09	—	0.34	0.34	3.22	0.05
DLMVGDEA SELR	P61160	Actin-related protein 2	3.92E+06	5.47E+06	3.56E+06	3.82E+06	1.67E+06	0.72	0.29	3.29	0.02	1.10	—	2.14	—	0.93	0.92	2.29	0.21
ILLTEPPMN PTK	P61160	Actin-related protein 2	3.01E+06	5.12E+06	2.48E+06	5.13E+06	2.33E+06	0.59	0.12	2.20	0.08	1.21	—	1.07	—	0.48	0.39	2.20	0.20
GLVIYPK	P98172	Ephrin-B1	1.79E+07	2.19E+07	1.68E+07	1.11E+07	9.86E+06	0.82	0.29	2.22	0.00	1.07	—	1.70	—	1.50	0.51	1.13	0.72
IGDKLDIICP R	P98172	Ephrin-B1	2.72E+06	1.62E+06	1.59E+05	3.06E+06	6.32E+05	1.67	0.69	2.57	0.42	17.12	—	0.25	—	0.05	0.25	4.84	0.19
SNPEDQILY QTER	Q14165	Malectin	6.10E+06	5.29E+06	3.87E+06	8.85E+06	4.79E+06	1.15	0.57	1.10	0.74	1.58	—	0.81	—	0.44	0.21	1.85	0.20
IEEVFK	Q14314	Fibroleukin	8.54E+06	1.16E+07	5.93E+06	6.14E+06	7.41E+06	0.74	0.20	1.56	0.10	1.44	—	0.80	—	0.97	0.98	0.83	0.73
EEINVLHGR	Q14314	Fibroleukin	6.54E+06	9.42E+06	5.82E+06	5.05E+06	7.38E+06	0.69	0.10	1.28	0.34	1.12	—	0.79	—	1.15	0.88	0.68	0.52

AGFGNLR	Q14314	Fibroleukin	4.39E+06	6.98E+06	2.52E+06	5.23E+06	2.53E+06	0.63	0.33	2.76	0.03	1.74	—	1.00	—	0.48	0.43	2.07	0.17
EGQGFVSED EYLEISDIKR	Q14982	Opioid-binding protein/cell adhesion molecule	1.75E+06	4.88E+06	1.04E+06	5.47E+06	5.26E+05	0.36	0.04	9.27	0.03	1.68	—	1.98	—	0.19	0.39	10.39	0.04
DQSGEYECS ALNDVAAP DVR	Q14982	Opioid-binding protein/cell adhesion molecule	7.15E+07	7.81E+07	6.35E+07	6.40E+07	5.65E+07	0.92	0.66	1.38	0.15	1.13	—	1.12	—	0.99	0.99	1.13	0.79
YGEGDSLTL QQLK	Q15043	Metal cation symporter ZIP14	2.69E+06	3.48E+06	6.18E+06	1.57E+06	2.04E+06	0.77	0.27	1.70	0.09	0.44	—	3.02	—	3.92	0.13	0.77	0.63
ALLNHLDV GVGR	Q15043	Metal cation symporter ZIP14	1.06E+06	9.96E+05	2.46E+06	3.66E+05	1.06E+06	1.06	0.85	0.94	0.82	0.43	—	2.32	—	6.72	0.01	0.35	0.11
AQLEQGGV GIR	Q6P1N0	Coiled-coil and C2 domain-containing protein 1A	1.95E+06	1.88E+06	3.49E+06	4.13E+06	1.22E+06	1.04	0.93	1.54	0.47	0.56	—	2.85	—	0.84	0.82	3.38	0.09
GSFSSTAAQ DAQGQR	Q86V85	Integral membrane protein GPR180	1.85E+07	2.23E+07	1.38E+07	1.45E+07	1.48E+07	0.83	0.40	1.51	0.06	1.34	—	0.93	—	0.96	0.90	0.98	0.96
LYLFQAQE WLK	Q86V85	Integral membrane protein GPR180	2.88E+05	8.18E+05	0.00	5.66E+03	1.67E+05	0.35	0.10	4.90	0.12	—	—	—	—	—	—	0.03	0.52
TEVIDNTLN PDFVR	Q86YQ8	Copine-8	4.23E+06	4.62E+06	3.94E+05	4.89E+02	9.97E+05	0.91	0.92	4.63	0.26	10.73	0.58	0.40	0.72	806.05	0.39	0.00	0.59
VEVYDWDR	Q86YQ8	Copine-8	4.39E+06	3.00E+06	3.43E+06	2.58E+06	2.90E+06	1.46	0.25	1.03	0.92	1.28	0.69	1.18	0.81	1.33	0.83	0.89	0.86
SQFSPAVFV YK	Q8WVV5	Butyrophilin subfamily 2 member A2	7.76E+07	8.42E+07	9.44E+07	6.55E+07	5.42E+07	0.92	0.69	1.55	0.05	0.82	0.57	1.74	0.11	1.44	0.48	1.21	0.62
ERTEEQMEE YR	Q8WVV5	Butyrophilin subfamily 2 member A2	1.04E+07	9.99E+06	1.07E+07	4.39E+06	7.90E+06	1.05	0.91	1.26	0.48	0.98	0.98	1.35	0.57	2.43	0.38	0.56	0.43

TEEQMEEYR	Q8WVV5	Butyrophilin subfamily 2 member A2	3.99E+07	4.47E+07	4.87E+07	2.01E+07	4.97E+07	0.89	0.67	0.90	0.77	0.82	0.63	0.98	0.97	2.43	0.19	0.40	0.34
ALADVATVLGR	Q92542	Nicastrin	2.89E+06	5.40E+06	3.92E+06	1.59E+06	1.11E+06	0.53	0.03	4.86	0.00	0.74	0.56	3.53	0.02	2.47	0.35	1.43	0.47
DSGPYSCSVNVQDK	Q96AP7	Endothelial cell-selective adhesion molecule	2.29E+07	3.89E+07	1.25E+07	5.10E+07	3.11E+07	0.59	0.03	1.25	0.65	1.83	0.34	0.40	0.55	0.24	0.11	1.64	0.58
TLELNVLVPPAPPSCR	Q96AP7	Endothelial cell-selective adhesion molecule	2.81E+07	4.25E+07	5.00E+07	3.93E+07	2.59E+07	0.66	0.04	1.64	0.04	0.56	0.09	1.93	0.10	1.27	0.72	1.52	0.19
SKPAVQYQWDR	Q96AP7	Endothelial cell-selective adhesion molecule	5.97E+06	1.51E+07	7.94E+06	3.90E+06	1.27E+07	0.40	0.00	1.19	0.68	0.75	0.47	0.63	0.63	2.04	0.35	0.31	0.41
HPPVQWAFQETSVESAVDTPFPAGIFVR	Q99969	Retinoic acid receptor responder protein 2	2.30E+06	1.27E+06	3.25E+06	0.00	1.41E+05	1.82	0.49	8.97	0.18	0.71	0.73	23.05	0.00	—	0.08	—	—
LVHCPIETQVLR	Q99969	Retinoic acid receptor responder protein 2	7.13E+06	1.55E+07	6.85E+06	2.19E+06	7.34E+06	0.46	0.04	2.11	0.11	1.04	0.95	0.93	0.92	3.12	0.21	0.30	0.35
FDCNFDLK	Q9H6Y7	E3 ubiquitin-protein ligase RNF167	7.82E+06	5.88E+06	1.14E+07	5.87E+06	6.49E+06	1.33	0.25	0.91	0.64	0.68	0.31	1.76	0.12	1.95	0.41	0.90	0.81
IEQLNADVFVPLR	Q9H756	Leucine-rich repeat-containing protein 19	2.10E+07	3.03E+07	2.15E+07	1.33E+07	1.44E+07	0.69	0.37	2.10	0.25	0.98	0.97	1.49	0.58	1.62	0.65	0.92	0.92
VFVSVLDVNDNAPEFPFK	Q9HBB8	Cadherin-related family member 5	4.16E+06	3.60E+06	1.27E+07	3.10E+06	4.08E+06	1.16	0.63	0.88	0.73	0.33	0.00	3.10	0.01	4.08	0.16	0.76	0.67

DAAAPSQPLR	Q9HBB8	Cadherin-related family member 5	3.65E+07	5.22E+07	4.39E+07	4.36E+07	3.96E+07	0.70	0.09	1.32	0.35	0.83	0.50	1.11	0.80	1.01	0.98	1.10	0.83
EYFGIVSVR	Q9NU53	Glycoprotein integral membrane protein 1	2.15E+07	3.28E+07	4.39E+07	2.14E+07	2.04E+07	0.66	0.04	1.61	0.04	0.49	0.03	2.15	0.03	2.05	0.28	1.05	0.91
LLPLEEHYR	Q9NZN3	EH domain-containing protein [illegible]	3.00E+06	1.90E+06	5.39E+06	3.33E+06	1.39E+06	1.58	0.27	1.37	0.45	0.56	0.28	3.88	0.05	1.62	0.64	2.39	0.14
ADQIETQQLMR	Q9NZN3	EH domain-containing protein [illegible]	9.60E+06	9.71E+06	8.96E+06	9.23E+06	6.32E+06	0.99	0.97	1.53	0.21	1.07	0.89	1.42	0.51	0.97	0.97	1.46	0.43
VYVSLPNEETR	Q9UBP0	Spastin	2.25E+06	1.56E+06	1.27E+06	7.80E+05	2.03E+06	1.44	0.40	0.77	0.63	1.76	0.50	0.63	0.66	1.63	0.75	0.38	0.48
SANPAMQLDR	Q9UBV8	Peflin	5.39E+06	5.53E+06	3.43E+06	1.00E+07	6.07E+06	0.97	0.93	0.91	0.76	1.57	0.52	0.56	0.41	0.34	0.14	1.65	0.31
SLTVSLGPVSK	Q9ULI3	Protein HEG homolog 1	2.70E+06	2.21E+06	8.82E+05	1.43E+06	3.57E+06	1.22	0.52	0.62	0.15	3.06	0.13	0.25	0.05	0.62	0.48	0.40	0.16
SIAGISYGQVR	Q9ULI3	Protein HEG homolog 1	2.12E+07	2.30E+07	1.62E+07	1.08E+07	1.96E+07	0.92	0.72	1.17	0.52	1.31	0.57	0.83	0.66	1.51	0.49	0.55	0.27
EIVSLLER	Q9ULK6	RING finger protein 150	4.08E+06	6.92E+06	1.17E+07	2.51E+06	5.14E+06	0.59	0.13	1.35	0.48	0.35	0.00	2.27	0.02	4.65	0.07	0.49	0.28
LIEFLSK	Q9Y376	Calcium-binding protein 39	1.45E+06	1.18E+06	1.47E+06	3.84E+05	1.18E+06	1.23	0.61	1.00	1.00	0.99	0.99	1.24	0.73	3.82	0.37	0.32	0.29
FSMDCAETAVLSNK	Q9Y4C0	Neurexin-3	7.91E+06	1.26E+07	1.58E+07	1.27E+07	9.89E+06	0.63	0.12	1.28	0.38	0.50	0.14	1.60	0.26	1.24	0.69	1.29	0.63
GLILDLK	Q9Y4C0	Neurexin-3	9.34E+06	1.32E+07	1.77E+07	8.13E+06	1.37E+07	0.71	0.11	0.97	0.90	0.53	0.03	1.29	0.43	2.17	0.14	0.60	0.32
DFLLSDK	Q9Y653	Adhesion G-protein coupled receptor G1	7.59E+06	9.66E+06	1.15E+07	8.74E+06	6.35E+06	0.79	0.26	1.52	0.07	0.66	0.25	1.81	0.09	1.31	0.67	1.38	0.37
RPSAAPASQQLQSLESK	Q9Y653	Adhesion G-protein coupled receptor G1	8.46E+05	5.49E+05	6.67E+05	6.80E+05	5.05E+05	1.54	0.51	1.09	0.91	1.27	0.82	1.32	0.79	0.98	0.99	1.35	0.79
