# Supplementary material for: Quantitative proteomics identifies and validates urinary biomarkers of rhabdomyosarcoma in children
Source: Clin Proteomics. 2023 Mar 14;20:10. doi: 10.1186/s12014-023-09401-4 (PMC10012572; doi:10.1186/s12014-023-09401-4)
Supplement: Supplementary file 7 — Additional file 7: Table S7. Literature analysis of 39 validated urinary biomarkers. [file 12014_2023_9401_MOESM7_ESM.pdf]

Table S7 Literature analysis of 39 validated urinary biomarkers.

ID	UniProt accession	Gene name	Protein name	Trend	Associated with RMS	Associated with muscular tissue	Reported as urine biomarkers	Reported as cancer biomarkers
1	P09237	MMP7	Matrilysin	Up	×	×	IgA[1], kidney fibrosis[2]	colon cancer[3], cervical cancer[4], gastric cancer[5], bladder cancer[6], ovarian cancer[7], oral verrucous and squamous cell cancer[8], head and neck squamous cell carcinomas[9]
2	Q13938	CAPS	Calcyphosin	Up	×	×	×	pediatric primitive neuroectodermal tumors[10], breast cancer[11], lung cancer[12]
3	P15907	ST6GAL1	Beta-galactoside alpha-2,6-sialyltransferase 1	Up	×	√[13]	×	hepatocellular carcinoma[14], gastric cancer[15], colorectal cancer[16]
4	P30086	PEBP1	Phosphatidylethanolamine-binding protein 1	Up	×	×	prostate cancer[17], ovarian cancer[18], clear cell renal cell carcinoma[19]	bladder cancer[20], pancreatic cancer[21], lung adenocarcinoma[22], hepatocellular carcinoma[23]
5	Q07075	ENPEP	Glutamyl aminopeptidase	Up	×	×	Familial Parkinson's disease[24]	colorectal cancer[25], breast cancer[26]
6	Q24JP5	TMEM132A	Transmembrane protein 132A	Up	×	×	×	×
7	Q9H6S3	EPS8L2	Epidermal growth factor receptor kinase substrate 8-like protein 2	Up	×	×	proliferative lupus nephritis[27], renal cell carcinoma[28]	endometrial carcinoma[29]
8	P01911	HLA-DRB1	HLA class II histocompatibility antigen, DRB1 beta chain	Up	×	×	renal allograft rejection[30]	×
9	P01009	SERPINA1	Alpha-1-antitrypsin	Up	×	×	preeclampsia[31], primary nephrotic syndrome[32], respiratory pathologies[33], bladder cancer[34], prostate cancer[35], morquio syndrome[36], hypertension[37]	bladder cancer[38], oral cancer[39], pancreatic cancer[40], non-small-cell lung cancer[41]
10	P02671	FGA	Fibrinogen alpha chain	Up	×	×	prostate cancer[17], bladder cancer[42], renal allograft rejection[30]	prostate cancer[17], bladder cancer[43]

11	O14745	SLC9A3R1	Na(+)/H(+) exchange regulatory cofactor NHE-RF1	Up	×	×	×	hepatocellular carcinoma[44]
12	Q10588	BST1	ADP-ribosyl cyclase/cyclic ADP-ribose hydrolase 2	Up	×	×	polycystic and other chronic kidney diseases[45], chronic lung allograft dysfunction[46]	prostate cancer[47]
13	O75309	CDH16	Cadherin-16	Up	×	×	Sepsis-induced acute kidney injury[48]	papillary thyroid cancer[49], lung adenocarcinoma[50]
14	P31997	CEACAM8	Carcinoembryonic antigen-related cell adhesion molecule 8	Up	×	×	×	oral squamous cell carcinoma[51], non-small cell lung cancer[52], gastric cancer[53]
15	Q9UKK9	NUDT5	ADP-sugar pyrophosphatase	Up	×	×	×	breast cancer[54], colon cancer[55]
16	Q9UN74	PCDHA4	Protocadherin alpha-4	Up	√[56]	×	×	cervical cancer[57]
17	Q13705	ACVR2B	Activin receptor type-2B	Down	×	√[58]	×	colorectal cancer[59]
18	Q6FHJ7	SFRP4	Secreted frizzled-related protein 4	Down	×	×	×	endometrial carcinoma[60], bladder cancer[61], gastric cancer[62], colon cancer[63]
19	O43278	SPINT1	Kunitz-type protease inhibitor 1	Down	×	×	urothelial carcinoma[64]	prostate adenocarcinoma[65], cervical cancer[66], endometrial carcinoma[67], CLL[68], pancreatic ductal adenocarcinoma[69]
20	O43291	SPINT2	Kunitz-type protease inhibitor 2	Down	×	×	×	cervical cancer[70], endometrial carcinoma[67], breast cancer[71], prostate cancer[72]
21	P08174	CD55	Complement decay-accelerating factor	Down	×	×	respiratory pathologies[33], prostate cancer[73]	colon cancer[74], primary gallbladder carcinoma[75], bladder cancer[76]
22	P09131	SLC10A3	P3 protein	Down	×	×	×	×
23	P09486	SPARC	SPARC	Down	×	×	clear cell renal cell carcinoma[77], urinary bladder cancer[78]	rectal cancer[79], non-small-cell lung cancer[80], pancreatic cancer[81], gastric cancer[82], hypopharyngeal cancer[83], colon cancer[84], diffuse large B-cell lymphoma[85], breast cancer[86]
24	P13598	ICAM2	Intercellular adhesion molecule 2	Down	×	×	×	lung cancer[87]

25	P15151	PVR	Poliovirus receptor	Down	×	×	×	multiple myeloma[88], bladder cancer[89]
26	P16112	ACAN	Aggrecan core protein	Down	×	×	×	gastric cancer[90], breast cancer[91]
27	P19022	CDH2	Cadherin-2	Down	×	×	respiratory pathologies[33], diabetic nephropathy[92]	malignant bone and soft tissue tumors[93], ductal carcinom[94], glioblastoma[95], papillary thyroid carcinoma[96]
28	P19652	ORM2	Alpha-1-acid glycoprotein 2	Down	×	×	rheumatoid arthritis[97], adult-onset Still's disease[98]	colorectal cancer[99], cervical cancer[100], cholangiocarcinoma[101], papillary thyroid carcinoma[102], liver cancer[103]
29	P20138	CD33	Myeloid cell surface antigen CD33	Down	×	×	×	colorectal cancer[104], AML[105]
30	P30530	AXL	Tyrosine-protein kinase receptor UFO	Down	√[106]	×	×	osteosarcoma[107], melanoma [108], esophageal adenocarcinoma [109], non-small cell lung cancer[110], colorectal adenocarcinoma[111] , Wilms' tumor[112]
31	Q15043	SLC39A14	Metal cation symporter ZIP14	Down	×	×	×	colorectal cancer[113], prostate cancer[114], breast cancer[115]
32	Q9HBB8	CDHR5	Cadherin-related family member 5	Down	×	×	autism[116]	hepatocellular carcinoma[117], clear cell renal cell carcinoma[118], pancreatic ductal adenocarcinoma[119]
33	Q9NU53	GINM1	Glycoprotein integral membrane protein 1	Down	×	×	bladder carcinoma[120]	bladder carcinoma[120]
34	Q9ULK6	RNF150	RING finger protein 150	Down	×	×	×	×
35	Q9Y4C0	NRXN3	Neurexin-3	Down	×	×	×	glioblastoma[121]
36	O75339	CILP	Cartilage intermediate layer protein 1	Down	×	×	cartilage matrix turnover[122]	bladder cancer[123]
37	Q9H6X2	ANTXR1	Anthrax toxin receptor 1	Down	×	×	×	angiosarcoma[124], gastric cancer[125], colorectal cancer[126], lung cancer[127]
38	P43490	NAMPT	Nicotinamide phosphoribosyltransferase	Down	√[128]	×	×	glioblastoma[129], bladder cancer[130], breast cancer[131], prostate cancer[132], basal cell carcinomas[133], breast invasive ductal carcinoma[134]

39	Q9BXN2	CLEC7A	C-type lectin domain family 7 member A	Down	×	×	×	clear cell renal cell carcinoma[135], AML[136]

References

1.Yang X, Ou J, Zhang H, Xu X, Zhu L, Li Q, et al. Urinary Matrix Metalloproteinase 7 and Prediction of IgA Nephropathy Progression. American journal of kidney diseases : the official journal of the National Kidney Foundation. 2020 2020 03;75(3):384-93. doi: 10.1053/j.ajkd.2019.07.018.

2.Zhou D, Tian Y, Sun L, Zhou L, Xiao L, Tan RJ, et al. Matrix Metalloproteinase-7 Is a Urinary Biomarker and Pathogenic Mediator of Kidney Fibrosis. Journal of the American Society of Nephrology : JASN. 2017 2017 02;28(2):598-611. doi: 10.1681/asn.2016030354.

3.Chen L, Ke X. MMP7 as a potential biomarker of colon cancer and its prognostic value by bioinformatics analysis. Medicine. 2021 2021 Mar 05;100(9):e24953. doi: 10.1097/md.0000000000024953.

4.Zhu L, Zheng X, Du Y, Xing Y, Xu K, Cui L. Matrix metalloproteinase-7 may serve as a novel biomarker for cervical cancer. OncoTargets and therapy. 2018 2018;11:4207-20. doi: 10.2147/ott.S160998.

5.Zhang Y, Qin L, Ma X, Wang Y, Wu Y, Jiang J. Coexpression of Matrix Metalloproteinase-7 and Tissue Inhibitor of Metalloproteinase-1 as a Prognostic Biomarker in Gastric Cancer. Disease markers. 2020 2020;2020:8831466. doi: 10.1155/2020/8831466.

6.Demery ME, Demirdjian-Sarkissian G, Thezenas S, Jacot W, Laghzali Y, Darbouret B, et al. Serum Matrix Metalloproteinase-7 is an independent prognostic biomarker in advanced bladder cancer. Clinical and translational medicine. 2014 2014;3:31. doi: 10.1186/s40169-014-0031-4.

7.Tanimoto H, Underwood LJ, Shigemasa K, Parmley TH, Wang Y, Yan Y, et al. The matrix metalloprotease pump-1 (MMP-7, Matrilysin): A candidate marker/target for ovarian cancer detection and treatment. Tumour biology : the journal of the International Society for Oncodevelopmental Biology and Medicine. 1999 1999 Mar-Apr;20(2):88-98. doi: 10.1159/000030051.

8.Impola U, Uitto VJ, Hietanen J, Hakkinen L, Zhang L, Larjava H, et al. Differential expression of matrilysin-1 (MMP-7), 92 kD gelatinase (MMP-9), and metalloelastase (MMP-12) in oral verrucous and squamous cell cancer. The Journal of pathology. 2004 2004 Jan;202(1):14-22. doi: 10.1002/path.1479.

9.Weber A, Hengge UR, Stricker I, Tischoff I, Markwart A, Anhalt K, et al. Protein microarrays for the detection of biomarkers in head and neck squamous cell carcinomas. Human pathology. 2007 2007 Feb;38(2):228-38. doi: 10.1016/j.humpath.2006.07.012.

10.Bont JMd, Boer MLd, Kros JM, Passier MMCJ, Reddingius RE, Smitt PAES, et al. Identification of novel biomarkers in pediatric primitive neuroectodermal tumors and ependymomas by proteome-wide analysis. Journal of neuropathology and experimental neurology. 2007 2007 Jun;66(6):505-16. doi: 10.1097/01.jnen.0000240475.35414.c3.

11.Johansson HJ, Sanchez BC, Forshed J, Stål O, Fohlin H, Lewensohn R, et al. Proteomics profiling identify CAPS as a potential predictive marker of tamoxifen resistance in estrogen receptor positive breast cancer. Clinical proteomics. 2015 2015;12(1):8. doi: 10.1186/s12014-015-9080-y.

12.Yang M, Sun Y, Sun J, Wang Z, Zhou Y, Yao G, et al. Differentially expressed and survival-related proteins of lung adenocarcinoma with bone metastasis. Cancer medicine. 2018 2018 04;7(4):1081-92. doi: 10.1002/cam4.1363.

13.Vergé C, Bouchatal A, Chirat F, Guérardel Y, Maftah A, Petit J-M. Involvement of ST6Gal I-mediated α2,6 sialylation in myoblast proliferation and differentiation. FEBS open bio. 2020 2020 01;10(1):56-69. doi: 10.1002/2211-5463.12745.

14.Myojin Y, Kodama T, Maesaka K, Motooka D, Sato Y, Tanaka S, et al. ST6GAL1 Is a Novel Serum Biomarker for Lenvatinib-Susceptible FGF19-Driven Hepatocellular Carcinoma. Clinical cancer research : an official journal of the American Association for Cancer Research. 2021 2021 02 15;27(4):1150-61. doi: 10.1158/1078-0432.Ccr-20-3382.

15.Duarte HO, Rodrigues JG, Gomes C, Hensbergen PJ, Ederveen ALH, Ru AHd, et al. ST6Gal1 targets the ectodomain of ErbB2 in a site-specific manner and regulates gastric cancer cell sensitivity to trastuzumab. Oncogene. 2021 2021 05;40(21):3719-33. doi: 10.1038/s41388-021-01801-w.

16.Costa-Nogueira C, Villar-Portela S, Cuevas E, Gil-Martín E, Fernández-Briera A. Synthesis and expression of CDw75 antigen in human colorectal cancer. BMC cancer. 2009 2009 Dec 10;9:431. doi: 10.1186/1471-2407-9-431.

17.Davalieva K, Kiprijanovska S, Kostovska IM, Stavridis S, Stankov O, Komina S, et al. Comparative Proteomics Analysis of Urine Reveals Down-Regulation of Acute Phase Response Signaling and LXR/RXR Activation Pathways in Prostate Cancer. Proteomes. 2017 2017 Dec 29;6(1). doi: 10.3390/proteomes6010001.
18.Rainczuk A, Condina M, Pelzing M, Dolman S, Rao J, Fairweather N, et al. The utility of isotope-coded protein labeling for prioritization of proteins found in ovarian cancer patient urine. Journal of proteome research. 2013 2013 Sep 06;12(9):4074-88. doi: 10.1021/pr400618v.
19.Papale M, Vocino G, Lucarelli G, Rutigliano M, Gigante M, Rocchetti MT, et al. Urinary RKIP/p-RKIP is a potential diagnostic and prognostic marker of clear cell renal cell carcinoma. Oncotarget. 2017 2017 Jun 20;8(25):40412-24. doi: 10.18632/oncotarget.16341.
20.Zaravinos A, Chatziioannou M, Lambrou GI, Boulalas I, Delakas D, Spandidos DA. Implication of RAF and RKIP genes in urinary bladder cancer. Pathology oncology research : POR. 2011 2011 Jun;17(2):181-90. doi: 10.1007/s12253-010-9295-1.
21.Qi Z-H, Xu H-X, Zhang S-R, Xu J-Z, Li S, Gao H-L, et al. RIPK4/PEBP1 axis promotes pancreatic cancer cell migration and invasion by activating RAF1/MEK/ERK signaling. International journal of oncology. 2018 2018 Apr;52(4):1105-16. doi: 10.3892/ijo.2018.4269.
22.Zhang A, Yang J, Ma C, Li F, Luo H. Development and Validation of a Robust Ferroptosis-Related Prognostic Signature in Lung Adenocarcinoma. Frontiers in cell and developmental biology. 2021 2021;9:616271. doi: 10.3389/fcell.2021.616271.
23.Xu Y-F, Yi Y, Qiu S-J, Gao Q, Li Y-W, Dai C-X, et al. PEBP1 downregulation is associated to poor prognosis in HCC related to hepatitis B infection. Journal of hepatology. 2010 2010 Nov;53(5):872-9. doi: 10.1016/j.jhep.2010.05.019.
24.Winter SV, Karayel O, Strauss MT, Padmanabhan S, Surface M, Merchant K, et al. Urinary proteome profiling for stratifying patients with familial Parkinson's disease. EMBO molecular medicine. 2021 2021 03 05;13(3):e13257. doi: 10.15252/emmm.202013257.
25.Yuan Y, Chen J, Wang J, Xu M, Zhang Y, Sun P, et al. Development and Clinical Validation of a Novel 4-Gene Prognostic Signature Predicting Survival in Colorectal Cancer. Frontiers in oncology. 2020 2020;10:595. doi: 10.3389/fonc.2020.00595.
26.Feliciano A, Castellvi J, Artero-Castro A, Leal JA, Romagosa C, Hernández-Losa J, et al. miR-125b acts as a tumor suppressor in breast tumorigenesis via its novel direct targets ENPEP, CK2-α, CCNJ, and MEGF9. PloS one. 2013 2013;8(10):e76247. doi: 10.1371/journal.pone.0076247.
27.Ghasemi M, Kalantari S, Zubarev RA, Nafar M, Saei AA, Heidari S-S, et al. Predictive Biomarker Panel in Proliferative Lupus Nephritis- Two-Dimensional Shotgun Proteomics. Iranian journal of kidney diseases. 2021 2021 Mar;1(2):121-33.
28.Meo AD, Batruch I, Brown MD, Yang C, Finelli A, Jewett MA, et al. Searching for prognostic biomarkers for small renal masses in the urinary proteome. International journal of cancer. 2020 2020 04 15;146(8):2315-25. doi: 10.1002/ijc.32650.
29.Kerley-Hamilton JS, Pike AM, Li N, DiRenzo J, Spinella MJ. A p53-dominant transcriptional response to cisplatin in testicular germ cell tumor-derived human embryonal carcinoma. Oncogene. 2005 2005 Sep 08;24(40):6090-100. doi: 10.1038/sj.onc.1208755.
30.Sigdel TK, Salomonis N, Nicora CD, Ryu S, He J, Dinh V, et al. The identification of novel potential injury mechanisms and candidate biomarkers in renal allograft rejection by quantitative proteomics. Molecular & cellular proteomics : MCP. 2014 2014 Feb;13(2):621-31. doi: 10.1074/mcp.M113.030577.
31.Starodubtseva N, Nizyaeva N, Baev O, Bugrova A, Gapaeva M, Muminova K, et al. SERPINA1 Peptides in Urine as A Potential Marker of Preeclampsia Severity. International journal of molecular sciences. 2020 2020 Jan 30;21(3). doi: 10.3390/ijms21030914.
32.Choi YW, Kim YG, Song M-Y, Moon J-Y, Jeong K-H, Lee T-W, et al. Potential urine proteomics biomarkers for primary nephrotic syndrome. Clinical proteomics. 2017 2017;14:18. doi: 10.1186/s12014-017-9153-1.
33.Starodubtseva NL, Kononikhin AS, Bugrova AE, Chagovets V, Indeykina M, Krokhina KN, et al. Investigation of urine proteome of preterm newborns with respiratory pathologies. Journal of proteomics. 2016 2016 10 21;149:31-7. doi: 10.1016/j.jprot.2016.06.012.
34.Chen L-M, Chang M, Dai Y, Chai KX, Dyrskjøt L, Sanchez-Carbayo M, et al. External validation of a multiplex urinary protein panel for the detection of bladder cancer in a multicenter cohort. Cancer epidemiology, biomarkers & prevention : a publication of the American Association for Cancer Research, cosponsored by the American Society of Preventive Oncology. 2014 2014 Sep;23(9):1804-12. doi: 10.1158/1055-9965.Epi-14-0029.
35.Davalieva K, Kiprijanovska S, Komina S, Petrusevska G, Zografska NC, Polenakovic M. Proteomics analysis of urine reveals acute phase response proteins as candidate diagnostic biomarkers for prostate cancer. Proteome science. 2015 2015;13(1):2. doi: 10.1186/s12953-014-0059-9.

36.Martell L, Lau K, Mei M, Burnett V, Decker C, Foehr ED. Biomarker analysis of Morquio syndrome: identification of disease state and drug responsive markers. Orphanet journal of rare diseases. 2011 2011 Dec 16;6:84. doi: 10.1186/1750-1172-6-84.

37.Gonzalez-Calero L, Martin-Lorenzo M, Cuesta Fdl, Maroto AS, Baldan-Martin M, Ruiz-Hurtado G, et al. Urinary alpha-1 antitrypsin and CD59 glycoprotein predict albuminuria development in hypertensive patients under chronic renin-angiotensin system suppression. Cardiovascular diabetology. 2016 2016 Jan 16;15:8. doi: 10.1186/s12933-016-0331-7.

38.Lindén M, Lind SB, Mayrhofer C, Segersten U, Wester K, Lyutvinskiy Y, et al. Proteomic analysis of urinary biomarker candidates for nonmuscle invasive bladder cancer. Proteomics. 2012 2012 Jan;12(1):135-44. doi: 10.1002/pmic.201000810.

39.Chu H-W, Chang K-P, Hsu C-W, Chang IY-F, Liu H-P, Chen Y-T, et al. Identification of Salivary Biomarkers for Oral Cancer Detection with Untargeted and Targeted Quantitative Proteomics Approaches. Molecular & cellular proteomics : MCP. 2019 2019 09;18(9):1796-806. doi: 10.1074/mcp.RA119.001530.

40.Wu C-C, Lu Y-T, Yeh T-S, Chan Y-H, Dash S, Yu J-S. Identification of Fucosylated SERPINA1 as a Novel Plasma Marker for Pancreatic Cancer Using Lectin Affinity Capture Coupled with iTRAQ-Based Quantitative Glycoproteomics. International journal of molecular sciences. 2021 2021 Jun 04;22(11). doi: 10.3390/ijms22116079.

41.Boccellino M, Pinto F, Ieluzzi V, Giovane A, Quagliuolo L, Fariello C, et al. Proteomics analysis of human serum of patients with non-small-cell lung cancer reveals proteins as diagnostic biomarker candidates. Journal of cellular physiology. 2019 2019 12;234(12):23798-806. doi: 10.1002/jcp.28948.

42.Awadalla A, Harraz AM, Abol-Enein H, Laymon M, Ahmed AE, Abdel-Rahim M, et al. Prognostic influence of microsatellite alterations of muscle-invasive bladder cancer treated with radical cystectomy. Urologic oncology. 2022 2022 02;40(2):64.e9-.e15. doi: 10.1016/j.urolonc.2021.08.020.

43.Zekri A-RN, Khaled HM, Mohammed MB, Diab FM, Abdellateif MS, Deeb SE, et al. Microsatellite instability profiling in Egyptian bladder cancer patients: A pilot study. Current problems in cancer. 2019 2019 12;43(6):100472. doi: 10.1016/j.currproblcancer.2019.03.002.

44.Chen W, Hu M-J, Zhong X-L, Ji L-H, Wang J, Zhang C-F, et al. Screening of a novel autophagy-related prognostic signature and therapeutic targets in hepatocellular carcinoma. Journal of gastrointestinal oncology. 2021 2021 Dec;12(6):2985-98. doi: 10.21037/jgo-21-664.

45.Konvalinka A, Batruch I, Tokar T, Dimitromanolakis A, Reid S, Song X, et al. Quantification of angiotensin II-regulated proteins in urine of patients with polycystic and other chronic kidney diseases by selected reaction monitoring. Clinical proteomics. 2016 2016;13:16. doi: 10.1186/s12014-016-9117-x.

46.Berra G, Farkona S, Mohammed-Ali Z, Kotlyar M, Levy L, Clotet-Freixas S, et al. Association between the renin-angiotensin system and chronic lung allograft dysfunction. The European respiratory journal. 2021 2021 10;58(4). doi: 10.1183/13993003.02975-2020.

47.Totten SM, Adusumilli R, Kullolli M, Tanimoto C, Brooks JD, Mallick P, et al. Multi-lectin Affinity Chromatography and Quantitative Proteomic Analysis Reveal Differential Glycoform Levels between Prostate Cancer and Benign Prostatic Hyperplasia Sera. Scientific reports. 2018 2018 04 25;8(1):6509. doi: 10.1038/s41598-018-24270-w.

48.Li Y, Long J, Chen J, Zhang J, Qin Y, Zhong Y, et al. Analysis of Spatiotemporal Urine Protein Dynamics to Identify New Biomarkers for Sepsis-Induced Acute Kidney Injury. Frontiers in physiology. 2020 2020;11:139. doi: 10.3389/fphys.2020.00139.

49.Calì G, Gentile F, Mogavero S, Pallante P, Nitsch R, Ciancia G, et al. CDH16/Ksp-cadherin is expressed in the developing thyroid gland and is strongly down-regulated in thyroid carcinomas. Endocrinology. 2012 2012 Jan;153(1):522-34. doi: 10.1210/en.2011-1572.

50.Li F, Wan B, Li X-Q. Expression Profile and Prognostic Values of CDH Family Members in Lung Adenocarcinoma. Disease markers. 2022 2022;2022:9644466. doi: 10.1155/2022/9644466.

51.Silva RNF, Dallarmi LB, Araujo AKC, Alencar RCG, Mendonça EF, Silva TA, et al. Immunohistochemical analysis of neutrophils, interleukin-17, matrix metalloproteinase-9, and neoformed vessels in oral squamous cell carcinoma. Journal of oral pathology & medicine : official publication of the International Association of Oral Pathologists and the American Academy of Oral Pathology. 2018 2018 Oct;47(9):856-63. doi:

52.Rakaee M, Busund L-T, Paulsen E-E, Richardsen E, Al-Saad S, Andersen S, et al. Prognostic effect of intratumoral neutrophils across histological subtypes of non-small cell lung cancer. Oncotarget. 2016 2016 11 01;7(44):72184-96. doi: 10.18632/oncotarget.12360.

53.Li S, Sun S, Sun H, Ma P, Zhang J, Cao Y, et al. A risk signature with inflammatory and immune cells infiltration predicts survival and efficiency of chemotherapy in gastric cancer. International immunopharmacology. 2021 2021 Jul;96:107589. doi: 10.1016/j.intimp.2021.107589.

54.Tong X-Y, Quan Y, Zhang H-Y. NUDT5 as a novel drug target and prognostic biomarker for ER-positive breast cancer. Drug discovery today. 2021 2021 03;26(3):620-5. doi: 10.1016/j.drudis.2020.11.031.

55.Li J, Yang C-C, Tian X-Y, Li Y-X, Cui J, Chen Z, et al. MutT-related proteins are novel progression and prognostic markers for colorectal cancer. Oncotarget. 2017 2017 Dec 01;8(62):105714-26. doi: 10.18632/oncotarget.22393.

56.Tombolan L, Poli E, Martini P, Zin A, Millino C, Pacchioni B, et al. Global DNA methylation profiling uncovers distinct methylation patterns of protocadherin alpha4 in metastatic and non-metastatic rhabdomyosarcoma. BMC cancer. 2016 2016 11 14;16(1):886. doi: 10.1186/s12885-016-2936-3.

57.Wang K-H, Lin C-J, Liu C-J, Liu D-W, Huang R-L, Ding D-C, et al. Global methylation silencing of clustered proto-cadherin genes in cervical cancer: serving as diagnostic markers comparable to HPV. Cancer medicine. 2015 2015 Jan;4(1):43-55. doi: 10.1002/cam4.335.

58.Walsh S, Metter EJ, Ferrucci L, Roth SM. Activin-type II receptor B (ACVR2B) and follistatin haplotype associations with muscle mass and strength in humans. Journal of applied physiology (Bethesda, Md : 1985). 2007 2007 Jun;102(6):2142-8. doi: 10.1152/japplphysiol.01322.2006.

59.Huot JR, Pin F, Narasimhan A, Novinger LJ, Keith AS, Zimmers TA, et al. ACVR2B antagonism as a countermeasure to multi-organ perturbations in metastatic colorectal cancer cachexia. Journal of cachexia, sarcopenia and muscle. 2020 2020 12;11(6):1779-98. doi: 10.1002/jcsm.12642.

60.Hrzenjak A, Tippl M, Kremser M-L, Strohmeier B, Guelly C, Neumeister D, et al. Inverse correlation of secreted frizzled-related protein 4 and beta-catenin expression in endometrial stromal sarcomas. The Journal of pathology. 2004 2004 Sep;204(1):19-27. doi: 10.1002/path.1616.

61.Serizawa RR, Ralfkiaer U, Steven K, Lam GW, Schmiedel S, Schüz J, et al. Integrated genetic and epigenetic analysis of bladder cancer reveals an additive diagnostic value of FGFR3 mutations and hypermethylation events. International journal of cancer. 2011 2011 Jul 01;129(1):78-87. doi: 10.1002/ijc.25651.

62.Cheong J-H, Yang H-K, Kim H, Kim WH, Kim Y-W, Kook M-C, et al. Predictive test for chemotherapy response in resectable gastric cancer: a multi-cohort, retrospective analysis. The Lancet Oncology. 2018 2018 05;19(5):629-38. doi: 10.1016/s1470-2045(18)30108-6.

63.Nfonsam LE, Jandova J, Jecius HC, Omesiete PN, Nfonsam VN. SFRP4 expression correlates with epithelial mesenchymal transition-linked genes and poor overall survival in colon cancer patients. World journal of gastrointestinal oncology. 2019 2019 Aug 15;11(8):589-98. doi: 10.4251/wjgo.v11.i8.589.

64.Shimwell NJ, Bryan RT, Wei W, James ND, Cheng KK, Zeegers MP, et al. Combined proteome and transcriptome analyses for the discovery of urinary biomarkers for urothelial carcinoma. British journal of cancer. 2013 2013 May 14;108(9):1854-61. doi: 10.1038/bjc.2013.157.

65.Saleem M, Adhami VM, Zhong W, Longley BJ, Lin C-Y, Dickson RB, et al. A novel biomarker for staging human prostate adenocarcinoma: overexpression of matriptase with concomitant loss of its inhibitor, hepatocyte growth factor activator inhibitor-1. Cancer epidemiology, biomarkers & prevention : a publication of the American Association for Cancer Research, cosponsored by the American Society of Preventive Oncology. 2006 2006

66.Nakamura K, Abarzua F, Hongo A, Kodama J, Nasu Y, Kumon H, et al. The role of hepatocyte growth factor activator inhibitor-1 (HAI-1) as a prognostic indicator in cervical cancer. International journal of oncology. 2009 2009 Aug;35(2):239-48.

67.Nakamura K, Hongo A, Kodama J, Hiramatsu Y. The role of hepatocyte growth factor activator inhibitor (HAI)-1 and HAI-2 in endometrial cancer. International journal of cancer. 2011 2011 Jun 01;128(11):2613-24. doi: 10.1002/ijc.25606.

68.Hosnijeh FS, Straten Lvd, Kater AP, Oers MHJv, Posthuma WFM, Chamuleau MED, et al. Proteomic markers with prognostic impact on outcome of chronic lymphocytic leukemia patients under chemo-immunotherapy: results from the HOVON 109 study. Experimental hematology. 2020 2020 09;89:55-60.e6. doi: 10.1016/j.exphem.2020.08.002.

69.Sakugawa C, Haruyama Y, Tanaka H, Fukushima T, Kawaguchi M, Kataoka H. Prognostic significance of hepatocyte growth factor activator inhibitor type 1 (HAI-1) immunoreactivity in pancreatic ductal adenocarcinoma. BMC research notes. 2017 2017 Dec 04;10(1):674. doi: 10.1186/s13104-017-3014-x.

70.Nakamura K, Abarzua F, Hongo A, Kodama J, Nasu Y, Kumon H, et al. Hepatocyte growth factor activator inhibitor-2 (HAI-2) is a favorable prognosis marker and inhibits cell growth through the apoptotic pathway in cervical cancer. Annals of oncology : official journal of the European Society for Medical Oncology. 2009 2009 Jan;20(1):63-70. doi: 10.1093/annonc/mdn556.

71.Generali D, Fox SB, Berruti A, Moore JW, Brizzi MP, Patel N, et al. Regulation of hepatocyte growth factor activator inhibitor 2 by hypoxia in breast cancer. Clinical cancer research : an official journal of the American Association for Cancer Research. 2007 2007 Jan 15;13(2 Pt 1):550-8. doi: 10.1158/1078-0432.Ccr-06-1266.

72.Wu L, Shu X, Bao J, Guo X, Kote-Jarai Z, Haiman CA, et al. Analysis of Over 140,000 European Descendants Identifies Genetically Predicted Blood Protein Biomarkers Associated with Prostate Cancer Risk. Cancer research. 2019 2019 09 15;79(18):4592-8. doi: 10.1158/0008-5472.Can-18-3997.

73.Lima T, Barros AS, Trindade F, Ferreira R, Leite-Moreira A, Barros-Silva D, et al. Application of Proteogenomics to Urine Analysis towards the Identification of Novel Biomarkers of Prostate Cancer: An Exploratory Study. Cancers. 2022 2022 Apr 15;14(8). doi: 10.3390/cancers14082001.

74.Durrant LG, Chapman MA, Buckley DJ, Spendlove I, Robins RA, Armitage NC. Enhanced expression of the complement regulatory protein CD55 predicts a poor prognosis in colorectal cancer patients. Cancer immunology, immunotherapy : CII. 2003 2003 Oct;52(10):638-42. doi: 10.1007/s00262-003-0402-y.

75.Wu J, Lei L, Wang S, Gu D, Zhang J. Immunohistochemical expression and prognostic value of CD97 and its ligand CD55 in primary gallbladder carcinoma. Journal of biomedicine & biotechnology. 2012 2012;2012:587672. doi: 10.1155/2012/587672.

76.Varela JC, Atkinson C, Woolson R, Keane TE, Tomlinson S. Upregulated expression of complement inhibitory proteins on bladder cancer cells and anti-MUC1 antibody immune selection. International journal of cancer. 2008 2008 Sep 15;123(6):1357-63. doi: 10.1002/ijc.23676.

77.Senturk A, Sahin AT, Armutlu A, Kiremit MC, Acar O, Erdem S, et al. Quantitative Proteomics Identifies Secreted Diagnostic Biomarkers as well as Tumor-Dependent Prognostic Targets for Clear Cell Renal Cell Carcinoma. Molecular cancer research : MCR. 2021 2021 08;19(8):1322-37. doi: 10.1158/1541-7786.Mcr-21-0004.

78.Critselis E, Rava M, Marquez M, Lygirou V, Chatzicharalambous D, Liapi P, et al. Diagnostic and Prognostic Performance of Secreted Protein Acidic and Rich in Cysteine (SPARC) Assay for Detecting Primary and Recurrent Urinary Bladder Cancer. Proteomics Clinical applications. 2019 2019 03;13(2):e1800148. doi: 10.1002/prca.201800148.

79.Kurtul N, Taşdemir EA, Ünal D, İzmirli M, Eroglu C. SPARC: As a prognostic biomarker in rectal cancer patients treated with chemo-radiotherapy. Cancer biomarkers : section A of Disease markers. 2017 2017;18(4):459-66. doi: 10.3233/cbm-161733.

80.Fabrizio FP, Sparaneo A, Fontana A, Mazza T, Graziano P, Pantalone A, et al. Potential Prognostic Role of SPARC Methylation in Non-Small-Cell Lung Cancer. Cells. 2020 2020 06 22;9(6). doi: 10.3390/cells9061523.

81.Gundewar C, Sasor A, Hilmersson KS, Andersson R, Ansari D. The role of SPARC expression in pancreatic cancer progression and patient survival. Scandinavian journal of gastroenterology. 2015 2015;50(9):1170-4. doi: 10.3109/00365521.2015.1024281.

82.Shan Z, Wang W, Tong Y, Zhang J. Genome-Scale Analysis Identified NID2, SPARC, and MFAP2 as Prognosis Markers of Overall Survival in Gastric Cancer. Medical science monitor : international medical journal of experimental and clinical research. 2021 2021 Mar 24;27:e929558. doi: 10.12659/msm.929558.

83.Ernst BP, Mikstas C, Stöver T, Stauber R, Strieth S. Association of eIF4E and SPARC Expression with Lymphangiogenesis and Lymph Node Metastasis in Hypopharyngeal Cancer. Anticancer research. 2018 2018 02;38(2):699-706. doi: 10.21873/anticanres.12275.

84.Zhong M-E, Chen Y, Xiao Y, Xu L, Zhang G, Lu J, et al. Serum extracellular vesicles contain SPARC and LRG1 as biomarkers of colon cancer and differ by tumour primary location. EBioMedicine. 2019 2019 Dec;50:211-23. doi: 10.1016/j.ebiom.2019.11.003.

85.Pan P-J, Liu J-X. Diagnostic and prognostic value of secreted protein acidic and rich in cysteine in the diffuse large B-cell lymphoma. World journal of clinical cases. 2021 2021 Aug 06;9(22):6287-99. doi: 10.12998/wjcc.v9.i22.6287.

86.Nagai MA, Gerhard R, Fregnani JHTG, Nonogaki S, Rierger RB, Netto MM, et al. Prognostic value of NDRG1 and SPARC protein expression in breast cancer patients. Breast cancer research and treatment. 2011 2011 Feb;126(1):1-14. doi: 10.1007/s10549-010-0867-2.

87.Li J, Yu H, Ma Y-F, Zhao M, Tang J. Identification of genes associated with lung cancer by bioinformatics analysis. European review for medical and pharmacological sciences. 2017 2017 05;21(10):2397-404.

88.Lee B-H, Kim J-H, Kang K-W, Lee S-R, Park Y, Sung H-J, et al. PVR (CD155) Expression as a Potential Prognostic Marker in Multiple Myeloma. Biomedicines. 2022 2022 May 10;10(5). doi: 10.3390/biomedicines10051099.

89.Luo C, Ye W, Hu J, Othmane B, Li H, Chen J, et al. A Poliovirus Receptor (CD155)-Related Risk Signature Predicts the Prognosis of Bladder Cancer. Frontiers in oncology. 2021 2021;11:660273. doi: 10.3389/fonc.2021.660273.

90.Vafaeie F, Nomiri S, Ranjbaran J, Safarpour H. ACAN, MDFI, and CHST1 as Candidate Genes in Gastric Cancer: A Comprehensive Insilco Analysis. Asian Pacific journal of cancer prevention : APJCP. 2022 2022 Feb 01;23(2):683-94. doi: 10.31557/apjcp.2022.23.2.683.

91.Yang Y-S, Ren Y-X, Liu C-L, Hao S, Xu X-E, Jin X, et al. The early-stage triple-negative breast cancer landscape derives a novel prognostic signature and therapeutic target. Breast cancer research and treatment. 2022 2022 Jun;193(2):319-30. doi: 10.1007/s10549-022-06537-z.

92.Zheng M, Lv L-L, Cao Y-H, Liu H, Ni J, Dai H-Y, et al. A pilot trial assessing urinary gene expression profiling with an mRNA array for diabetic nephropathy. PloS one. 2012 2012;7(5):e34824. doi: 10.1371/journal.pone.0034824.

93.Niimi R, Matsumine A, Iino T, Nakazora S, Nakamura T, Uchida A, et al. Soluble Neural-cadherin as a novel biomarker for malignant bone and soft tissue tumors. BMC cancer. 2013 2013 Jun 26;13:309. doi: 10.1186/1471-2407-13-309.

94.Guvakova MA, Prabakaran I, Wu Z, Hoffman DI, Huang Y, Tchou J, et al. CDH2/N-cadherin and early diagnosis of invasion in patients with ductal carcinoma in situ. Breast cancer research and treatment. 2020 2020 Sep;183(2):333-46. doi: 10.1007/s10549-020-05797-x.

95.Chen Q, Cai J, Jiang C. CDH2 expression is of prognostic significance in glioma and predicts the efficacy of temozolomide therapy in patients with glioblastoma. Oncology letters. 2018 2018 May;15(5):7415-22. doi: 10.3892/ol.2018.8227.

96.Wan Y, Zhang X, Leng H, Yin W, Zeng W, Zhang C. Identifying hub genes of papillary thyroid carcinoma in the TCGA and GEO database using bioinformatics analysis. PeerJ. 2020 2020;8:e9120. doi: 10.7717/peerj.9120.

97.Park Y-J, Yoo S-A, Hwang D, Cho C-S, Kim W-U. Identification of novel urinary biomarkers for assessing disease activity and prognosis of rheumatoid arthritis. Experimental & molecular medicine. 2016 2016 Feb 26;48:e211. doi: 10.1038/emm.2015.120.

98.Sun Y, Wang F, Zhou Z, Teng J, Su Y, Chi H, et al. Urinary Proteomics Identifying Novel Biomarkers for the Diagnosis of Adult-Onset Still's Disease. Frontiers in immunology. 2020 2020;11:2112. doi: 10.3389/fimmu.2020.02112.

99.Kopylov AT, Stepanov AA, Malsagova KA, Soni D, Kushlinsky NE, Enikeev DV, et al. Revelation of Proteomic Indicators for Colorectal Cancer in Initial Stages of Development. Molecules (Basel, Switzerland). 2020 2020 Jan 31;25(3). doi: 10.3390/molecules25030619.

100.Qiu F, Chen F, Liu D, Xu J, He J, Xiao J, et al. [LC-MS/MS-based screening of new protein biomarkers for cervical precancerous lesions and cervical cancer]. Nan fang yi ke da xue xue bao = Journal of Southern Medical University. 2019 2019 Jan 30;39(1):13-22. doi: 10.12122/j.issn.1673-4254.2019.01.03.

101.Rucksaken R, Charoensuk L, Pinlaor P, Pairojkul C, Khuntikeo N, Pinlaor S. Plasma orosomucoid 2 as a potential risk marker of cholangiocarcinoma. Cancer biomarkers : section A of Disease markers. 2017 2017;18(1):27-34. doi: 10.3233/cbm-160670.

102.Yekta RF, Oskouie AA, Tavirani MR, Mohajeri-Tehrani MR, Soroush AR. Decreased apolipoprotein A4 and increased complement component 3 as potential markers for papillary thyroid carcinoma: A proteomic study. The International journal of biological markers. 2018 2018 Nov;33(4):455-62. doi: 10.1177/1724600818787752.

103.Zhu H-Z, Zhou W-J, Wan Y-F, Ge K, Lu J, Jia C-K. Downregulation of orosomucoid 2 acts as a prognostic factor associated with cancer-promoting pathways in liver cancer. World journal of gastroenterology. 2020 2020 Feb 28;26(8):804-17. doi: 10.3748/wjg.v26.i8.804.

104.Toor SM, Taha RZ, Nair VS, Saleh R, Murshed K, Nada MA, et al. Differential gene expression of tumor-infiltrating CD33+ myeloid cells in advanced- versus early-stage colorectal cancer. Cancer immunology, immunotherapy : CII. 2021 2021 Mar;70(3):803-15. doi: 10.1007/s00262-020-02727-0.

105.Willier S, Rothämel P, Hastreiter M, Wilhelm J, Stenger D, Blaeschke F, et al. CLEC12A and CD33 coexpression as a preferential target for pediatric AML combinatorial immunotherapy. Blood. 2021 2021 02 25;137(8):1037-49. doi: 10.1182/blood.2020006921.

106.Bai Y, Li J, Fang B, Edwards A, Zhang G, Bui M, et al. Phosphoproteomics identifies driver tyrosine kinases in sarcoma cell lines and tumors. Cancer research. 2012 2012 May 15;72(10):2501-11. doi: 10.1158/0008-5472.Can-11-3015.

107.Li Q, Wang X, Jiang N, Xie X, Liu N, Liu J, et al. Exosome-transmitted linc00852 associated with receptor tyrosine kinase AXL dysregulates the proliferation and invasion of osteosarcoma. Cancer medicine. 2020 2020 09;9(17):6354-66. doi: 10.1002/cam4.3303.

108.Flem-Karlsen K, Nyakas M, Farstad IN, McFadden E, Wernhoff P, Jacobsen KD, et al. Soluble AXL as a marker of disease progression and survival in melanoma. PloS one. 2020 2020;15(1):e0227187. doi: 10.1371/journal.pone.0227187.

109.Hong J, Abid F, Phillips S, Salaria SN, Revetta FL, Peng D, et al. Co-overexpression of AXL and c-ABL predicts a poor prognosis in esophageal adenocarcinoma and promotes cancer cell survival. Journal of Cancer. 2020 2020;11(20):5867-79. doi: 10.7150/jca.47318.

110.Ramkumar K, Stewart CA, Cargill KR, Corte CMD, Wang Q, Shen L, et al. AXL Inhibition Induces DNA Damage and Replication Stress in Non-Small Cell Lung Cancer Cells and Promotes Sensitivity to ATR Inhibitors. Molecular cancer research : MCR. 2021 2021 03;19(3):485-97. doi: 10.1158/1541-7786.Mcr-20-0414.

111.Ciardiello D, Blauensteiner B, Matrone N, Belli V, Mohr T, Vitiello PP, et al. Dual inhibition of TGFβ and AXL as a novel therapy for human colorectal adenocarcinoma with mesenchymal phenotype. Medical oncology (Northwood, London, England). 2021 2021 Feb 11;38(3):24. doi: 10.1007/s12032-021-01464-3.

112.Zhu S, Liu G, Fu W, Hu J, Fu K, Jia W. Axl promotes the proliferation, invasion and migration of Wilms' tumor and can be used as a prognostic factor. OncoTargets and therapy. 2017 2017;10:955-63. doi: 10.2147/ott.S127419.

113.Thorsen K, Mansilla F, Schepeler T, Øster B, Rasmussen MH, Dyrskjøt L, et al. Alternative splicing of SLC39A14 in colorectal cancer is regulated by the Wnt pathway. Molecular & cellular proteomics : MCP. 2011 2011 Jan;10(1):M110.002998. doi: 10.1074/mcp.M110.002998.

114.Shakri AR, Zhong TJ, Ma W, Coker C, Kim S, Calluori S, et al. Upregulation of ZIP14 and Altered Zinc Homeostasis in Muscles in Pancreatic Cancer Cachexia. Cancers. 2019 2019 Dec 18;12(1). doi: 10.3390/cancers12010003.

115.Liu L, Yang J, Wang C. Analysis of the prognostic significance of solute carrier (SLC) family 39 genes in breast cancer. Bioscience reports. 2020 2020 08 28;40(8). doi: 10.1042/bsr20200764.

116.Meng W, Huan Y, Gao Y. Urinary proteome profiling for children with autism using data-independent acquisition proteomics. Translational pediatrics. 2021 2021 Jul;10(7):1765-78. doi: 10.21037/tp-21-193.

117.Ding X, Tian X, Liu W, Li Z. CDHR5 inhibits proliferation of hepatocellular carcinoma and predicts clinical prognosis. Irish journal of medical science. 2020 2020 May;189(2):439-47. doi: 10.1007/s11845-019-02092-7.

118.Bläsius FM, Meller S, Stephan C, Jung K, Ellinger J, Glocker MO, et al. Loss of cadherin related family member 5 (CDHR5) expression in clear cell renal cell carcinoma is a prognostic marker of disease progression. Oncotarget. 2017 2017 Sep 26;8(43):75076-86. doi: 10.18632/oncotarget.20507.

119.Gao J, Wang M, Li T, Liu Q, You L, Liao Q. Up-regulation of CDHR5 expression promotes malignant phenotype of pancreatic ductal adenocarcinoma. Journal of cellular and molecular medicine. 2020 2020 11;24(21):12726-35. doi: 10.1111/jcmm.15856.

120.Sathe G, George IA, Deb B, Jain AP, Patel K, Nayak B, et al. Urinary glycoproteomic profiling of non-muscle invasive and muscle invasive bladder carcinoma patients reveals distinct N-glycosylation pattern of CD44, MGAM, and GINM1. Oncotarget. 2020 2020 Aug 25;11(34):3244-55. doi: 10.18632/oncotarget.27696.

121.Yang Q, Wang R, Wei B, Peng C, Wang L, Hu G, et al. Gene and microRNA Signatures Are Associated with the Development and Survival of Glioblastoma Patients. DNA and cell biology. 2019 2019 Jul;38(7):688-99. doi: 10.1089/dna.2018.4353.

122.Boeth H, Raffalt PC, MacMahon A, Poole AR, Eckstein F, Wirth W, et al. Association between changes in molecular biomarkers of cartilage matrix turnover and changes in knee articular cartilage: a longitudinal pilot study. Journal of experimental orthopaedics. 2019 2019 May 03;6(1):19. doi: 10.1186/s40634-019-0179-3.

123.Tan Z, Fu S, Feng R, Huang Y, Li N, Wang H, et al. Identification of Potential Biomarkers for Progression and Prognosis of Bladder Cancer by Comprehensive Bioinformatics Analysis. Journal of oncology. 2022 2022;2022:1802706. doi: 10.1155/2022/1802706.

124.Kusaba Y, Kajihara I, Sakamoto R, Maeda-Otsuka S, Yamada-Kanazawa S, Sawamura S, et al. Overexpression of tumor endothelial marker 8 protein predicts poor prognosis in angiosarcoma. The Journal of dermatology. 2021 2021 Oct;48(10):E514-E6. doi: 10.1111/1346-8138.16069.

125.Huang X, Zhang J, Zheng Y. ANTXR1 Is a Prognostic Biomarker and Correlates With Stromal and Immune Cell Infiltration in Gastric Cancer. Frontiers in molecular biosciences. 2020 2020;7:598221. doi: 10.3389/fmolb.2020.598221.

126.Pietrzyk Ł, Korolczuk A, Matysek M, Arciszewski MB, Torres K. Clinical Value of Detecting Tumor Endothelial Marker 8 (ANTXR1) as a Biomarker in the Diagnosis and Prognosis of Colorectal Cancer. Cancer management and research. 2021 2021;13:3113-22. doi: 10.2147/cmar.S298165.

127.Sun M, Li H, Liu J, Ning L, Zhao D, Liu S. The relationship between TEM8 and early diagnosis and prognosis of lung cancer. Minerva medica. 2021 2021 Jun;112(3):359-64. doi: 10.23736/s0026-4806.20.06444-7.

128.Vora M, Ansari J, Shanti RM, Veillon D, Cotelingam J, Coppola D, et al. Increased Nicotinamide Phosphoribosyltransferase in Rhabdomyosarcomas and Leiomyosarcomas Compared to Skeletal and Smooth Muscle Tissue. Anticancer research. 2016 2016 Feb;36(2):503-7.

129.Guo Q, Han N, Shi L, Yang L, Zhang X, Zhou Y, et al. NAMPT: A potential prognostic and therapeutic biomarker in patients with glioblastoma. Oncology reports. 2019 2019 Sep;42(3):963-72. doi: 10.3892/or.2019.7227.

130.Elamin AA, Klunkelfuß S, Kämpfer S, Oehlmann W, Stehr M, Smith C, et al. A Specific Blood Signature Reveals Higher Levels of S100A12: A Potential Bladder Cancer Diagnostic Biomarker Along With Urinary Engrailed-2 Protein Detection. Frontiers in oncology. 2019 2019;9:1484. doi: 10.3389/fonc.2019.01484.

131.Zhu Y, Guo M, Zhang L, Xu T, Wang L, Xu G. Biomarker triplet NAMPT/VEGF/HER2 as a de novo detection panel for the diagnosis and prognosis of human breast cancer. Oncology reports. 2016 2016 Jan;35(1):454-62. doi: 10.3892/or.2015.4391.
132.Sun BL, Sun X, Casanova N, Garcia AN, Oita R, Algotar AM, et al. Role of secreted extracellular nicotinamide phosphoribosyltransferase (eNAMPT) in prostate cancer progression: Novel biomarker and therapeutic target. EBioMedicine. 2020 2020 Nov;61:103059. doi: 10.1016/j.ebiom.2020.103059.
133.Brandl L, Hartmann D, Kirchner T, Menssen A. Expression of n-MYC, NAMPT and SIRT1 in Basal Cell Carcinomas and their Cells of Origin. Acta dermato-venereologica. 2019 2019 01 01;99(1):63-71. doi: 10.2340/00015555-3031.
134.Zhou S-J, Bi T-Q, Qin C-X, Yang X-Q, Pang K. Expression of NAMPT is associated with breast invasive ductal carcinoma development and prognosis. Oncology letters. 2018 2018 May;15(5):6648-54. doi: 10.3892/ol.2018.8164.
135.Xia Y, Liu L, Bai Q, Wang J, Xi W, Qu Y, et al. Dectin-1 predicts adverse postoperative prognosis of patients with clear cell renal cell carcinoma. Scientific reports. 2016 2016 09 07;6:32657. doi: 10.1038/srep32657.
136.Zhang H, Nakauchi Y, Köhnke T, Stafford M, Bottomly D, Thomas R, et al. Integrated analysis of patient samples identifies biomarkers for venetoclax efficacy and combination strategies in acute myeloid leukemia. Nature cancer. 2020 2020 08;1(8):826-39. doi: 10.1038/s43018-020-0103-x.
