# Supplementary material for: Quantitative proteomics identifies and validates urinary biomarkers of rhabdomyosarcoma in children
Source: Clin Proteomics. 2023 Mar 14;20:10. doi: 10.1186/s12014-023-09401-4 (PMC10012572; doi:10.1186/s12014-023-09401-4)
Supplement: Supplementary file 8 — Additional file 8: Table S8. Biomarkers panel for diagnosis of RMS. [file 12014_2023_9401_MOESM8_ESM.pdf]

Table S8 Biomarkers panel for diagnosis of RMS.

ID	Peptides Name	UniProt accession	Gene name	Protein name	AUC	T-tests
1	SQPVSQPLTYESGPDEVR	Q9H6S3	EPS8L2	Epidermal growth factor receptor kinase substrate 8-like protein 2	0.73	0.001
2	LHLDYIGPCK	P09486	SPARC	Secreted protein acidic and rich in cysteine	0.69	0.005
3	FDSDVGEFR	P01911	HLA-DRB1	HLA class II histocompatibility antigen, DRB1 beta chain	0.68	0.04
4	FTFQEAANECR	P16112	ACAN	Aggrecan core protein	0.67	0.04
5	TFLVGNLEIR	O75339	CILP	Cartilage intermediate layer protein 1	0.67	0.04
